# Pan-GWAS of Streptococcus agalactiae Highlights Lineage-Specific Genes Associated with Virulence and Niche Adaptation

**DOI:** 10.1128/mBio.00728-20

**Published:** 2020-06-09

**Authors:** Andrea Gori, Odile B. Harrison, Ethwako Mlia, Yo Nishihara, Jia Mun Chan, Jacquline Msefula, Macpherson Mallewa, Queen Dube, Todd D. Swarthout, Angela H. Nobbs, Martin C. J. Maiden, Neil French, Robert S. Heyderman

**Affiliations:** a NIHR Mucosal Pathogens Research Unit, Division of Infection and Immunity, University College London, London, United Kingdom; b Department of Zoology, University of Oxford, The Peter Medawar Building for Pathogen Research, Oxford, United Kingdom; c Malawi-Liverpool-Wellcome Trust Clinical Research Programme, College of Medicine, University of Malawi, Blantyre, Malawi; d University of Malawi, College of Medicine, Blantyre, Malawi; e Queen Elizabeth Central Hospital, Blantyre, Malawi; f Institute of Infection and Global Health, University of Liverpool, Liverpool, United Kingdom; g Bristol Dental School, University of Bristol, Bristol, United Kingdom; University of Toronto

**Keywords:** *Streptococcus agalactiae*, pan-genome, GWAS, population structure, bacterial phylogeny, virulence, bacterial genomics

## Abstract

Streptococcus agalactiae (group B streptococcus; GBS) is a colonizer of the gastrointestinal and urogenital tracts, and an opportunistic pathogen of infants and adults. The worldwide population of GBS is characterized by clonal complexes (CCs) with different invasive potentials. CC17, for example, is a hypervirulent lineage commonly associated with neonatal sepsis and meningitis, while CC1 is less invasive in neonates and more commonly causes invasive disease in adults with comorbidities. The genetic basis of GBS virulence and the extent to which different CCs have adapted to different host environments remain uncertain. We have therefore applied a pan-genome-wide association study (GWAS) approach to 1,988 GBS strains isolated from different hosts and countries. Our analysis identified 279 CC-specific genes associated with virulence, disease, metabolism, and regulation of cellular mechanisms that may explain the differential virulence potential of particular CCs. In CC17 and CC23, for example, we have identified genes encoding pilus, quorum-sensing proteins, and proteins for the uptake of ions and micronutrients which are absent in less invasive lineages. Moreover, in CC17, carriage and disease strains were distinguished by the allelic variants of 21 of these CC-specific genes. Together our data highlight the lineage-specific basis of GBS niche adaptation and virulence.

The genome sequences of almost 2,000 GBS samples isolated from both animals and humans in high- and low- income countries were analyzed using a pan-genome-wide association study approach. This allowed us to identify 279 genes which are associated with different lineages of GBS, characterized by a different virulence and preferred host. Additionally, we propose that the GBS now carried in humans may have first evolved in animals before expanding clonally once adapted to the human host.

These findings are essential to help understand what is causing GBS disease and how the bacteria have evolved and are transmitted.

## Abstract (precis)

GBS is a leading cause of mortality in newborn babies in high- and low-income countries worldwide. Different strains of GBS are characterized by different degrees of virulence, where some are harmlessly carried by humans or animals and others are much more likely to cause disease. The genome sequences of almost 2,000 GBS samples isolated from both animals and humans in high- and low- income countries were analyzed using a pan-genome-wide association study approach. This allowed us to identify 279 genes which are associated with different lineages of GBS, characterized by a different virulence and preferred host. Additionally, we propose that the GBS now carried in humans may have first evolved in animals before expanding clonally once adapted to the human host. These findings are essential to help understand what is causing GBS disease and how the bacteria have evolved and are transmitted.

## INTRODUCTION

Streptococcus agalactiae (group B streptococcus; GBS) forms part of the normal gastrointestinal and urogenital microbiota, occasionally causing life-threatening invasive disease in infants, pregnant women, and adults with comorbidities (1). Since the 1970s, GBS has been reported as a leading cause of neonatal mortality and morbidity in the United States (2), but it is increasingly recognized that the burden is greatest in low-to-middle income countries. In sub-Saharan Africa, where up to 30% of women carry GBS asymptomatically, the incidence of invasive GBS disease in neonates has been reported to be up to 2.1 per 1,000 live births, with case fatality rates between 13 and 46% (3–5).

In neonates, early onset disease (EOD) in the first week of life typically presents as pneumonia or sepsis (5, 6). Late-onset disease (LOD) develops from 7 days to 3 months after birth and is frequently characterized by meningitis, leading to chronic neurological damage, seizures, blindness, and cognitive impairment in those that survive (5, 7). The gastrointestinal tract is the reservoir for GBS and the most likely source for maternal vaginal colonization (8). This may lead to GBS transmission before or during birth, potentially leading to early onset disease in the infant (5). The route for late-onset colonization and disease is less clear; while vertical transmission is still possible, environmental transmission and acquisition are considered more common (9).

GBS capsular polysaccharide is a key virulence factor (10) and the basis for serotyping. Ten GBS capsular serotypes have been described (11). Serotypes Ia, Ib, II, III, and V account for 98% of human carriage serotypes isolated globally, although prevalence of each serotype varies by region (12). Serotype III accounts for 25% to 30% of strains isolated in Europe and Africa but only 11% of strains isolated in North America or Asia. Serotypes VI, VII, VIII, and IX are frequently isolated in southern, southeastern, and eastern Asia, but are relatively rare elsewhere (12). Multilocus sequence typing (MLST) has identified 6 major clonal complexes (CCs) in humans: CC1, CC10, CC17, CC19, CC23, and CC26 (13, 14). In recent years it has become apparent that some CCs have a greater potential to cause invasive disease, while others are largely associated with asymptomatic carriage. CC1, CC23, and CC19, for example, are the predominant colonizers of pregnant women, well adapted to vaginal mucosa with a limited invasive potential in neonates (15, 16). In contrast, CC17 strains, mostly serotype III, are associated with neonatal sepsis and meningitis, accounting for more than 80% of LOD (1, 17). Comparative phylogeny of human and bovine GBS strains suggested that CC17 emerged recently from a bovine ancestor (CC67) and is characterized by limited recombination (18). However, this has been challenged (1), and the relationship between isolates from these different hosts remains uncertain.

Colonization and persistence of GBS in different host niches depends upon the ability to adhere to the mucosal epithelium (1, 19, 20) utilizing numerous bacterial adhesins, including fibrinogen binding protein (Fbs), C5a peptidase (ScpB), and GBS immunogenic bacterial adhesin (BibA) (21–23). Biofilm formation is essential to promoting colonization, which is also enhanced by bacterial capsule and type IIa pili (24, 25). Biofilm formation also plays a central role in the phenotype switch from commensal to pathogen (26). Deletion of the gene encoding biofilm regulatory protein A (BrpA) was shown to impair both biofilm formation and the ability of the bacterium to colonize and invade the murine host (26). The expression of these virulence factors varies by CC, with the Fbs proteins carried by the hypervirulent lineage CC17, for instance, characterized by specific deletions and frameshift mutations that alter the sequence or expression rate (27). S. agalactiae is able to survive both the acidic vaginal environment and within the blood (23). Transcription analyses have suggested that this transition is largely mediated by the two-component regulator CovRS (28, 29). Recently, specific gene substitutions in the two-component signal transducer (TCS) CovRS have been identified in disease-adapted CC17 GBS clones (30). How widespread these genetic adaptations are among CC17 clones and whether different adaptations confer enhanced colonization and disease potential in other CCs is uncertain.

Here, we report a pan-genome-wide association study of genome sequence data from 1,988 GBS carriage- or invasive disease-associated isolates from different hosts and countries. This study revealed that GBS CCs possess distinct collections of genes conferring increased potential for persistence, including genes associated with carbohydrate metabolism, nutrient acquisition, and quorum sensing. Within CC17, allelic variants of these crucial genes distinguish carriage from invasive strains. The differences in the GBS CCs analyzed are not geographically restricted, and we postulate that they may have emerged from an original ancestral GBS strain in animal hosts before crossing to humans.

## RESULTS

### GBS whole-genome sequencing data set.

A total of 358 Malawian GBS genome sequences were initially available. Of these, 55 samples did not pass the quality checks, therefore the final Malawian data set was composed of 303 GBS strains (Table 1), including 131 isolated from invasive disease in children and 166 isolated from healthy mothers, in which draft genome assemblies had an average *N*_50_ of 163,462 bp (range 12,593 to 717,849 bp), average contig number of 70 (range 20 to 377), and average longest contig of 30,148 bp (range 44,691 to 1,019,176 bp).

**TABLE 1:** Characteristics of GBS isolates

Isolate type	Country	Source	Count	No. invasive	No. missing data
Human	Malawi	This work^*a*^	303	131	6
	Kenya	Seale et al. (63)	1034	71	0
	USA	Flores et al. (64)	99	99	0
	Canada	Teatero et al. (31)	141	141	0
	The Netherlands	PRJEB14124^*b*^	300	unknown	300
	Unknown	***^c^	24	unknown	24
Animal^*d*^	Italy	***	3		
	Kenya	***	2		
	Germany	***	1		
	Brazil	***	1		
	Unknown	***	80		

a Isolated from Queen Elizabeth Central Hospital, Blantyre.

b Jamrozy et al. (65).

c ***, Genomes retrieved from https://pubmlst.org/. Full metadata are reported in Table S1.

d Animal isolates are reported to be isolated from cattle (*n* = 49), fish (*n* = 23), frogs (*n* = 3), or other animal sources (*n* = 12).

10.1128/mBio.00728-20.7 TABLE S1 Metadata of each GBS isolate described in this work. From left to right each column shows the isolate name, the clonal complex to which the isolate belongs (CC1, CC6, CC10, CC19, CC17, CC23, single ST, or N/D), the source of isolation (animal or human, in which case it is reported as carrier, invasive, or unknown), the country of isolation, the serotype, the year of isolation (where available), the MLST-type, and the accession number of each isolate (where available). Download Table S1, PDF file, 0.3 MB. Copyright © 2020 Gori et al. 2020 Gori et al. This content is distributed under the terms of the Creative Commons Attribution 4.0 International license.

A further 1,598 clinical isolates were included: these comprised 1,034 Kenyan, 99 American, 141 Canadian, and 300 Dutch strains randomly selected from 1,512 isolates from the Netherlands (Table 1). Animal isolates (87) sampled in several different countries were also included (Table 1). Where information was available, 446 (22.4% of the 1,988 total) strains were associated with invasive disease (bacteremia or meningitis) and 1,125 (56.6%) from healthy carriers. Meta-data consisting of country of origin, year of isolation, capsular serotype, MLST, and accession number are reported in Table S1 in the supplemental material. The genome sequences from 1,988 isolates were used for the analysis.

### Clonal complex assignment and core genome phylogeny.

Six CCs were identified: CC1, CC6, CC10, CC19, CC17, and CC23 according to the BURST algorithm (31) and core genome phylogeny (Table S1 and S2). Several sequence types (STs) were found in just one country (e.g., ST 866 was found exclusively in Malawi or ST 196 was found only in Kenya); however, these STs were always represented by less than 20 isolates. With the exception of the United States, where isolates were intentionally selected to represent only CC1 (31), and the rare CC6 represented by 22 isolates, CCs were distributed across all of the countries analyzed.

10.1128/mBio.00728-20.8 TABLE S2 Sequence type defining each clonal complex and number of STs isolated per country. The table is divided into 8 horizontal sectors (CC1, CC6, CC10, CC19, CC17, CC23, single ST, or N/D). In each sector columns show (from left to right) which ST is represented in each CC; number of isolates belonging to a particular ST are found in each of the countries where the isolates described in this study were sourced (Brazil, Canada, Germany, Italy, Kenya, Malawi, Netherlands, USA, or unknown source). Download Table S2, PDF file, 0.04 MB. Copyright © 2020 Gori et al. 2020 Gori et al. This content is distributed under the terms of the Creative Commons Attribution 4.0 International license.

**FIG 1:**
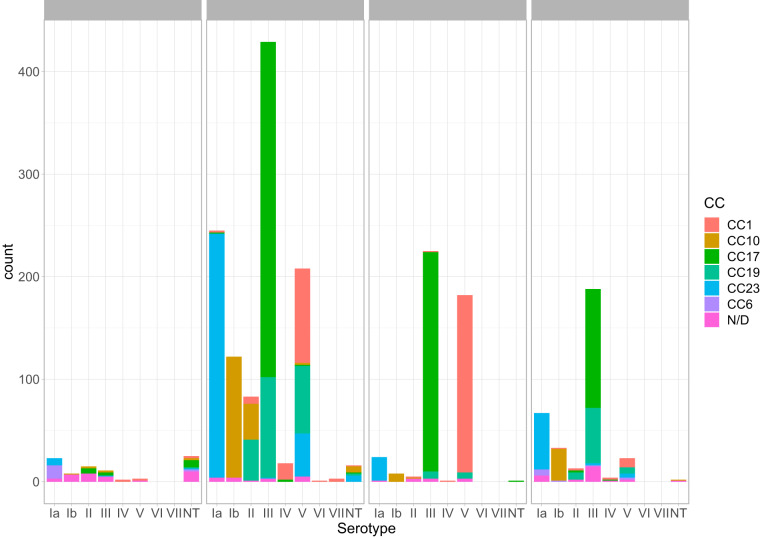
Isolates used in this study, stratified per serotype, CC, and source. Clinical isolates are grouped as invasive (including strains isolated from children and adults affected by any GBS invasive disease), carrier (including healthy carrying mothers), and unknown, where metadata were not available.

**FIG 2:**
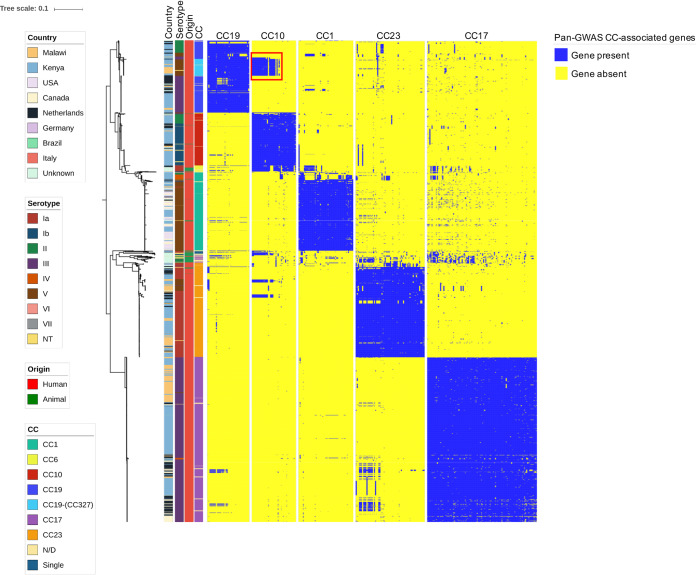
Core genome-based population structure of GBS. The phylogenetic tree is annotated with 4 colored strips representing the clonal complex, the country of isolation, the origin, and the serotype of each strain. The three binary heatmaps represent the presence (blue) or absence (yellow) of the genes identified by the pan-GWAS pipeline. The tree is rooted at midpoint. The reference strain used in this analysis was COH1, reference HG939456. The red square in the CC10 heatmap highlights the cluster of CC10-associated genes found in CC19 clones. Trees built with different reference strains are shown in Fig. S1 in the supplemental material and show analogous topology.

While each serotype in the clinically derived WGS was predominantly associated with only one or two CCs, the animal isolates were more variable (Fig. 1). SNPs identified in the part of the genome shared by all isolates (∼26,000 polymorphisms) were used to infer the ML phylogenetic trees (Fig. 2, Fig. S1). Similar results were obtained calculating recombination-censored maximum-likelihood core-genome phylogeny on five different 140-strain subsets using ClonalFrame-ML, as shown in Fig. S2. CCs clustered in distinct branches of the tree; in particular, CC17 and CC23 produced two clusters. A group of 41 animal-derived WGS data clustered within a separate clade (Fig. 2), branching from human-associated CC17, hence suggesting a human origin for these animal strains. However, 46/87 isolates were located in clades associated with human-derived samples and CCs: three animal isolates for instance (MRI Z1 201, MRI Z1 200, and MRI ZI 203) clustered within the clinical isolates in the CC23 clade. We also observed several cases of CC17 animal isolates (classified as CC17 with eBurst which indicates a close relatedness to the CC17 clade but clustering within these animal strains, such as FSL S3 603 and LDS 623). This pattern could be interpreted as zoonotic transfer, with the human-associated CCs arising in animals before undergoing clonal expansion after infection of the human host. However, given the fragmented sampling frame of this data set, as well as the lack of rooting in this phylogenetic analysis (Fig. 2), we can only speculate on this matter.

10.1128/mBio.00728-20.1 FIG S1 Core genome-based population structure of GBS built with alternative reference strains. Trees showing the GBS population structure as in Fig. 1, produced with different reference strains. Reference strains used for the four trees are: NC_021485, strain 09mas018883; NC_007432, strain A909; NC_018646, strain GD201008; NC_004368, strain NEM316. For each tree, the annotation is analogous to the one described for Fig. 1. Download FIG S1, PDF file, 0.1 MB. Copyright © 2020 Gori et al. 2020 Gori et al. This content is distributed under the terms of the Creative Commons Attribution 4.0 International license.

10.1128/mBio.00728-20.2 FIG S2 Recombination-censored maximum-likelihood phylogenetic trees on 5 random 140-strain subsets of the GBS genome dataset used in this work. Twenty isolates were selected randomly from each CC. The annotation of each tree is reported in the legend and is analogous to Fig. 1 for CC, origin, serotype, and country of isolation. Trees are unrooted. Download FIG S2, PDF file, 0.2 MB. Copyright © 2020 Gori et al. 2020 Gori et al. This content is distributed under the terms of the Creative Commons Attribution 4.0 International license.

### Pangenome and pan-GWAS.

Scoary has previously been used for a similar pan-GWAS analysis of three CC17 strains (30). In this study, we applied it on a data set of 1,988 strains, representing 6 different clonal complexes (Fig. 1): 1,374 genes were included in the core genome (i.e., present in more than 95% of the strains, “core” and “soft-core” genes), and 12,457 genes in the accessory genome (i.e., less than 95% of the strains, “shell” and “cloud” genes) (32). We observed that pangenome saturation was achieved. A total of 51, 41, 39, 102, and 64 genes associated, respectively, with CC1, CC10, CC19, CC17, and CC23 were identified (Table 2 and Table S3), with a specificity and sensitivity in defining the CC given the annotated CDS (and vice versa) greater than 90% (*P* < 0.05). The pipeline was not applied to CC6, which was represented by only 22 genomes in our data set. BLASTn was used to confirm whether gene sequences associated with each CC in the pan-GWAS were completely absent in different CCs, or had accumulated sufficient mutations to fail recognition by automated annotation (i.e., Prokka). We identified 57 such genes in CC17 out of the 102 identified by the Scoary pipeline, 22 genes in CC23, 4 genes in CC1, 9 genes in CC10, and 5 in CC19 (Fig. S3 and Table S3). This suggests that the genes characterizing a particular CC may have been rendered nonfunctional (i.e., pseudogenes) in other CCs. Table S3 highlights which CC-associated genes are completely absent and which genes are characterized by mutations (SNPs or indels) that alter the protein sequence with point mutations or truncation. Table 2 summarizes the number of genes driving the pan-GWAS analysis and the nature of the mutations encountered.

**TABLE 2:** Summary of the number of genes driving the pan-GWAS in each clonal complex and type of mutations encountered

Clonal complex	Presence/absence	Nonsynonymous SNPs	Synonymous SNPs	Nonsense SNPs	Total	GWAS-genes island^*a*^
CC1	47	3	0	1	51	0
CC10	32	3	2	4	41	0
CC17	38	42	10	12	102	0
CC19	34	5	0	0	39	1
CC23	42	14	3	5	64	0

a A GWAS-gene island is defined as a region of the genome of at least 200kbp where > 90% of the coding regions are GWAS-driving genes.

10.1128/mBio.00728-20.3 FIG S3 Heatmaps based on the BLASTn score of each CC-characterizing gene for each isolate. Heatmaps were produced with pheatmap package in R (clustering of rows and columns was performed using the Euclidean method). Each heatmap shows 1,988 isolates on the rows and the CC-associated genes on the column. Each row-clustering tree (related to the isolates) is annotated with colored strips representing the clonal complex. Cells are colored according to the normalized BLAST bitscore score (red = 0, green = 1), as described in the Materials and Methods. Strains belonging to CC10-ST327 and CC10-ST328 are reported as CC327 in this representation. Download FIG S3, PDF file, 0.4 MB. Copyright © 2020 Gori et al. 2020 Gori et al. This content is distributed under the terms of the Creative Commons Attribution 4.0 International license.

10.1128/mBio.00728-20.9 TABLE S3 Genes defining each CC as identified by pan-GWAS. The table is divided into five sections, relative to CC1, CC10, CC19, CC17, and CC23. Each column shows (from left to right) the name of the gene identified by pan-GWAS, the length of the putative protein produced by each gene, the KEGG database id (where available), a short annotation of each gene (according to KEGG and/or Prokka where available, otherwise reported as hypothetical protein), the functional class to which each gene belongs (metabolic, reported as “met”; environmental information processing, “env”; or cellular processes, “cell”; according to the KEGG annotation, the type of variation between different clonal complexes (“point mutations”, “synonymous point mutations”, “truncated protein”, or “gene absent”). Where neither Prokka nor KEGG annotation reported a gene name, an arbitrary gene name was assigned to the hypothetical gene following the scheme, “*g*”, followed by the clonal complex and an incremental number. Download Table S3, PDF file, 0.1 MB. Copyright © 2020 Gori et al. 2020 Gori et al. This content is distributed under the terms of the Creative Commons Attribution 4.0 International license.

10.1128/mBio.00728-20.10 TEXT 1 Representative sequences of CC-specific genes. Download Text S1, TXT file, 0.2 MB. Copyright © 2020 Gori et al. 2020 Gori et al. This content is distributed under the terms of the Creative Commons Attribution 4.0 International license.

**FIG 3:**
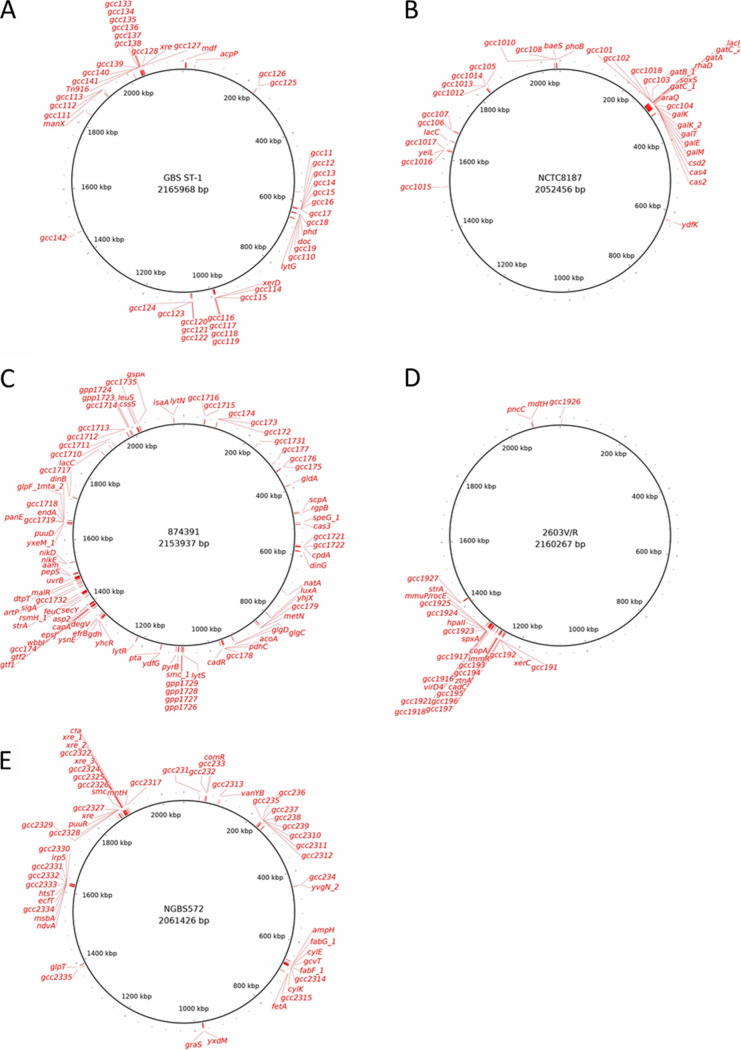
Location of genes identified by the pan-GWAS pipeline on a strain belonging to CC1 (A), CC10 (B), CC17 (C), CC19 (D), and CC23 (E). Gene location on each chromosome is represented by a red mark. Gene names correspond to the ones reported in Table S3.

Gene location identified from the pan-GWAS analyses in CC1, CC10, CC17, and CC23 was evenly spread across the chromosome and not clustered in particular areas, consistent with the observed gene associations not resulting from a chromosomally integrated plasmid or transposon pathogenicity island acquired through horizontal gene transfer (Fig. 3). One exception was CC19, where the majority of the 39 genes were clustered in a 200-kbp region of the chromosome. Gene synteny was conserved across different isolates; in Fig. S4 the parallel vertical lines show how the genes conserved among the CC17 isolates are present in the same relative location (represented as different colored blocks in the mauve representation) in three representative genomes.

10.1128/mBio.00728-20.4 FIG S4 Synteny of CC17-characterizing genes. The image shows the alignment of three CC17 S. agalactiae genomes (strains 874391, BM110, and SGM6) and 104 CC17-associated genes (at the bottom). Each vertical line represents a sequence found in the same location in all the analyzed sequences. Image obtained with the software Mauve. Download FIG S4, JPG file, 0.2 MB. Copyright © 2020 Gori et al. 2020 Gori et al. This content is distributed under the terms of the Creative Commons Attribution 4.0 International license.

The majority of the pan-GWAS-identified genes were associated with only one CC, but a particular cluster of genes associated with CC10 (including the *gatKTEM* system for galactose metabolism) was also present in a set of isolates belonging to CC19 (Fig. 2). These isolates were all from Africa (Malawi and Kenya) and were ST-327 and ST-328.

### Functional pathways affected by CC-specific genes.

A total of 279 genes were found to be CC-specific (Table S3). Genes characteristic of CC17 and CC23 were classified into five functional categories (Table 3): metabolism, environmental information processing, cellular processes, human disease, and genetic information processing. In both CCs, the most-represented functional families were in these categories, including metabolic genes and environmental information processes.

**TABLE 3:** Pathways and functional categories identified by KEGG annotation in the five groups of CC-associated genes^*a*^

Clonal complex	Kegg no.	Pathway
CC1		
Metabolism (09100)	01130	Biosynthesis of antibiotics
	00052	Galactose metabolism
	00999	Biosynthesis of secondary metabolites—unclassified
Environmental Information Processing (09130)	02060	Phosphotransferase system (PTS)
CC10		
Metabolism (09100)	01100	Metabolic pathways
	01110	Biosynthesis of secondary metabolites
	01120	Microbial metabolism in diverse environments
	01130	Biosynthesis of antibiotics
	00010	Glycolysis/gluconeogenesis
	00040	Pentose and glucuronate interconversions
	00051	Fructose and mannose metabolism
	00052	Galactose metabolism
	00561	Glycerolipid metabolism
	00600	Sphingolipid metabolism
	00603	Glycosphingolipid biosynthesis—globo and isoglobo series
Environmental Information Processing (09130)	02060	Phosphotransferase system (PTS)
CC19		
Metabolism (09100)	01100	Metabolic pathways
	00270	Cysteine and methionine metabolism
	00760	Nicotinate and nicotinamide metabolism
Environmental Information Processing (09130)	03070	Bacterial secretion system
CC17		
Metabolism (09100)	00010	Glycolysis/gluconeogenesis
	00020	Citrate cycle (TCA cycle)
	00052	Galactose metabolism
	00500	Starch and sucrose metabolism
	00520	Amino sugar and nucleotide sugar metabolism
	00620	Pyruvate metabolism
	00630	Glyoxylate and dicarboxylate metabolism
	00640	Propanoate metabolism
	00680	Methane metabolism
	00910	Nitrogen metabolism
	00561	Glycerolipid metabolism
	00230	Purine metabolism
	00240	Pyrimidine metabolism
	00250	Alanine, aspartate and glutamate metabolism
	00260	Glycine, serine, and threonine metabolism
	00280	Valine, leucine, and isoleucine degradation
	00220	Arginine biosynthesis
	01007	Amino acid related enzymes
	00430	Taurine and hypotaurine metabolism
	01003	Glycosyltransferases
	01005	Lipopolysaccharide biosynthesis proteins
	01011	Peptidoglycan biosynthesis and degradation proteins
	00760	Nicotinate and nicotinamide metabolism
	00770	Pantothenate and CoA biosynthesis
	01001	Protein kinases
	01002	Peptidases
	03021	Transcription machinery
	03016	Transfer RNA biogenesis
CC17 (continued)		
	00970	Aminoacyl-tRNA biosynthesis
	03110	Chaperones and folding catalysts
	03060	Protein export
	03420	Nucleotide excision repair
	03400	DNA repair and recombination proteins
Environmental Information Processing (09130)	02000	Transporters
	02010	ABC transporters
	02060	Phosphotransferase system (PTS)
	03070	Bacterial secretion system
	02020	Two-component system
	02044	Secretion system
	02022	Two-component system
Cellular Processes (09140)	04147	Exosome
	02048	Prokaryotic defense system
	02024	Quorum sensing
	02026	Biofilm formation-*Escherichia coli*
Unclassified (09190)	99982	Energy metabolism
	99984	Nucleotide metabolism
	99999	Others
	99977	Transport
CC23		
Metabolism (09100)	00630	Glyoxylate and dicarboxylate metabolism
	00061	Fatty acid biosynthesis
	01040	Biosynthesis of unsaturated fatty acids
	01004	Lipid biosynthesis proteins
	00260	Glycine, serine, and threonine metabolism
	00550	Peptidoglycan biosynthesis
	01011	Peptidoglycan biosynthesis and degradation proteins
	00780	Biotin metabolism
	00670	One carbon pool by folate
	01008	Polyketide biosynthesis proteins
	01053	Biosynthesis of siderophore group nonribosomal peptides
	00333	Prodigiosin biosyntheses
	01002	Peptidases
Cellular Processes (09140)	02000	Transporters
	02010	ABC transporters
	02020	Two-component system
	02042	Bacterial toxins
Human Disease (09100)	02048	Prokaryotic defense system
	02024	Quorum sensing
	01502	Vancomycin resistance
	01504	Antimicrobial resistance genes
Unclassified (09190)	99988	Biosynthesis and biodegradation of secondary metabolites
Genetic Information Processing (09120)	03000	Transcription factors

a For each clonal complex the functional categories of the GWAS-associated genes are shown. The metabolic pathways affected by those genes and their Kegg reference numbers are reported.

Differences in metabolic pathways between CC17 and CC23 included carbohydrate, amino acid, nitrogen compound, and fatty acid metabolism. Siderophores for the uptake and transport of micronutrients (i.e., iron or nickel), and essential for successful colonization of the human host in several bacterial pathogens (33–35), also exhibited significant variation, for instance with genes for nickel uptake (*nikE* and *nikD*) and iron transport (*feuC*) truncated or characterized by SNPs in non-CC17 strains (Table S3).

CC17 and CC23 also showed differences in the genes affecting the environmental information processing functional pathways characterized by the presence of phosphotransferase (PTS) systems and two-component systems (TCS), used for signal transduction and sensing of environmental stimuli. Moreover, in the same functional category, differences were present in secretion systems, transporters, quorum sensing, and bacterial toxins. These pathways are used by GBS not only in colonization of the host, but also to gain competitive advantage against other microorganisms occupying a particular ecological niche (36).

Genes for prokaryotic defense systems, such as the CRISPR-Cas9 system, were also found, as well as proteins involved in genetic information processing such as transcription factors and regulators that may affect the expression of multiple genes (37). Finally, antibiotic resistance also appears among the lineage-specific characteristics; in particular, CC23 is the only CC showing typical genes involved in vancomycin resistance. CC17 also showed the presence of genes belonging to the KEGG group for “nucleotide excision repair” and “DNA repair/recombination protein” (KO numbers 03420/03400; Table 3), which could indicate a variation in mutagenesis rate and thus capacity to respond to changes in environmental conditions and presence of stresses.

In contrast, the genes defining CC1, CC10, and CC19 were confined to metabolism, environmental information processing, and genetic information processing. Genes involved with regulation and environmental sensing (PTS systems), as well as secretion systems, were identified in this group of CCs. In particular, a gene encoding the VirD4 type IV secretion system protein was associated with CC19. CC10 was characterized by an array of genes involved in carbohydrate metabolism and uptake, such as the ABC transport system for multiple sugar transport.

The majority of genes characteristic for CC1 were of unknown function, with the exception of genes involved with genetic regulation and a complete toxin/antitoxin system *phd/doc* (38). These systems are often described as a tool for stabilizing extrachromosomal DNA (i.e., plasmids), but they are often found integrated chromosomally in both Gram-positive and Gram-negative bacterial species—though their function in this setting is unclear (39).

In relation to the CC17-associated genes, we also checked for allelic variants specific to strains isolated from invasive disease or carriage. Figure S4 shows the proportion of CC17 invasive or carriage strains, and the frequency of each allelic variant. We identified 21 genes with alleles that statistically differentiated strains isolated from carriage and invasive disease (Fisher test, *P* < 0.05; Table 4). The DNA sequence of the allelic variant differed by a single polymorphism in all cases. In 15/21 cases this nucleotide change was translated into an amino acid change (missense), while in a single case the mutation resulted in a truncated protein (nonsense). Figure S6 shows these mutations in relation to the population structure of CC17. The tree shows how these mutations are associated with particular clades, rather than the disease phenotype. This suggests that the mutations have been acquired historically by the CC17 bacterial population, and have become fixed in each clade. Nonetheless, each CC17 cluster is not equally represented by disease and carriage strains, suggesting that these mutations may have contributed to the development of an invasive phenotype. These genes have the potential to affect the metabolism and virulence of the bacterial strains. For example, although the major pilin synthesis gene is known to be characterized by locus variants which are associated with biofilm and virulence (namely, variants PI-I, PI-IIa and PI-IIb) (40), CC17 is characterized by the presence of PI-I/PI-IIb. Smaller variations within the locus PI-IIb appear to be associated with CC17 isolated from carriage, suggesting that this gene may be impaired in functionality. Similarly, the *prtP* gene and the *efrB* genes, encoding, respectively, for a virulence-associated protease and for the ATP-binding cassette of a multidrug-efflux pump, have alleles that are more common in strains isolated from disease, highlighting the potential for these allelic variations to result in a more virulent phenotype (41).

**TABLE 4:** CC17-associated genes showing at least one allele statistically associated with strains isolated from either invasive disease or from carriage

Gene	Allele 1		Allele 2		No. mismatches (aa)	% difference (aa)
	*P* value^*a*^	Odds ratio	*P* value	Odds ratio		
*gdh*	8.98E−18	84.47	1.67E−03	0.19	0	0
*dinG*	5.48E−10	0.08	0.063	0.55	0	0
*gcc178*	1.19E−19	0.09	0.151	1.41	0	0
*metN*	6.27E−18	0.10	2.97E−14	0.10	1	0.4
*yhjX*	0.028	0.24			1	0.2
*pta*	0.018	0.19			1	0.3
*strA*	0.006	5.86			0	0
*gcc1730*	5.72E−03	5.93			1^*b*^	0.3^*b*^
*gpp1725*	1.11E−04	0.47			1	0.6
*endA*	7.42E−05	0.46			1	0.9
*dtpT*	2.41E−05	0.43			1	0.2
*cadR*	1.48E−05	0.42			1	0.3
*pyrB*	3.80E−08	0.10			1	0.3
*gcc171*	3.27E−09	0.15			1	1.1
*gcc176*	5.64E−10	3.56			1	0.2
*gcc1713*	5.48E−10	3.55			0	0
*natA*	1.04E−10	3.69			1	0.9
*inlA_2*	2.42E−15	0.09			1	0.1
*prtP_2*	1.71E−16	42.87			1	0.1
*glgD*	1.78E−17	83.77			1	0.9
*efrB*	1.78E−18	49.48			1	0.2

a *P* values are Benjamini-Hochberg corrected.

b *gcc1730* showed only one mismatch in the protein alignment, which introduced a stop codon in position 122.

10.1128/mBio.00728-20.5 FIG S5 Genes showing alleles statistically associated with carriage or invasive disease in CC17 strains. Each bar plot shows the frequency of each allele in each of the 21 CC17-associated genes observed to have at least one allele associated with disease or carriage. Different numbers on the *x* axis represent different allelic configuration of the gene. *P* value < 0.05, *, not significant. Download FIG S5, PDF file, 0.3 MB. Copyright © 2020 Gori et al. 2020 Gori et al. This content is distributed under the terms of the Creative Commons Attribution 4.0 International license.

10.1128/mBio.00728-20.6 FIG S6 Population structure of CC17 isolates and presence of alleles associated with disease or carriage. Vertical colored strips show the clinical origin of each isolate (disease or carriage) and the alleles associated with disease or carriage for the genes reported in Fig. S4. The genes are reported in the tree annotation in the following order: *cadR, dinG, dtpT, efrB, endA, gcc171, gcc 176, gcc178, gcc1713, gcc1730, gdh, glgD, gpp1725, inlA_2, metN, natA, prtP_2, pta, pyrB, srtA, yhjX*. Download FIG S6, TIF file, 0.4 MB. Copyright © 2020 Gori et al. 2020 Gori et al. This content is distributed under the terms of the Creative Commons Attribution 4.0 International license.

In order to test the impact of the mutations targeting the *efrB* multidrug-efflux pump, we assessed susceptibility (MIC) to a range of unrelated compounds previously shown to be informative in S. pneumoniae (42) (Table 5). For three out of five molecules (erythromycin, chloramphenicol, and norfloxacin), the CC17 strain we tested had the highest MIC, although this was also seen in other non-CC17 strains. The acriflavin MIC did not vary between the different strains. The berberine MICs for CC17 and CC1 were lower than for CC23 and CC19.

**TABLE 5:** Susceptibility of four S. agalactiae strains against five antimicrobial agents

Antimicrobial	MIC (μg/ml)^*a*^			
	COH1 (serotype III, cc17)	NEM316 (serotype III, cc23)	2603V/R (serotype III, cc19)	H36B (serotype Ib, cc1)
Erythromycin	0.25	0.125	0.25	0.25
Chloramphenicol	4	4	4	2
Norfloxacin	>8^*b*^	>8^*b*^	4	4
Acriflavine	8	8	8	8
Berberine	32	64	64	32

a MICs were determined from 3 experiments.

b Value of 8 μg/ml was the higher detection limit for norfloxacin due to acidification and precipitation of media components.

## DISCUSSION

S. agalactiae isolated from human and animal sources is characterized by a range of clonal complexes and sequence types. Each CC appears to be phenotypically different, with CC1 being commonly isolated in adult disease, and CC17 (associated with capsular serotype III) commonly isolated in neonatal disease and demonstrating hypervirulence (1, 16). We show that these different CCs are characterized by different gene sets belonging to functional families involved in niche adaptation and virulence. This is in part reflected in the various potential of different CCs to cause invasive diseases in different human hosts, as illustrated by the hypervirulence of CC17 in neonates, the lower neonatal invasive potential of CC1, CC19, and CC23 clones, and the propensity of CC1 to cause disease in adults with comorbidities (15, 16). Importantly, these CC-specific genetic characteristics and the pattern of gene presence and absence are independent of geographical origin, with the exception of the CC10 gene cluster present in the strains isolated from Africa belonging to ST327 and ST328. Furthermore, within CC17 we have identified several, functionally important allelic variants associated with either carriage or disease.

Among the hypervirulent CC17-specific genes, there were several examples of previously identified genes associated with human disease due to GBS and other related bacteria. For instance, the transporter Nik that controls the uptake of nickel is essential for survival in the human host. A homolog of Nik has been shown to be essential for Staphylococcus aureus in the causation of UTIs (43). The *dldh* gene, encoding the dihydrolipoamide dehydrogenase enzyme (44), has been implicated in several virulence-related processes in Streptococcus pneumoniae, such as survival within the host and production of capsular polysaccharide. Mutants lacking the *dldh* gene are unable to cause sepsis and pneumonia in mouse models (44). Surface proteases in S. agalactiae are described to have several virulence-associated functions, such as inactivation of chemokines that recruit immune cells at the site of infection or facilitate invasion of damaged tissue (45, 46). We have identified PrtP and ScpA proteases, both characterized by the presence of C5a peptidase domains and a signal peptidase SpsB, specific to this complex. Genes known to be associated with CC17 hypervirulence have also been identified in this analysis, including the Pi-IIb locus (40), part of which is represented by the CC17-associated genes *gcc1732*, *lepB*, *inlA_2*, and *gcc1733* (Table S3), supporting the validity of this analysis. Allelic variation of virulence-associated genes has previously been used to identify genes classifying invasive and noninvasive strains in other streptococcal species (41). A proportion of CC17-specific genes also showed unique alleles associated with invasive disease or carriage strains. Sixteen of twenty-one allelic variants resulted in a difference that was translated into the protein sequence, including regulatory proteins and virulence- or metabolism-associated proteins, such as ABC transport systems, a major pilin protein, and a C5a peptidase. These data suggest there have been further selection processes within hypervirulent CC17 that could result in strains characterized by different virulence levels.

A phenotypic analysis was carried out to identify the potential effect of mutations in the *efrB* multidrug efflux pump that differentiated CC17 from the other clonal complexes. Using the MIC for a panel of antimicrobial molecules, we have not conclusively been able to show that mutations in *efrB* gene alone change the MIC. This is likely to be confounded by other differences in the non-CC17 genomes. CC23, for instance, encodes several ABC transporters in its accessory genome (such as such as the *wxdM* gene; Table S3) and we cannot exclude that some of these would be involved with efflux of deleterious molecules.

Further work is needed to assess the effect of the GWAS-driving mutations in an isogenic CC17 background.

CC23-specific genes identified are putatively involved in virulence and host invasion, including *mntH*, a gene encoding a manganese transport protein. During a bacterial infection the host limits access to manganese, among other micronutrients, and it has been shown that S. aureus responds to this host-induced starvation by expressing metal transporters, such as MntH (35). Interestingly, CC23 is also associated with *vanY*, a gene implicated in vancomycin resistance in other streptococci (47). GBS is typically susceptible to vancomycin (48), an antibacterial glycopeptide obtained from Streptomyces orientalis, which inhibits cell wall synthesis, alters the permeability of the cell membrane, and selectively inhibits RNA synthesis (49). Whether the presence of this gene also facilitates niche adaptation in the context of complex host-microbiota environment remains to be determined.

Lineage CC10, and the sublineage CC19 that includes the strains belonging to ST327 and ST328, are mostly characterized by metabolic genes, consistent with the lower virulence of these clonal complexes. The genes *galTKEM* are present in these two lineages only, and encode the “Leloir pathway” in other streptococci, such as S. mutans, S. thermophilus, and S. pneumoniae (50–52). This pathway in S. pneumoniae is finely tuned by CbpA and activated in tandem with the tagatose-6-phosphate pathway in order to maximize growth (53). The functionality of this pathway is yet to be described in GBS, but we hypothesize that accessing different methods to metabolize carbohydrates facilitates nutrient competition and survival. Among the nonmetabolic genes that are associated with CC19, we identified a *virD4* gene, which is part of a previously identified type IV secretion system (T4SS) (54) present in numerous bacterial species and associated with virulence effector translocation and conjugation (55, 56).

GBS is widely thought to be a zoonosis (57–61). Based on the nature and the distribution of CC-characterizing genes, and their location in the GBS genome, we hypothesize that S. agalactiae lineages that colonize humans may have initially evolved in animals and then subsequently expanded clonally in humans. Our analysis is limited by the availability of data sets from across the world and from putative animal hosts, and therefore this hypothesis needs further confirmation. However, in line with the observation that S. agalactiae has undergone genome reduction (62), we postulate that the human-adapted clones evolved in animals through loss of function of redundant genes. Having escaped the animal niche, they were then able to evade the human immune system and establish successful colonization. Recently, the “missing link” between animal and human adaptation of GBS was described to be CC103 (57). However, we have identified animal isolates belonging to human-associated CCs (e.g., CC17 and CC23) which, in the case of the CC23 strains, cluster together with human clinical isolates in the GBS population structure.

Our analysis has a number of additional limitations. First, we were confined to the current publicly available GBS human and animal genomes retrieved from https://pubmlst.org/sagalactiae/ (a total of 3,028 isolates, including the full data set from the Netherlands), plus a further 303 genomes from Malawi. Second, the GWAS pipeline we used relies on the automated annotation of software Prokka. The use of this software required the use of Roary and Scoary to produce the pangenome and the pan-GWAS. This was extremely efficient when used to annotate the thousands of bacterial genomes in this analysis, and although the genome annotations and the pangenome were manually screened for consistency and quality (such as saturation of the core and accessory genome), it could potentially introduce artifacts. Confirming the GWAS findings with the sequence alignments allowed us to identify several genes that were characterized by nonsynonymous mutations and small indels, as well as unraveling the potential artifacts that require further investigation. Finally, as our analysis is confined to the genomic differences between the different clades, further laboratory and epidemiological analysis will be needed to fully appreciate the biological consequences of these CC-specific genes.

In conclusion, we have shown that the CCs of Streptococcus agalactiae responsible for neonatal meningitis and adult colonization are characterized by the presence of specific gene sets that are not limited to particular geographical areas. In the context of GBS control measures, such as vaccination, we speculate that as the human gastrointestinal and urogenital niches are vacated by vaccine serotypes, serotype replacement could occur as a result of new GBS strains arising from animals, including cattle and fish, which are reservoirs of GBS genetic diversity.

## MATERIALS AND METHODS

### Bacterial strains, genomes, and origin.

Publicly available genome sequences from 1,574 human isolates from Kenya, the United States, Canada, and the Netherlands, together with 87 genomes from animal isolates, were analyzed (16, 63–65) (Table 1). 24 further genomes derived from human isolates were retrieved from https://pubmlst.org/sagalactiae/ and were included. The genome assemblies were not available for the isolates from Kenya and the Netherlands. In those cases, short read sequence data were retrieved from the European Nucleotide Archive (ENA, https://www.ebi.ac.uk/ena). Raw DNA reads were trimmed of low-quality ends and cleaned of adapters using Trimmomatic software (ver. 0.32) (66). *De novo* assembly was performed with SPAdes software (ver 3.8.0) (67), using a sample of 1,400,000 reads and k-mer values of 21, 33, 55, and 77. *De novo* assemblies were checked for plausible length (between 1,900,000 and 2,200,000 bp), annotated using Prokka (ver. 1.12) (68), and checked for low-level contamination using Kraken (ver. 0.10.5) (69). In cases for which more than 5% of the contigs belonged to a species different from Streptococcus agalactiae, the genome sequence was not included in any further analysis. Resulting assemblies were deposited in the pubmlst.org/sagalactiae database, which runs the BIGSdb genomics platform (70).

In addition, 303 carriage and invasive disease strains isolated in Malawi between 2004 and 2016 in the context of carriage and invasive disease surveillance were sequenced. DNA was extracted from an overnight culture using DNAeasy blood and tissue kit (Qiagen) following the manufacturer’s guidelines, and sequenced using HiSeq4000 (paired-end library 2 × 150) platform at Oxford Genomics Centre UK. Sequences were then assembled as described above.

### MLST and serotype definition.

Serotypes were determined via DNA sequence (63). BLASTn was used to align the DNA fragments typical of each serotype to the DNA assemblies (parameters: E value 1e−10, minimum 95% identity, minimum 90% query coverage). Accession numbers for the serotype-specific sequences are AB028896.2 (from 6982 to 11695, serotype Ia); AB050723.1 (from 2264 to 6880, serotype Ib); EF990365.1 (from 1915 to 8221, serotype II); AF163833.1 (from 6592 to 11193, serotype III); AF355776.1 (from 6417 to 11656, serotype IV); AF349539.1 (from 6400 to 12547, serotype V); AF337958.1 (from 6437 to 10913, serotype VI); AY376403.1 (from 3403 to 8666, serotype VII); and AY375363.1 (from 2971 to 7340, serotype VIII). Only one fragment matched each genome under these parameters, defining each isolate’s serotype. If no match was identified, the isolate was described as nontypeable (NT).

Multilocus sequence types (MLST) STs were derived from the allelic profiles of 7 housekeeping genes (*adhP*, *pheS*, *atr*, *glnA*, *sdhA*, *glcK*, and *tkt*). This grouped strains into 91 unique STs. Strains which did not show a full set of housekeeping gene alleles or were not assigned to any previously described ST (*n* = 68) were double-checked for sequence contamination and assigned to a non-sequence-typeable (NST) group.

### Phylogeny inference.

BURST (71) was used to evaluate the relatedness between different STs, and to define CCs. Five random subsets, each containing 1,000/1,988 isolates, were analyzed using eBURST on PubMLST (70). This grouped STs sharing at least five out of seven MLST loci, and identified the central ST (i.e., the ST with the highest number of single or double locus variants), and was used to define CCs. Each of the five subsets showed the same six CCs (CC1, CC6, CC10, CC19, CC17, and CC23) plus a series of singletons (STs not belonging to any CC). CCs were defined as the set of STs associated with a particular CC in at least one eBURST result.

Core-genome phylogeny of GBS data sets was inferred using the software Parsnp (from Harvest package, ver. 1.1.2) (72), which performs a core genome SNP typing and uses Fastree2 (73) to reconstruct whole-genome maximum-likelihood phylogeny under a generalized time-reversible model. Parsnp requires a reference to calculate the core SNPs shared by all isolates: complete reference genomes from 5 different strains were used separately (accession numbers: NC_021485, strain 09mas018883 [CC1]; NC_007432, strain A909 [CC6]; HG939456, strain COH1 [CC17]; NC_018646, strain GD201008 [CC6]; NC_004368, strain NEM316 [CC23]). Trees were rooted at midpoint. Visualization of the phylogenetic analysis was performed via iTol (74). The recombination effect on phylogeny was assessed via ClonalFrameML ver. 1.12 (75) on five 140-strain random subsets of the entire data set.

### Pangenome construction and genome-wide association analysis.

A pangenome was generated from the combined human (African isolates from Malawi and Kenya [64], Canadian [32], American [65], and Dutch isolates, and isolates of unkown geographical origin [Table 1])- and animal-derived strains using Roary (ver. 3.8.0). Parameters for each run were: 95% of minimum BLASTp identity; MLC inflation value 1.5; with 99% of strains in which a gene must be present to be considered “core.”

Recently, several pipelines were developed for bacterial genome wide association studies (GWAS), such as PLINK, PhyC, ROADTRIPS, and SEER (76–79). Scoary (80) was designed to highlight genes in the accessory pangenome of a bacterial data set associated with a particular bacterial phenotype. Here, Scoary (ver. 1.6.16) was used to establish which genes were typical of each CC via a pan-genome-wide association study (pan-GWAS). The CC of each isolate was depicted as a discrete phenotype, e.g., belonging to CC17 or not, and defined as “positive” or “negative,” respectively, with the Scoary algorithm evaluating which gene feature is statistically associated with a particular CC (80). The cutoff for a significant association was a *P* value lower than 1e−10 and a sensitivity and specificity greater than 90%. CC-associated genes were plotted on the circular representation of the chromosome of 5 GBS isolates belonging to each CC (strains ST-1, NCTC8187, 2603V/R, SGM4, 874391, and NGBS572) using BRIG (ver. 0.8) (81). Gene synteny was also evaluated for the CC-associated genes; three genomes belonging to each CC and the genes identified in the pan-GWAS analysis were aligned using ProgressiveMauve (82). Mauve (ver. 2.3.1) (83) was used to produce a graphical representation of the alignment and gene synteny was qualitatively evaluated.

The sequence diversity of genes identified from the pan-GWAS analysis was investigated by selecting one representative nucleotide gene sequence associated with each CC (sequences reported in supplemental material file 1) and aligning this against each genome included in the analysis using BLASTn. The bitscore value of each gene alignment was used to produce the heatmaps shown in Fig. S2, using the R package pheatmap (ver. 1.0.10; https://CRAN.R-project.org/package=pheatmap). Bitscores were normalized against the highest scoring isolate for each gene: the normalized bitscore was 0 > x ≥ 1, where 1 corresponds to the highest identified bitscore, 0 corresponds to the absence of the gene, and values in between highlight a different level of gene similarity. For the identification of alleles that distinguish strains isolated from disease and carriage, we calculated the allelic profiles of the genes identified by the pan-GWAS pipeline in 545 CC17 strains (for which the isolation source was non-animal and known). For each gene we selected the alleles present in at least 10 strains and calculated the proportion of strains isolated from invasive source and carriage. Significance for alleles unevenly distributed between carriage and disease was calculated with a Fisher test.

### Assessment of ABC transporter activity by MIC.

Routine expansion of S. agalactiae strains was done in Todd-Hewitt (TH) broth or on TH agar (TH broth with 15% agar) at 37^°^C, 5% CO_2_.

The MICs of erythromycin, chloramphenicol, norfloxacin, acriflavin, and berberine against S. agalactiae strains COH1, NEM316, 2603V/R, and H36B were determined using the broth microdilution method with MH-F broth (Mueller-Hinton broth supplemented with 5% clarified lysed horse blood and 20 mg/liter β-NAD), as recommended by EUCAST (84, 85) and described in Robertson et al. in 2005 (42).

Briefly, GBS strains grown statically in TH broth at 37^°^C, 5% CO_2_ to logarithmic phase were harvested and resuspended in prewarmed MH-F broth at ∼5 × 10^5^ CFU/ml. A bacterial suspension (50 μl) was added to individual wells of a 96-well plate containing 50 μl MH-F supplemented with various concentrations of antimicrobial compounds (ranging from 0.06 to 32 μg/ml). The MIC was read after incubation of the 96-well plate at 37^°^C, 5% CO_2_ for 18 to 24 h.

### Ethical approval for Malawi GBS collection.

Collection of carriage isolates was approved by the College of Medicine Research Ethics Committee (COMREC), University of Malawi (P.05/14/1574) and the Liverpool School of Tropical Medicine Research Ethics Committee (14.036). Invasive disease surveillance in Malawi was approved by COMREC (P.11/09/835 and P.08/14/1614).

## References

[1] Shabayek S, Spellerberg B 2018 Group B streptococcal colonization, molecular characteristics, and epidemiology. Frontiers Microbiol 9:437. doi:10.3389/fmicb.2018.00437. PMC5861770 29593684

[2] Dermer P, Lee C, Eggert J, Few B 2004 A history of neonatal group B streptococcus with its related morbidity and mortality rates in the United States. J Pediatr Nurs 19:357–363. doi:10.1016/j.pedn.2004.05.012. 15614260

[3] Dagnew AF, Cunnington MC, Dube Q, Edwards MS, French N, Heyderman RS, Madhi SA, Slobod K, Clemens S 2012 Variation in reported neonatal group B streptococcal disease incidence in developing countries. Clin Infect Dis 55:91–102. doi:10.1093/cid/cis395. 22523262

[4] Heyderman RS, Madhi SA, French N, Cutland C, Ngwira B, Kayambo D, Mboizi R, Koen A, Jose L, Olugbosi M, Wittke F, Slobod K, Dull PM 2016 Group B streptococcus vaccination in pregnant women with or without HIV in Africa: a non-randomised phase 2, open-label, multicentre trial. Lancet Infect Dis 16:546–555. doi:10.1016/S1473-3099(15)00484-3. 26869376 PMC4835545

[5] Nishihara Y, Dangor Z, French N, Madhi S, Heyderman R 2017 Challenges in reducing group B Streptococcus disease in African settings. Arch Dis Child 102:72–77. doi:10.1136/archdischild-2016-311419. 27831912 PMC5256401

[6] Edmond KM, Kortsalioudaki C, Scott S, Schrag SJ, Zaidi AK, Cousens S, Heath PT 2012 Group B streptococcal disease in infants aged younger than 3 months: systematic review and meta-analysis. Lancet 379:547–556. doi:10.1016/S0140-6736(11)61651-6. 22226047

[7] Berardi A, GBS Prevention Working Group, Emilia-Romagna, Rossi C, Lugli L, Creti R, Bacchi Reggiani ML, Lanari M, Memo L, Pedna MF, Venturelli C, Perrone E, Ciccia M, Tridapalli E, Piepoli M, Contiero R, Ferrari F 2013 Group B streptococcus late-onset disease: 2003–2010. Pediatrics 131:e361–e368. doi:10.1542/peds.2012-1231. 23296441

[8] Meyn LA, Krohn MA, Hillier SL 2009 Rectal colonization by group B Streptococcus as a predictor of vaginal colonization. Am J Obstet Gynecol :201–276. doi:10.1016/j.ajog.2009.02.011. 19371857 PMC2770838

[9] Rajagopal L 2009 Understanding the regulation of Group B Streptococcal virulence factors. Future Microbiol 4:201–221. doi:10.2217/17460913.4.2.201. 19257847 PMC2691590

[10] Lemire P, Houde M, Lecours MP, Fittipaldi N, Segura M 2012 Role of capsular polysaccharide in Group B Streptococccus interactions with dendritic cells. Microbes Infect 14:1064–1076. doi:10.1016/j.micinf.2012.05.015. 22683668

[11] Slotved HC, Kong F, Lambertsen L, Sauer S, Gilbert GL 2007 Serotype IX, a proposed new *Streptococcus agalactiae* serotype. J Clin Microbiol 45:2929–2936. doi:10.1128/JCM.00117-07. 17634306 PMC2045254

[12] Russell NJ, GBS Maternal Colonization Investigator Group, Seale AC, O’Driscoll M, O’Sullivan C, Bianchi-Jassir F, Gonzalez-Guarin J, Lawn JE, Baker CJ, Bartlett L, Cutland C, Gravett MG, Heath PT, Le Doare K, Madhi SA, Rubens CE, Schrag S, Sobanjo-ter Meulen A, Vekemans J, Saha SK, Ip M, Asturias E, Gaind R, Kumar P, Anthony B, Madrid L, Bassat Q, Zhu C, Luo M, Nagarjuna D, Majumder S, GBS Maternal Colonization Investigator Group. 2017 Maternal colonization with group B streptococcus and serotype distribution worldwide: systematic review and meta-analyses. Clin Infect Dis 65:S100–S111. doi:10.1093/cid/cix658. 29117327 PMC5848259

[13] Da Cunha V, Davies MR, Douarre PE, Rosinski-Chupin I, Margarit I, Spinali S, Perkins T, Lechat P, Dmytruk N, Sauvage E, Ma L, Romi B, Tichit M, Lopez-Sanchez MJ, Descorps-Declere S, Souche E, Buchrieser C, Trieu-Cuot P, Moszer I, Clermont D, Maione D, Bouchier C, McMillan DJ, Parkhill J, Telford JL, Dougan G, Walker MJ, DEVANI Consortium, Holden MTG, Poyart C, Glaser P, Melin P, Decheva A, Petrunov B, Kriz P, Berner R, Buchele A, Hufnagel M, Kunze M, Creti R, Baldassarri L, Orefici G, Berardi A, Granger JR, Fraile M, Afshar B, Efstratiou A 2014 *Streptococcus agalactiae* clones infecting humans were selected and fixed through the extensive use of tetracycline. Nat Commun 5:4544. doi:10.1038/ncomms5544. 25088811 PMC4538795

[14] Sørensen UBS, Poulsen K, Ghezzo C, Margarit I, Kilian M 2010 Emergence and global dissemination of host-specific *Streptococcus agalactiae* clones. mBio 1:1–3. doi:10.1128/mBio.00178-10. PMC2932510 20824105

[15] Manning SD, Lewis MA, Springman AC, Lehotzky E, Whittam TS, Davies HD 2008 Genotypic diversity and serotype distribution of group B streptococcus isolated from women before and after delivery. Clin Infect Dis 46:1829–1837. doi:10.1086/588296. 18462173 PMC9491394

[16] Teatero S, Ferrieri P, Martin I, Demczuk W, McGeer A, Fittipaldi N 2017 Serotype distribution, population structure, and antimicrobial resistance of group B streptococcus strains recovered from colonized pregnant women. J Clin Microbiol 55:412–422. doi:10.1128/JCM.01615-16. 27852675 PMC5277510

[17] Lamy MC, Dramsi S, Billoët A, Réglier-Poupet H, Tazi A, Raymond J, Guérin F, Couvé E, Kunst F, Glaser P, Trieu-Cuot P, Poyart C 2006 Rapid detection of the “highly virulent” group B streptococcus ST-17 clone. Microbes Infect 8:1714–1722. doi:10.1016/j.micinf.2006.02.008. 16822689

[18] Bisharat N, Crook DW, Leigh J, Harding RM, Ward PN, Coffey TJ, Maiden MC, Peto T, Jones N 2004 Hyperinvasive neonatal group B streptococcus has arisen from a bovine ancestor. J Clin Microbiol 42:2161–2167. doi:10.1128/jcm.42.5.2161-2167.2004. 15131184 PMC404684

[19] Nobbs AH, Lamont RJ, Jenkinson HF 2009 Streptococcus adherence and colonization. Microbiol Mol Biol Rev 73:407–450. doi:10.1128/MMBR.00014-09. 19721085 PMC2738137

[20] Rosini R, Margarit I 2015 Biofilm formation by *Streptococcus agalactiae*: influence of environmental conditions and implicated virulence factor. Front Cell Infect Microbiol 5:6. doi:10.3389/fcimb.2015.00006. 25699242 PMC4316791

[21] Landwehr-Kenzel S, Henneke P 2014 Interaction of *Streptococcus agalactiae* and cellular innate immunity in colonization and disease. Front Immunol 5:519. doi:10.3389/fimmu.2014.00519. 25400631 PMC4212683

[22] Cheng Q, Stafslien D, Purushothaman SS, Cleary P 2002 The group B streptococcal C5a peptidase is both a specific protease and an invasin. Infect Immun 70:2408–2413. doi:10.1128/iai.70.5.2408-2413.2002. 11953377 PMC127948

[23] Santi I, Grifantini R, Jiang SM, Brettoni C, Grandi G, Wessels MR, Soriani M 2009 CsrRS regulates group B Streptococcus virulence gene expression in response to environmental pH: a new perspective on vaccine development. J Bacteriol 191:5387–5397. doi:10.1128/JB.00370-09. 19542277 PMC2725634

[24] Konto-Ghiorghi Y, Mairey E, Mallet A, Duménil G, Caliot E, Trieu-Cuot P, Dramsi S 2009 Dual role for pilus in adherence to epithelial cells and biofilm formation in *Streptococcus agalactiae*. PLoS Pathog 5:e1000422. doi:10.1371/journal.ppat.1000422. 19424490 PMC2674936

[25] Di Xia F, Mallet A, Caliot E, Gao C, Trieu-Cuot P, Dramsi S 2015 Capsular polysaccharide of Group B Streptococcus mediates biofilm formation in the presence of human plasma. Microbes Infect 17:71–76. doi:10.1016/j.micinf.2014.10.007. 25448634

[26] Patras KA, Derieux J, Al-Bassam MM, Adiletta N, Vrbanac A, Lapek JD, Zengler K, Gonzalez DJ, Nizet V 2018 Group B streptococcus biofilm regulatory protein A contributes to bacterial physiology and innate immune resistance. J Infect Dis 218:1641–1652. doi:10.1093/infdis/jiy341. 29868829 PMC6173572

[27] Buscetta M, Papasergi S, Firon A, Pietrocola G, Biondo C, Mancuso G, Midiri A, Romeo L, Teti G, Speziale P, Trieu-Cuot P, Beninati C 2014 FbsC, a novel fibrinogen-binding protein, promotes *Streptococcus agalactiae*-host cell interactions. J Biol Chem 289:21003–21015. doi:10.1074/jbc.M114.553073. 24904056 PMC4110306

[28] Patras KA, Wang NY, Fletcher EM, Cavaco CK, Jimenez A, Garg M, Fierer J, Sheen TR, Rajagopal L, Doran KS 2013 Group B Streptococcus CovR regulation modulates host immune signalling pathways to promote vaginal colonization. Cell Microbiol 15:1154–1167. doi:10.1111/cmi.12105. 23298320 PMC3657335

[29] Almeida A, Alves-Barroco C, Sauvage E, Bexiga R, Albuquerque P, Tavares F, Santos-Sanches I, Glaser P 2016 Persistence of a dominant bovine lineage of group B streptococcus reveals genomic signatures of host adaptation. Environ Microbiol 18:4216–4229. doi:10.1111/1462-2920.13550. 27696631

[30] Almeida A, Rosinski-Chupin I, Plainvert C, Douarre P-E, Borrego MJ, Poyart C, Glaser P 2017 Parallel evolution of group B streptococcus hypervirulent clonal complex 17 unveils new pathoadaptive mutations. mSystems 2:e00074-17. doi:10.1128/mSystems.00074-17. 28904998 PMC5585690

[31] Teatero S, McGeer A, Low DE, Li A, Demczuk W, Martin I, Fittipaldi N 2014 Characterization of invasive group B Streptococcus strains from the greater Toronto area, Canada. J Clin Microbiol 52:1441–1447. doi:10.1128/JCM.03554-13. 24554752 PMC3993709

[32] Page AJ, Cummins CA, Hunt M, Wong VK, Reuter S, Holden MTG, Fookes M, Falush D, Keane JA, Parkhill J 2015 Roary: rapid large-scale prokaryote pan genome analysis. Bioinformatics 31:3691–3693. doi:10.1093/bioinformatics/btv421. 26198102 PMC4817141

[33] Bray BA, Sutcliffe IC, Harrington DJ 2009 Expression of the MtsA lipoprotein of *Streptococcus agalactiae* A909 is regulated by manganese and iron. Antonie Van Leeuwenhoek 95:101–109. doi:10.1007/s10482-008-9291-6. 18982279

[34] Janulczyk R, Ricci S, Björck L 2003 MtsABC is important for manganese and iron transport, oxidative stress resistance, and virulence of *Streptococcus pyogenes*. Infect Immun 71:2656–2664. doi:10.1128/iai.71.5.2656-2664.2003. 12704140 PMC153223

[35] Kehl-Fie TE, Zhang Y, Moore JL, Farrand AJ, Hood MI, Rathi S, Chazin WJ, Caprioli RM, Skaar EP 2013 MntABC and MntH contribute to systemic *Staphylococcus aureus* infection by competing with calprotectin for nutrient manganese. Infect Immun 81:3395–3405. doi:10.1128/IAI.00420-13. 23817615 PMC3754211

[36] Paterson GK, Blue CE, Mitchell TJ 2006 Role of two-component systems in the virulence of *Streptococcus pneumoniae*. J Med Microbiol 55:355–363. doi:10.1099/jmm.0.46423-0. 16533981

[37] Lier C, Baticle E, Horvath P, Haguenoer E, Valentin AS, Glaser P, Mereghetti L, Lanotte P 2015 Analysis of the type II-A CRISPR-Cas system of *Streptococcus agalactiae* reveals distinctive features according to genetic lineages. Front Genet 6:214. doi:10.3389/fgene.2015.00214. 26124774 PMC4466440

[38] Chan WT, Yeo CC, Sadowy E, Espinosa M 2014 Functional validation of putative toxin-antitoxin genes from the Gram-positive pathogen *Streptococcus pneumoniae*: phd-doc is the fourth bona-fide operon. Front Microbiol 5:677. doi:10.3389/fmicb.2014.00677. 25538695 PMC4257102

[39] Van Melderen L 2010 Toxin-antitoxin systems: why so many, what for? Curr Opin Microbiol 13:781–785. doi:10.1016/j.mib.2010.10.006. 21041110

[40] Périchon B, Szili N, Du Merle L, Rosinski-Chupin I, Gominet M, Bellais S, Poyart C, Trieu-Cuot P, Dramsi S 2017 Regulation of PI-2b pilus expression in hypervirulent *Streptococcus agalactiae* ST-17 BM110. PLoS One 12:e0169840. doi:10.1371/journal.pone.0169840. 28107386 PMC5249243

[41] Obolski U, Gori A, Lourenço J, Thompson C, Thompson R, French N, Heyderman RS, Gupta S 2019 Identifying genes associated with invasive disease in *S. pneumoniae* by applying a machine learning approach to whole genome sequence typing data. Sci Rep 9:4049. doi:10.1038/s41598-019-40346-7. 30858412 PMC6411942

[42] Robertson GT, Doyle TB, Lynch AS 2005 Use of an efflux-deficient *Streptococcus pneumoniae* strain panel to identify ABC-class multidrug transporters involved in intrinsic resistance to antimicrobial agents. Antimicrob Agents Chemother 49:4781–4783. doi:10.1128/AAC.49.11.4781-4783.2005. 16251330 PMC1280156

[43] Remy L, Carrière M, Derré-Bobillot A, Martini C, Sanguinetti M, Borezée-Durant E 2013 The *Staphylococcus aureus* Opp1 ABC transporter imports nickel and cobalt in zinc-depleted conditions and contributes to virulence. Mol Microbiol 87:730–743. doi:10.1111/mmi.12126. 23279021

[44] Smith AW, Roche H, Trombe M-C, Briles DE, Håkansson A 2002 Characterization of the dihydrolipoamide dehydrogenase from *Streptococcus pneumoniae* and its role in pneumococcal infection. Mol Microbiol 44:431–448. doi:10.1046/j.1365-2958.2002.02883.x. 11972781

[45] Lindahl G, Stålhammar-Carlemalm M, Areschoug T 2005 Surface proteins of *Streptococcus agalactiae* and related proteins in other bacterial pathogens. Clin Microbiol Rev 18:102–127. doi:10.1128/CMR.18.1.102-127.2005. 15653821 PMC544178

[46] Lalioui L, Pellegrini E, Dramsi S, Baptista M, Bourgeois N, Doucet-Populaire F, Rusniok C, Zouine M, Glaser P, Kunst F, Poyart C, Trieu-Cuot P 2005 The SrtA sortase of *Streptococcus agalactiae* is required for cell wall anchoring of proteins containing the LPXTG motif, for adhesion to epithelial cells, and for colonization of the mouse intestine. Infect Immun 73:3342–3350. doi:10.1128/IAI.73.6.3342-3350.2005. 15908360 PMC1111822

[47] Romero-Hernández B, Tedim AP, Sánchez-Herrero JF, Librado P, Rozas J, Muñoz G, Baquero F, Cantón R, Del Campo R 2015 *Streptococcus gallolyticus* subsp. *gallolyticus* from human and animal origins: genetic diversity, antimicrobial susceptibility, and characterization of a vancomycin-resistant calf isolate carrying a *vanA*-Tn1546-like element. Antimicrob Agents Chemother 59:2006–2015. doi:10.1128/AAC.04083-14. 25605355 PMC4356806

[48] Berg BR, Houseman JL, TerSteeg ZE, LeBar WD, Newton DW 2014 Antimicrobial susceptibilities of group B streptococcus isolates from prenatal screening samples. J Clin Microbiol 52:3499–3501. doi:10.1128/JCM.01781-14. 25031448 PMC4313155

[49] Moellering RC 2006 Vancomycin: a 50-year reassessment. Clin Infect Dis 42:S3–S4. doi:10.1086/491708. 16323117

[50] Vaillancourt K, Moineau S, Frenette M, Lessard C, Vadeboncoeur C 2002 Galactose and lactose genes from the galactose-positive bacterium *Streptococcus salivarius* and the phylogenetically related galactose-negative bacterium *Streptococcus thermophilus*: organization, sequence, transcription, and activity of the gal gene products. J Bacteriol 184:785–793. doi:10.1128/jb.184.3.785-793.2002. 11790749 PMC139519

[51] Abranches J, Chen YYM, Burne RA 2004 Galactose metabolism by *Streptococcus mutans*. Appl Environ Microbiol 70:6047–6052. doi:10.1128/AEM.70.10.6047-6052.2004. 15466549 PMC522122

[52] Anbukkarasi K, Nanda DK, UmaMaheswari T, Hemalatha T, Singh P, Singh R 2014 Assessment of expression of Leloir pathway genes in wild-type galactose-fermenting *Streptococcus thermophilus* by real-time PCR. Eur Food Res Technol 239:895–903. doi:10.1007/s00217-014-2286-9.

[53] Carvalho SM, Kloosterman TG, Kuipers OP, Neves AR 2011 CcpA ensures optimal metabolic fitness of *Streptococcus pneumoniae*. PLoS One 6:e26707. doi:10.1371/journal.pone.0026707. 22039538 PMC3198803

[54] Zhang W, Rong C, Chen C, Gao GF 2012 Type-IVC secretion system: a novel subclass of type IV secretion system (T4SS) common existing in Gram-positive genus Streptococcus. PLoS One 7:e46390. doi:10.1371/journal.pone.0046390. 23056296 PMC3464263

[55] Alvarez-Martinez CE, Christie PJ 2009 Biological diversity of prokaryotic type IV secretion systems. Microbiol Mol Biol Rev 73:775–808. doi:10.1128/MMBR.00023-09. 19946141 PMC2786583

[56] Wallden K, Rivera-Calzada A, Waksman G 2010 Type IV secretion systems: versatility and diversity in function. Cell Microbiol 12:1203–1212. doi:10.1111/j.1462-5822.2010.01499.x. 20642798 PMC3070162

[57] Botelho ACN, Ferreira AFM, Fracalanzza SEL, Teixeira LM, Pinto T 2018 A perspective on the potential zoonotic role of *Streptococcus agalactiae*: searching for a missing link in alternative transmission routes. Front Microbiol 9:608. doi:10.3389/fmicb.2018.00608. 29643850 PMC5882794

[58] Zadoks RN, Middleton JR, McDougall S, Katholm J, Schukken YH 2011 Molecular epidemiology of mastitis pathogens of dairy cattle and comparative relevance to humans. J Mammary Gland Biol Neoplasia 16:357–372. doi:10.1007/s10911-011-9236-y. 21968538 PMC3208832

[59] Lyhs U, Kulkas L, Katholm J, Waller KP, Saha K, Tomusk RJ, Zadoks RN 2016 *Streptococcus agalactiae* serotype IV in humans and cattle, Northern Europe. Emerg Infect Dis 22:2097–2103. doi:10.3201/eid2212.151447. 27869599 PMC5189126

[60] Manning SD, Springman AC, Million AD, Milton NR, McNamara SE, Somsel PA, Bartlett P, Davies HD 2010 Association of group B Streptococcus colonization and bovine exposure: a prospective cross-sectional cohort study. PLoS One 5:e8795. doi:10.1371/journal.pone.0008795. 20098699 PMC2808344

[61] Chen M, Wang R, Luo FG, Huang Y, Liang WW, Huang T, Lei AY, Gan X, Li LP 2015 *Streptococcus agalactiae* isolates of serotypes Ia, III and V from human and cow are able to infect tilapia. Vet Microbiol 180:129–135. doi:10.1016/j.vetmic.2015.07.033. 26255553

[62] Rosinski-Chupin I, Sauvage E, Mairey B, Mangenot S, Ma L, Da Cunha V, Rusniok C, Bouchier C, Barbe V, Glaser P 2013 Reductive evolution in *Streptococcus agalactiae* and the emergence of a host adapted lineage. BMC Genomics 14:252. doi:10.1186/1471-2164-14-252. 23586779 PMC3637634

[63] Seale AC, Koech AC, Sheppard AE, Barsosio HC, Langat J, Anyango E, Mwakio S, Mwarumba S, Morpeth SC, Anampiu K, Vaughan A, Giess A, Mogeni P, Walusuna L, Mwangudzah H, Mwanzui D, Salim M, Kemp B, Jones C, Mturi N, Tsofa B, Mumbo E, Mulewa D, Bandika V, Soita M, Owiti M, Onzere N, Walker AS, Schrag SJ, Kennedy SH, Fegan G, Crook DW, Berkley JA 2016 Maternal colonization with *Streptococcus agalactiae* and associated stillbirth and neonatal disease in coastal Kenya. Nat Microbiol 1:16067. doi:10.1038/nmicrobiol.2016.67. 27572968 PMC4936517

[64] Flores AR, Galloway-Peña J, Sahasrabhojane P, Saldaña M, Yao H, Su X, Ajami NJ, Holder ME, Petrosino JF, Thompson E, Ros IMY, Rosini R, Grandi G, Horstmann N, Teatero S, McGeer A, Fittipaldi N, Rappuoli R, Baker CJ, Shelburne SA 2015 Sequence type 1 group B Streptococcus, an emerging cause of invasive disease in adults, evolves by small genetic changes. Proc Natl Acad Sci U S A 112:6431–6436. doi:10.1073/pnas.1504725112. 25941374 PMC4443349

[65] Jamrozy D, de Goffau MC, Bijlsma MW, van de Beek D, Kuijpers TW, Parkhill J, van der Ende A, Bentley SD 2018 Temporal population structure of invasive Group B Streptococcus during a period of rising disease incidence shows expansion of a CC17 clone. bioRxiv 447037. doi:10.1101/447037.

[66] Bolger AM, Lohse M, Usadel B 2014 Trimmomatic: a flexible trimmer for Illumina sequence data. Bioinformatics 30:2114–2120. doi:10.1093/bioinformatics/btu170. 24695404 PMC4103590

[67] Bankevich A, Nurk S, Antipov D, Gurevich AA, Dvorkin M, Kulikov AS, Lesin VM, Nikolenko SI, Pham S, Prjibelski AD, Pyshkin AV, Sirotkin AV, Vyahhi N, Tesler G, Alekseyev MA, Pevzner PA 2012 SPAdes: a new genome assembly algorithm and its applications to single-cell sequencing. J Comput Biol 19:455–477. doi:10.1089/cmb.2012.0021. 22506599 PMC3342519

[68] Seemann T 2014 Prokka: rapid prokaryotic genome annotation. Bioinformatics 30:2068–2069. doi:10.1093/bioinformatics/btu153. 24642063

[69] Wood DE, Salzberg SL 2014 Kraken: ultrafast metagenomic sequence classification using exact alignments. Genome Biol 15:R46. doi:10.1186/gb-2014-15-3-r46. 24580807 PMC4053813

[70] Jolley KA, Maiden MC 2010 BIGSdb: scalable analysis of bacterial genome variation at the population level. BMC Bioinformatics 11:595. doi:10.1186/1471-2105-11-595. 21143983 PMC3004885

[71] Enright MC, Robinson DA, Randle G, Feil EJ, Grundmann H, Spratt BG 2002 The evolutionary history of methicillin-resistant *Staphylococcus aureus* (MRSA). Proc Natl Acad Sci U S A 99:7687–7692. doi:10.1073/pnas.122108599. 12032344 PMC124322

[72] Treangen TJ, Ondov BD, Koren S, Phillippy AM 2014 The Harvest suite for rapid core-genome alignment and visualization of thousands of intraspecific microbial genomes. Genome Biol 15:524. doi:10.1186/s13059-014-0524-x. 25410596 PMC4262987

[73] Price MN, Dehal PS, Arkin AP 2010 FastTree 2—approximately maximum-likelihood trees for large alignments. PLoS One 5:e9490. doi:10.1371/journal.pone.0009490. 20224823 PMC2835736

[74] Letunic I, Bork P 2016 Interactive Tree Of Life (iTOL) v3: an online tool for the display and annotation of phylogenetic and other trees. Nucleic Acids Res 44:W242–W245. doi:10.1093/nar/gkw290. 27095192 PMC4987883

[75] Didelot X, Wilson DJ 2015 ClonalFrameML: efficient inference of recombination in whole bacterial genomes. PLoS Comput Biol 11:e1004041. doi:10.1371/journal.pcbi.1004041. 25675341 PMC4326465

[76] Chen PE, Shapiro BJ 2015 The advent of genome-wide association studies for bacteria. Curr Opin Microbiol 25:17–24. doi:10.1016/j.mib.2015.03.002. 25835153

[77] Chang CC, Chow CC, Tellier LC, Vattikuti S, Purcell SM, Lee JJ 2015 Second-generation PLINK: rising to the challenge of larger and richer datasets. Gigascience 4:7. doi:10.1186/s13742-015-0047-8. 25722852 PMC4342193

[78] Thornton T, McPeek MS 2010 ROADTRIPS: case-control association testing with partially or completely unknown population and pedigree structure. Am J Hum Genet 86:172–184. doi:10.1016/j.ajhg.2010.01.001. 20137780 PMC2820184

[79] Lees JA, Vehkala M, Välimäki N, Harris SR, Chewapreecha C, Croucher NJ, Marttinen P, Davies MR, Steer AC, Tong SYC, Honkela A, Parkhill J, Bentley SD, Corander J 2016 Sequence element enrichment analysis to determine the genetic basis of bacterial phenotypes. Nat Commun 7:12797. doi:10.1038/ncomms12797. 27633831 PMC5028413

[80] Brynildsrud O, Bohlin J, Scheffer L, Eldholm V 2016 Rapid scoring of genes in microbial pan-genome-wide association studies with Scoary. Genome Biol 17:238. doi:10.1186/s13059-016-1108-8. 27887642 PMC5124306

[81] Alikhan NF, Petty NK, Ben Zakour NL, Beatson SA 2011 BLAST Ring Image Generator (BRIG): simple prokaryote genome comparisons. BMC Genomics 12:402. doi:10.1186/1471-2164-12-402. 21824423 PMC3163573

[82] Darling AE, Mau B, Perna NT 2010 progressiveMauve: multiple genome alignment with gene gain, loss and rearrangement. PLoS One 5:e11147. doi:10.1371/journal.pone.0011147. 20593022 PMC2892488

[83] Darling AE, Mau B, Blattner FR, Perna NT 2004 Mauve: multiple alignment of conserved genomic sequence with rearrangements. Genome Res 14:1394–1403. doi:10.1101/gr.2289704. 15231754 PMC442156

[84] Andrews JM 2001 Determination of minimum inhibitory concentrations. J Antimicrob Chemother 48:5–16. doi:10.1093/jac/48.suppl_1.5. 11420333

[85] Wiegand I, Hilpert K, Hancock R 2008 Agar and broth dilution methods to determine the minimal inhibitory concentration (MIC) of antimicrobial substances. Nat Protoc 3:163–175. doi:10.1038/nprot.2007.521. 18274517

