# Supplementary material for: Pan-GWAS of Streptococcus agalactiae Highlights Lineage-Specific Genes Associated with Virulence and Niche Adaptation
Source: mBio. 2020 Jun 9;11(3):e00728-20. doi: 10.1128/mBio.00728-20 (PMC7373188; doi:10.1128/mBio.00728-20)
Supplement: TABLE S1 [file mBio.00728-20-st001.pdf]

Name	CC	Source	country	Serotype	Year	MLST-Type	ENA Acc. Number
1000_NETGBS_STREPCAR_145	CC23	carrier	Malawi	V	2008	24	ERR840684
1001_NETGBS_C10285	CC17	invasive	Malawi	III	2004	17	ERR840685
1002_NETGBS_STREPCAR_158	CC17	carrier	Malawi	III	2008	17	ERR840686
1003_NETGBS_C10367	CC17	invasive	Malawi	III	2004	17	ERR840687
1004_NETGBS_STREPCAR_182	CC23	carrier	Malawi	Ia	2008	23	ERR840688
1005_NETGBS_D27359	CC17	invasive	Malawi	III	2004	17	ERR840689
1006_NETGBS_STREPCAR_189	CC19	carrier	Malawi	V	2008	327	ERR840690
1007_NETGBS_D27967	CC17	invasive	Malawi	III	2004	17	ERR840691
1008_NETGBS_STREPCAR_191	CC17	carrier	Malawi	III	2008	17	ERR840692
1009_NETGBS_D27979A	CC19	invasive	Malawi	III	2004	28	ERR840693
1010_NETGBS_STREPCAR_194	CC23	carrier	Malawi	V	2008	24	ERR840694
1011_NETGBS_D28080	CC17	invasive	Malawi	III	2004	867	ERR840695
1012_NETGBS_STREPCAR_206	CC23	carrier	Malawi	Ia	2008	23	ERR840696
1013_NETGBS_D28665	CC17	invasive	Malawi	III	2004	17	ERR840697
1014_NETGBS_STREPCAR_258	CC23	carrier	Malawi	V	2009	24	ERR840698
1015_NETGBS_C11288	CC17	invasive	Malawi	III	2005	109	ERR840699
1016_NETGBS_STREPCAR_266	CC23	carrier	Malawi	Ia	2009	23	ERR840700
1017_NETGBS_D31106	CC23	invasive	Malawi	Ia	2005	23	ERR840701
1018_NETGBS_STREPCAR_279	CC17	carrier	Malawi	III	2009	17	ERR840702
1020_NETGBS_STREPCAR_284	CC23	carrier	Malawi	Ia	2009	23	ERR840704
1021_NETGBS_D31426	CC17	invasive	Malawi	III	2005	17	ERR840705
1022_NETGBS_STREPCAR_289	CC23	carrier	Malawi	V	2009	24	ERR840706
1023_NETGBS_C11802	CC17	invasive	Malawi	III	2005	17	ERR840707
1024_NETGBS_STREPCAR_306	CC1	carrier	Malawi	Ia	2009	1	ERR840708
1025_NETGBS_D31476	CC17	invasive	Malawi	III	2005	17	ERR840709
1026_NETGBS_STREPCAR_307	CC23	carrier	Malawi	V	2009	23	ERR840710
1027_NETGBS_C11826	CC17	invasive	Malawi	III	2005	17	ERR840711
1028_NETGBS_STREPCAR_313	CC23	carrier	Malawi	Ia	2009	23	ERR840712
1029_NETGBS_D31518	CC17	invasive	Malawi	III	2005	17	ERR840713
1030_NETGBS_STREPCAR_315	CC17	carrier	Malawi	III	2009	17	ERR840714
1032_NETGBS_STREPCAR_324	CC17	carrier	Malawi	III	2009	17	ERR840716
1033_NETGBS_D31771	N/D	invasive	Malawi	II	2005		ERR840717
1034_NETGBS_STREPCAR_325	CC19	carrier	Malawi	V	2009	327	ERR840718
1035_NETGBS_D31823	CC17	invasive	Malawi	III	2005	17	ERR840719
1038_NETGBS_STREPCAR_356	CC17	carrier	Malawi	III	2009	17	ERR840722
1039_NETGBS_D32679	CC23	invasive	Malawi	Ia	2005	23	ERR840723
1040_NETGBS_STREPCAR_394	CC17	carrier	Malawi	III	2009	17	ERR840724
1041_NETGBS_C12327	CC17	invasive	Malawi	III	2005	17	ERR840725
1043_NETGBS_D32872	CC17	invasive	Malawi	III	2005	17	ERR840727
1045_NETGBS_C12445	CC17	invasive	Malawi	III	2005	17	ERR840729
1049_NETGBS_C12548	CC17	invasive	Malawi	III	2005	17	ERR840733
1050_NETGBS_STREPCAR_429	CC19	carrier	Malawi	V	2009	327	ERR840734
1051_NETGBS_D33430	CC17	invasive	Malawi	III	2005	17	ERR840735
1053_NETGBS_D34216	CC17	invasive	Malawi	III	2005	17	ERR840737
1054_NETGBS_STREPCAR_450	CC23	carrier	Malawi	Ia	2009	23	ERR840738
1055_NETGBS_C12852	CC17	invasive	Malawi	III	2005	17	ERR840739
1056_NETGBS_STREPCAR_463	CC23	carrier	Malawi	Ia	2009	23	ERR840740
1058_NETGBS_STREPCAR_467	CC23	carrier	Malawi	Ia	2009	23	ERR840742
1059_NETGBS_D35054	CC17	invasive	Malawi	III	2005	866	ERR840743
1061_NETGBS_C13174	CC17	invasive	Malawi	III	2005	17	ERR840745
1063_NETGBS_D35392	CC17	invasive	Malawi	III	2005	17	ERR840747
1065_NETGBS_D36580	CC17	invasive	Malawi	III	2006	17	ERR840749
1066_NETGBS_STREPCAR_529	CC23	carrier	Malawi	V	2009	223	ERR840750
1067_NETGBS_D36637	CC17	invasive	Malawi	III	2006	17	ERR840751
1068_NETGBS_STREPCAR_531	CC23	carrier	Malawi	Ia	2009	23	ERR840752
1069_NETGBS_D37811	CC17	invasive	Malawi	III	2006	17	ERR840753
1070_NETGBS_STREPCAR_561	CC17	carrier	Malawi	V	2009	17	ERR840754
1071_NETGBS_C13986	CC17	invasive	Malawi	III	2006	17	ERR840755
1072_NETGBS_STREPCAR_565	CC19	carrier	Malawi	V	2009	327	ERR840756
1073_NETGBS_C14243	CC17	invasive	Malawi	III	2006	17	ERR840757
1074_NETGBS_STREPCAR_566	CC19	carrier	Malawi	V	2009	327	ERR840758

1075_NETGBS_D38644	CC17	invasive	Malawi	III	2006	17	ERR840759
1079_NETGBS_D39631	CC17	invasive	Malawi	III	2006	17	ERR840763
1082_NETGBS_STREPCAR_609	CC17	carrier	Malawi	III	2009	17	ERR840766
1083_NETGBS_D40942	CC17	invasive	Malawi	III	2007	17	ERR840767
1084_NETGBS_STREPCAR_633	CC23	carrier	Malawi	Ia	2009	23	ERR840768
1085_NETGBS_D43106	CC17	invasive	Malawi	III	2007	868	ERR840769
1086_NETGBS_STREPCAR_636	CC23	carrier	Malawi	Ia	2009	23	ERR840770
1087_NETGBS_D44762	CC17	invasive	Malawi	III	2007	17	ERR840771
1088_NETGBS_STREPCAR_639	CC23	carrier	Malawi	Ia	2009	23	ERR840772
1089_NETGBS_C17687	CC17	invasive	Malawi	III	2008	17	ERR840773
1090_NETGBS_STREPCAR_640	CC23	carrier	Malawi	Ia	2009	23	ERR840774
1091_NETGBS_C17799	CC17	invasive	Malawi	III	2008	17	ERR840775
1092_NETGBS_STREPCAR_643	CC17	carrier	Malawi	III	2009	17	ERR840776
1093_NETGBS_D46721	CC17	invasive	Malawi	III	2008	17	ERR840777
1095_NETGBS_D47658	CC17	invasive	Malawi	III	2008	17	ERR840779
1099_NETGBS_D48747	CC17	invasive	Malawi	III	2008	17	ERR840783
1100_NETGBS_STREPCAR_822	CC19	carrier	Malawi	V	2009	327	ERR840784
1101_NETGBS_D48955	CC10	invasive	Malawi	Ib	2008	10	ERR840785
1102_NETGBS_STREPCAR_823	N/D	carrier	Malawi	V	2009		ERR840786
1103_NETGBS_D49166	CC17	invasive	Malawi	III	2008	17	ERR840787
1104_NETGBS_STREPCAR_827	N/D	carrier	Malawi	III	2009		ERR840788
1105_NETGBS_D51502	CC17	invasive	Malawi	III	2009	17	ERR840789
1106_NETGBS_STREPCAR_875	CC23	carrier	Malawi	NT	2009	23	ERR840790
1107_NETGBS_D52468	CC19	invasive	Malawi	III	2009	28	ERR840791
1109_NETGBS_C21622	CC17	invasive	Malawi	III	2009	17	ERR840793
1110_NETGBS_STREPCAR_1028	CC17	carrier	Malawi	III	2009	17	ERR840794
1111_NETGBS_C15979	CC17	invasive	Malawi	III	2007	17	ERR840795
1112_NETGBS_STREPCAR_1043	CC17	carrier	Malawi	Ia	2009	17	ERR840796
1113_NETGBS_D50751	CC17	invasive	Malawi	III	2009	17	ERR840797
1114_NETGBS_STREPCAR_1067	CC17	carrier	Malawi	III	2009	17	ERR840798
1115_NETGBS_D52378	CC17	invasive	Malawi	III	2009	17	ERR840799
1116_NETGBS_STREPCAR_1137	CC17	carrier	Malawi	III	2009	869	ERR840800
1117_NETGBS_D55627	CC17	invasive	Malawi	III	2010	17	ERR840801
1118_NETGBS_STREPCAR_1149	CC17	carrier	Malawi	III	2009	17	ERR840802
1119_NETGBS_BCN103	CC17	invasive	Malawi	III	2010	17	ERR840803
1120_NETGBS_STREPCAR_1158	CC17	carrier	Malawi	III	2009	17	ERR840804
1122_NETGBS_STREPCAR_1159	CC23	carrier	Malawi	Ia	2009	23	ERR840806
1123_NETGBS_1011471	CC17	invasive	Malawi	III	2010	17	ERR840807
1124_NETGBS_STREPCAR_1196	CC19	carrier	Malawi	V	2009	327	ERR840808
1125_NETGBS_1014197	CC17	invasive	Malawi	III	2011	17	ERR840809
1126_NETGBS_STREPCAR_1203	CC19	carrier	Malawi	V	2009	327	ERR840810
1128_NETGBS_STREPCAR_1226	CC17	carrier	Malawi	III	2009	17	ERR840812
1129_NETGBS_BCN11Z	CC17	invasive	Malawi	III	2011	17	ERR840813
1131_NETGBS_1016321	CC17	invasive	Malawi	III	2011	17	ERR840815
1132_NETGBS_STREPCAR_1302	CC17	carrier	Malawi	III	2009	870	ERR840816
1133_NETGBS_1016523	CC17	invasive	Malawi	III	2011	17	ERR840817
1134_NETGBS_STREPCAR_1330	CC17	carrier	Malawi	III	2009	17	ERR840818
1136_NETGBS_1022709	CC17	invasive	Malawi	III	2011	17	ERR840820
1138_NETGBS_1024925	CC17	invasive	Malawi	III	2011	17	ERR840822
1139_NETGBS_STREPCAR_1370	CC17	carrier	Malawi	III	2009	17	ERR840823
1140_NETGBS_1025174	CC17	invasive	Malawi	III	2011	17	ERR840824
1141_NETGBS_STREPCAR_1374	CC19	carrier	Malawi	V	2009	327	ERR840825
1142_NETGBS_1029273	CC17	invasive	Malawi	III	2012	17	ERR840826
1143_NETGBS_STREPCAR_1435	CC23	carrier	Malawi	Ia	2009	23	ERR840827
1144_NETGBS_1032982	CC17	invasive	Malawi	III	2012	17	ERR840828
1145_NETGBS_STREPCAR_1448	CC19	carrier	Malawi	V	2009	327	ERR840829
1146_NETGBS_1035321	CC17	invasive	Malawi	III	2012	17	ERR840830
1148_NETGBS_1035358	CC17	invasive	Malawi	III	2012	17	ERR840832
1151_NETGBS_BKQ3RF	CC17	invasive	Malawi	III	2013	17	ERR840835
1152_NETGBS_STREPCAR_1543	CC17	carrier	Malawi	NT	2010	17	ERR840836
1153_NETGBS_BKQ4TM	CC17	invasive	Malawi	III	2013	866	ERR840837
1154_NETGBS_STREPCAR_1544	CC23	carrier	Malawi	V	2010	223	ERR840838

1155_NETGBS_BKR2NF	CC23	invasive	Malawi	Ia	2013	23	ERR840839
1156_NETGBS_STREPCAR_1547	CC17	carrier	Malawi	III	2010	17	ERR840840
1158_NETGBS_STREPCAR_1556	CC23	carrier	Malawi	V	2010	24	ERR840842
1159_NETGBS_BKQ6Q9	CC17	invasive	Malawi	III	2013	17	ERR840843
1163_NETGBS_BKQBUE	CC17	invasive	Malawi	III	2014	17	ERR840847
1272_GB00112	CC17	invasive	Canada	NT	1999	17	
1475_NGBS10	CC1	invasive	Canada	V	2009	1	SRR1790740
1476_NGBS107	CC1	invasive	Canada	V	2010	1	SRR1790741
1477_NGBS110	CC1	invasive	Canada	V	2010	1	SRR1790742
1478_NGBS117	CC1	invasive	Canada	V	2010	1	SRR1790743
1480_NGBS164	N/D	invasive	Canada	V	2010		SRR1790745
1481_NGBS171	CC1	invasive	Canada	V	2010	1	SRR1790746
1482_NGBS172	CC1	invasive	Canada	V	2010	1	SRR1790747
1483_NGBS177	CC1	invasive	Canada	V	2010	1	SRR1790748
1484_NGBS180	CC1	invasive	Canada	V	2010	1	SRR1790749
1485_NGBS200	CC1	invasive	Canada	V	2010	1	SRR1790750
1486_NGBS21	CC1	invasive	Canada	V	2009	1	SRR1790751
1487_NGBS210	CC1	invasive	Canada	V	2011	1	SRR1790752
1488_NGBS22	CC1	invasive	Canada	V	2009	1	SRR1790753
1490_NGBS234	N/D	invasive	Canada	V	2010		SRR1790755
1491_NGBS241	CC1	invasive	Canada	V	2011	453	SRR1790756
1492_NGBS244	CC1	invasive	Canada	V	2011	871	SRR1790757
1493_NGBS246	CC1	invasive	Canada	V	2011	1	SRR1790758
1494_NGBS25	CC1	invasive	Canada	V	2009	1	SRR1790759
1495_NGBS267	CC1	invasive	Canada	V	2010	1	SRR1790760
1496_NGBS272	CC1	invasive	Canada	V	2011	1	SRR1790761
1497_NGBS273	CC1	invasive	Canada	V	2011	1	SRR1790762
1498_NGBS275	CC1	invasive	Canada	V	2010	1	SRR1790763
1499_NGBS279	CC1	invasive	Canada	V	2010	1	SRR1790764
1500_NGBS28	CC1	invasive	Canada	V	2009	1	SRR1790765
1501_NGBS283	CC1	invasive	Canada	V	2010	1	SRR1790766
1502_NGBS287	CC1	invasive	Canada	V	2010	1	SRR1790767
1503_NGBS288	CC1	invasive	Canada	V	2010	1	SRR1790768
1504_NGBS298	CC1	invasive	Canada	V	2011	1	SRR1790769
1505_NGBS30	CC1	invasive	Canada	V	2010	1	SRR1790770
1506_NGBS303	CC1	invasive	Canada	V	2010	1	SRR1790771
1507_NGBS321	CC1	invasive	Canada	V	2011	1	SRR1790772
1508_NGBS323	CC1	invasive	Canada	V	2011	1	SRR1790773
1509_NGBS325	CC1	invasive	Canada	V	2011	1	SRR1790774
1510_NGBS330	CC1	invasive	Canada	V	2011	1	SRR1790775
1511_NGBS331	CC1	invasive	Canada	V	2011	1	SRR1790776
1512_NGBS332	CC1	invasive	Canada	V	2011	1	SRR1790777
1514_NGBS348	CC1	invasive	Canada	V	2011	1	SRR1790779
1515_NGBS35	CC1	invasive	Canada	V	2010	1	SRR1790780
1516_NGBS357	CC1	invasive	Canada	V	2011	1	SRR1790781
1517_NGBS359	CC1	invasive	Canada	V	2011	1	SRR1790782
1518_NGBS360	CC1	invasive	Canada	V	2011	1	SRR1790783
1519_NGBS372	CC1	invasive	Canada	V	2011	1	SRR1790784
1520_NGBS380	CC1	invasive	Canada	V	2011	1	SRR1790785
1521_NGBS381	CC1	invasive	Canada	V	2011	1	SRR1790786
1522_NGBS411	CC1	invasive	Canada	V	2011	1	SRR1790787
1523_NGBS418	CC1	invasive	Canada	V	2011	1	SRR1790788
1524_NGBS425	CC1	invasive	Canada	V	2011	1	SRR1790789
1526_NGBS444	CC1	invasive	Canada	V	2011	1	SRR1790792
1527_NGBS462	CC1	invasive	Canada	V	2011	1	SRR1790793
1528_NGBS492	CC1	invasive	Canada	V	2012	1	SRR1790794
1530_NGBS497	CC1	invasive	Canada	V	2012	1	SRR1790796
1531_NGBS499	CC1	invasive	Canada	V	2012	1	SRR1790797
1533_NGBS519	CC1	invasive	Canada	V	2012	1	SRR1790799
1534_NGBS536	CC1	invasive	Canada	V	2012	1	SRR1790800
1535_NGBS54	CC1	invasive	Canada	V	2010	1	SRR1790801
1536_NGBS553	CC1	invasive	Canada	V	2012	1	SRR1790802

1537_NGBS558	CC1	invasive	Canada	V	2012	1	SRR1790803
1538_NGBS561	CC1	invasive	Canada	V	2012	1	SRR1790804
1540_NGBS579	CC1	invasive	Canada	V	2012	1	SRR1790806
1541_NGBS580	CC1	invasive	Canada	V	2012	1	SRR1790807
1542_NGBS586	CC1	invasive	Canada	V	2012	1	SRR1790808
1543_NGBS604	CC1	invasive	Canada	V	2012	1	SRR1790809
1544_NGBS624	CC1	invasive	Canada	V	2012	1	SRR1790810
1545_NGBS63	CC1	invasive	Canada	V	2010	1	SRR1790811
1546_NGBS630	CC1	invasive	Canada	V	2012	1	SRR1790812
1547_NGBS633	CC1	invasive	Canada	V	2012	531	SRR1790813
1548_NGBS68	CC1	invasive	Canada	V	2010	1	SRR1790814
1549_NGBS8	CC1	invasive	Canada	V	2009	1	SRR1790815
1550_NGBS9	CC1	invasive	Canada	V	2010	1	SRR1790816
1551_NGBS92	CC1	invasive	Canada	V	2010	1	SRR1790817
1552_NGBS93	CC1	invasive	Canada	V	2010	1	SRR1790818
1553_NGBS94	CC1	invasive	Canada	V	2010	1	SRR1790819
1554_NGBS99	CC1	invasive	Canada	V	2010	1	SRR1790820
1555_SS100	CC1	invasive	USA	V	2006	1	SRR2981531
1556_SS102	CC1	invasive	USA	V	2006	1	SRR2981532
1557_SS103	CC1	invasive	USA	V	2006	1	SRR2981533
1558_SS104	CC1	invasive	USA	V	2006	1	SRR2981534
1560_SS106	CC1	invasive	USA	V	2006	1	SRR2981536
1561_SS107	CC1	invasive	USA	V	2006	1	SRR2981537
1562_SS108	CC1	invasive	USA	V	2006	872	SRR2981538
1563_SS109	CC1	invasive	USA	V	2006	1	SRR2981539
1564_SS110	CC1	invasive	USA	V	2007	1	SRR2981540
1565_SS111	CC1	invasive	USA	V	2007	1	SRR2981541
1566_SS114	CC1	invasive	USA	V	2007	1	SRR2981542
1567_SS115	CC1	invasive	USA	V	2007	1	SRR2981543
1568_SS116	CC1	invasive	USA	V	2007	1	SRR2981544
1570_SS119	CC1	invasive	USA	V	2008	1	SRR2981546
1571_SS120	CC1	invasive	USA	V	2008	1	SRR2981547
1572_SS122	CC1	invasive	USA	V	2009	1	SRR2981548
1573_SS125	CC1	invasive	USA	V	2009	1	SRR2981549
1574_SS126	CC1	invasive	USA	V	2009	1	SRR2981550
1575_SS127	CC1	invasive	USA	V	2009	1	SRR2981551
1576_SS129	CC1	invasive	USA	V	2009	1	SRR2981552
1577_SS132	CC1	invasive	USA	V	2009	1	SRR2981553
1578_SS133	CC1	invasive	USA	V	2009	1	SRR2981554
1579_SS135	CC1	invasive	USA	V	2007	1	SRR2981555
1580_SS136	CC1	invasive	USA	V	2007	297	SRR2981556
1581_SS138	CC1	invasive	USA	V	2010	1	SRR2981557
1582_SS140	CC1	invasive	USA	V	2008	1	SRR2981558
1583_SS141	CC1	invasive	USA	V	2009	1	SRR2981559
1584_SS143	CC1	invasive	USA	V	2010	1	SRR2981560
1585_SS144	CC1	invasive	USA	V	2010	1	SRR2981561
1586_SS145	CC1	invasive	USA	V	2010	1	SRR2981562
1587_SS146	CC1	invasive	USA	V	2010	1	SRR2981563
1588_SS147	CC1	invasive	USA	V	2010	1	SRR2981564
1589_SS148	CC1	invasive	USA	V	2011	1	SRR2981565
1590_SS150	CC1	invasive	USA	V	2012	1	SRR2981566
1591_SS151	CC1	invasive	USA	V	2012	1	SRR2981567
1592_SS152	CC1	invasive	USA	V	2012	1	SRR2981568
1593_SS31	CC1	invasive	USA	V	1992	1	SRR2981569
1594_SS32	CC1	invasive	USA	V	1992	1	SRR2981570
1595_SS33	CC1	invasive	USA	V	1994	1	SRR2981571
1596_SS34	CC1	invasive	USA	V	1994	1	SRR2981572
1597_SS35	CC1	invasive	USA	V	1994	1	SRR2981573
1598_SS36	CC1	invasive	USA	V	1997	1	SRR2981574
1599_SS37	CC1	invasive	USA	V	1997	1	SRR2981575
1600_SS38	CC1	invasive	USA	V	1998	1	SRR2981576
1601_SS39	CC1	invasive	USA	V	1998	1	SRR2981577

1602_SS40	CC1	invasive	USA	V	1998	1	SRR2981578
1603_SS41	CC1	invasive	USA	V	1998	1	SRR2981579
1604_SS42	CC1	invasive	USA	V	1998	1	SRR2981580
1605_SS43	CC1	invasive	USA	V	1998	1	SRR2981581
1606_SS44	CC1	invasive	USA	V	1999	1	SRR2981582
1607_SS45	CC1	invasive	USA	V	1999	1	SRR2981583
1608_SS46	CC1	invasive	USA	V	1999	1	SRR2981584
1609_SS47	CC1	invasive	USA	V	2000	1	SRR2981585
1610_SS48	CC1	invasive	USA	V	2000	1	SRR2981586
1611_SS49	CC1	invasive	USA	V	2000	873	SRR2981587
1612_SS50	CC1	invasive	USA	V	2000	1	SRR2981588
1613_SS51	CC1	invasive	USA	V	2000	1	SRR2981589
1614_SS52	CC1	invasive	USA	V	2000	1	SRR2981590
1615_SS53	CC1	invasive	USA	V	2000	1	SRR2981591
1616_SS54	CC1	invasive	USA	V	2001	1	SRR2981592
1617_SS56	CC1	invasive	USA	V	2001	1	SRR2981593
1618_SS57	CC1	invasive	USA	V	2001	1	SRR2981594
1619_SS58	CC1	invasive	USA	V	2001	1	SRR2981595
1620_SS59	CC1	invasive	USA	V	2001	1	SRR2981596
1621_SS60	CC1	invasive	USA	V	2001	1	SRR2981597
1622_SS61	CC1	invasive	USA	V	2002	1	SRR2981598
1623_SS62	CC1	invasive	USA	V	2002	1	SRR2981599
1624_SS63	CC1	invasive	USA	V	2002	1	SRR2981600
1625_SS64	CC1	invasive	USA	V	2002	1	SRR2981601
1626_SS65	CC1	invasive	USA	V	2002	1	SRR2981602
1627_SS66	CC1	invasive	USA	V	2002	1	SRR2981603
1628_SS67	CC1	invasive	USA	V	2002	1	SRR2981604
1629_SS68	CC1	invasive	USA	V	2003	1	SRR2981605
1630_SS69	CC1	invasive	USA	V	2003	1	SRR2981606
1632_SS71	CC1	invasive	USA	V	2003	1	SRR2981608
1633_SS72	CC1	invasive	USA	V	2003	1	SRR2981609
1634_SS74	CC1	invasive	USA	V	2004	1	SRR2981610
1635_SS75	CC1	invasive	USA	V	2004	1	SRR2981611
1636_SS76	CC1	invasive	USA	V	2004	1	SRR2981612
1637_SS77	CC1	invasive	USA	V	2004	1	SRR2981613
1638_SS78	CC1	invasive	USA	V	2004	1	SRR2981614
1639_SS79	CC1	invasive	USA	V	2004	153	SRR2981615
1640_SS80	CC1	invasive	USA	V	2004	1	SRR2981616
1641_SS81	CC1	invasive	USA	V	2004	1	SRR2981617
1642_SS82	CC1	invasive	USA	V	2004	1	SRR2981618
1643_SS83	CC1	invasive	USA	V	2004	1	SRR2981619
1644_SS84	CC1	invasive	USA	V	2005	1	SRR2981620
1645_SS85	CC1	invasive	USA	V	2005	1	SRR2981621
1646_SS86	CC1	invasive	USA	V	2005	1	SRR2981622
1647_SS87	CC1	invasive	USA	V	2005	1	SRR2981623
1648_SS88	CC1	invasive	USA	V	2005	1	SRR2981624
1649_SS89	CC1	invasive	USA	V	2005	1	SRR2981625
1650_SS92	CC1	invasive	USA	V	2005	1	SRR2981626
1651_SS93	CC1	invasive	USA	V	2005	1	SRR2981627
1652_SS94	CC1	invasive	USA	V	2005	1	SRR2981628
1653_SS95	CC1	invasive	USA	V	2005	1	SRR2981629
1654_SS96	CC1	invasive	USA	V	2005	1	SRR2981630
1655_SS97	CC1	invasive	USA	V	2005	1	SRR2981631
1656_SS98	CC1	invasive	USA	V	2005	1	SRR2981632
1657_NGBS003	CC1	invasive	Canada	III	2009	2	SRR2981633
1658_NGBS050	CC17	invasive	Canada	III	2010	17	SRR2451874
1659_NGBS069	CC17	invasive	Canada	III	2010	17	SRR2451875
1661_NGBS079	CC17	invasive	Canada	III	2010	17	SRR2451877
1662_NGBS082	CC17	invasive	Canada	III	2010	17	SRR2451879
1664_NGBS126	CC17	invasive	Canada	III	2010	17	SRR2451883
1665_NGBS128	CC17	invasive	Canada	III	2010	17	SRR2451884
1666_NGBS169	CC17	invasive	Canada	III	2010	17	SRR2451892

1667_NGBS186	CC17	invasive	Canada	III	2010	17	SRR2451893
1668_NGBS205	CC17	invasive	Canada	III	2011	17	SRR2451894
1669_NGBS220	CC17	invasive	Canada	III	2011	17	SRR2451895
1670_NGBS222	CC17	invasive	Canada	III	2010	17	SRR2451896
1671_NGBS238	CC17	invasive	Canada	III	2011	17	SRR2451897
1672_NGBS239	CC17	invasive	Canada	III	2011	17	SRR2451898
1673_NGBS250	CC17	invasive	Canada	III	2011	17	SRR2451899
1675_NGBS277	CC17	invasive	Canada	III	2010	17	SRR2451901
1676_NGBS282	CC17	invasive	Canada	III	2010	17	SRR2451902
1677_NGBS291	CC17	invasive	Canada	III	2010	17	SRR2451903
1678_NGBS296	CC17	invasive	Canada	III	2010	17	SRR2451904
1679_NGBS297	CC17	invasive	Canada	III	2010	17	SRR2451905
1680_NGBS299	CC17	invasive	Canada	III	2011	17	SRR2451906
1681_NGBS306	CC17	invasive	Canada	III	2011	17	SRR2451907
1682_NGBS312	CC17	invasive	Canada	III	2011	17	SRR2451908
1683_NGBS318	CC17	invasive	Canada	III	2011	290	SRR2451910
1684_NGBS327	CC17	invasive	Canada	III	2011	484	SRR2451911
1686_NGBS345	CC17	invasive	Canada	III	2011	874	SRR2451913
1687_NGBS356	CC17	invasive	Canada	III	2011	17	SRR2451914
1688_NGBS361	CC17	invasive	Canada	III	2011	17	SRR2451915
1689_NGBS362	CC17	invasive	Canada	III	2011	17	SRR2451916
1690_NGBS368	CC17	invasive	Canada	III	2011	17	SRR2451917
1692_NGBS374	CC17	invasive	Canada	III	2011	17	SRR2451919
1693_NGBS377	CC17	invasive	Canada	III	2011	17	SRR2451920
1695_NGBS398	CC17	invasive	Canada	III	2011	17	SRR2451922
1696_NGBS403	CC17	invasive	Canada	III	2011	17	SRR2451923
1698_NGBS421	CC17	invasive	Canada	III	2011	17	SRR2451925
1699_NGBS422	CC17	invasive	Canada	III	2011	17	SRR2451926
1700_NGBS431	CC17	invasive	Canada	III	2011	17	SRR2451927
1702_NGBS456	CC17	invasive	Canada	III	2011	17	SRR2451929
1703_NGBS464	CC17	invasive	Canada	III	2011	17	SRR2451930
1704_NGBS469	CC17	invasive	Canada	III	2011	17	SRR2451931
1705_NGBS470	CC17	invasive	Canada	III	2011	17	SRR2451932
1706_NGBS483	CC17	invasive	Canada	III	2011	17	SRR2451933
1707_NGBS485	CC17	invasive	Canada	III	2011	17	SRR2451934
1708_NGBS486	CC17	invasive	Canada	III	2011	17	SRR2451935
1709_NGBS500	CC17	invasive	Canada	III	2012	17	SRR2451936
1710_NGBS501	CC17	invasive	Canada	III	2012	17	SRR2451937
1711_NGBS502	CC17	invasive	Canada	III	2012	95	SRR2451938
1712_NGBS515	CC17	invasive	Canada	III	2012	17	SRR2451939
1714_NGBS531	CC17	invasive	Canada	III	2012	148	SRR2451941
1715_NGBS534	CC17	invasive	Canada	III	2012	17	SRR2451942
1716_NGBS551	CC17	invasive	Canada	III	2012	17	SRR2451943
1717_NGBS583	CC17	invasive	Canada	III	2012	17	SRR2451945
1718_NGBS593	CC17	invasive	Canada	III	2012	17	SRR2451946
1719_NGBS594	CC17	invasive	Canada	III	2012	17	SRR2451947
1720_NGBS596	CC17	invasive	Canada	III	2012	17	SRR2451948
1721_NGBS607	CC17	invasive	Canada	III	2012	17	SRR2451949
1722_NGBS608	CC17	invasive	Canada	III	2012	17	SRR2451950
1723_NGBS609	CC17	invasive	Canada	III	2012	17	SRR2451951
1724_NGBS613	CC17	invasive	Canada	III	2012	17	SRR2451952
1726_NGBS618	CC17	invasive	Canada	III	2012	17	SRR2451954
1727_NGBS622	CC17	invasive	Canada	III	2012	148	SRR2451955
1730_NGBS632	CC17	invasive	Canada	III	2012	17	SRR2451958
1731_NGBS636	CC17	invasive	Canada	III	2010	17	SRR2451959
1732_NGBS641	CC17	invasive	Canada	III	2010	17	SRR2451960
1733_NGBS644	CC17	invasive	Canada	III	2011	17	SRR2451961
1734_NGBS650	CC17	invasive	Canada	III	2011	17	SRR2451962
1735_NGBS44	CC1	invasive	Canada	V	2010	1	SRR1790791
1897_K59213_SRR3321197	CC1	invasive	Kenya	V	2012	1	
1898_K58722_SRR3321196	CC19	invasive	Kenya	III	2012	182	
1899_K56677_SRR3321195	CC1	invasive	Kenya	V	2012	1	

1900_K62205_SRR3321194	CC23	carrier	Kenya	Ia	2013	23	
1901_K61856_SRR3321193	CC23	carrier	Kenya	Ia	2013	23	
1902_K61593_SRR3321192	CC1	carrier	Kenya	V	2013	1	
1903_K61569_SRR3321191	CC10	carrier	Kenya	Ib	2013	10	
1904_K61520_SRR3321190	CC17	carrier	Kenya	III	2013	484	
1905_K61337_SRR3321189	CC10	carrier	Kenya	NT	2013	8	
1906_K61281_SRR3321188	CC1	carrier	Kenya	V	2013	1	
1907_K61004_SRR3321187	CC17	carrier	Kenya	III	2013	17	
1908_K60927_SRR3321186	CC23	carrier	Kenya	Ia	2013	23	
1909_K60926_SRR3321185	CC23	carrier	Kenya	Ia	2013	23	
1910_K60802_SRR3321184	CC19	carrier	Kenya	II	2013	28	
1911_K60691_SRR3321183	CC17	carrier	Kenya	III	2013	484	
1912_K59750_SRR3321182	CC17	carrier	Kenya	III	2012	484	
1913_K59732_SRR3321181	CC17	carrier	Kenya	III	2012	17	
1914_K59579_SRR3321180	CC10	carrier	Kenya	Ib	2012	8	
1915_K59519_SRR3321179	CC1	carrier	Kenya	V	2012	1	
1916_K59497_SRR3321178	CC17	carrier	Kenya	III	2012	17	
1917_K59496_SRR3321177	CC10	carrier	Kenya	Ib	2012	10	
1918_K59380_SRR3321176	CC19	carrier	Kenya	III	2012	182	
1919_K59319_SRR3321175	CC1	carrier	Kenya	V	2012	1	
1920_K59216_SRR3321174	CC17	carrier	Kenya	III	2012	17	
1921_K59112_SRR3321173	CC1	carrier	Kenya	NT	2012	1	
1922_K58953_SRR3321172	CC1	carrier	Kenya	V	2012	1	
1923_K58904_SRR3321171	CC1	carrier	Kenya	V	2012	1	
1924_K58847_SRR3321170	CC1	carrier	Kenya	V	2012	1	
1925_K58834_SRR3321169	CC23	carrier	Kenya	Ia	2012	23	
1926_K58784_SRR3321168	CC17	carrier	Kenya	III	2012	17	
1927_K58759_SRR3321167	CC17	carrier	Kenya	III	2012	484	
1928_K58245_SRR3321166	CC17	carrier	Kenya	III	2012	17	
1929_K58244_SRR3321165	CC10	carrier	Kenya	Ib	2012	10	
1930_K57971_SRR3321164	CC10	carrier	Kenya	II	2012	10	
1931_K57970_SRR3321163	CC10	carrier	Kenya	Ib	2012	8	
1932_K57879_SRR3321162	CC10	carrier	Kenya	Ib	2012	8	
1933_K57791_SRR3321161	CC10	carrier	Kenya	II	2012	10	
1934_K57354_SRR3321160	CC17	carrier	Kenya	III	2012	17	
1935_K57353_SRR3321159	CC17	carrier	Kenya	III	2012	17	
1936_K57172_SRR3321158	CC1	carrier	Kenya	VI	2012	14	
1937_K57063_SRR3321157	CC17	carrier	Kenya	III	2012	17	
1938_K56978_SRR3321156	CC19	carrier	Kenya	III	2012	182	
1939_K56941_SRR3321155	CC17	carrier	Kenya	III	2012	17	
1940_K56795_SRR3321154	CC23	carrier	Kenya	Ia	2012	23	
1941_K56485_SRR3321153	CC17	carrier	Kenya	III	2012	786	
1942_K56441_SRR3321152	CC23	carrier	Kenya	Ia	2012	23	
1943_K56376_SRR3321151	CC23	carrier	Kenya	Ia	2012	23	
1944_K62060_SRR3321150	CC23	carrier	Kenya	Ia		23	
1945_K61534_SRR3321149	CC10	carrier	Kenya	Ib		8	
1946_K60936_SRR3321148	CC1	carrier	Kenya	V		1	
1947_K59565_SRR3321147	CC17	carrier	Kenya	III		17	
1948_K59185_SRR3321146	CC17	carrier	Kenya	III		17	
1949_K59069_SRR3321145	CC17	carrier	Kenya	III		484	
1950_K58241_SRR3321144	CC19	carrier	Kenya	II		28	
1951_K57911_SRR3321143	CC17	carrier	Kenya	III		17	
1952_K57091_SRR3321142	CC19	carrier	Kenya	V		327	
1953_K56940_SRR3321141	CC19	carrier	Kenya	V		19	
1954_K56631_SRR3321140	CC1	carrier	Kenya	V		1	
1955_K56368_SRR3321139	CC10	carrier	Kenya	Ib		8	
1956_K56068_SRR3321138	CC19	carrier	Kenya	III		182	
1957_K52047_SRR3321137	CC23	carrier	Kenya	Ia		23	
1958_K51025_SRR3321136	CC1	carrier	Kenya	V		1	
1959_K49737_SRR3321135	CC23	carrier	Kenya	Ia		23	
1960_K31131_SRR3321134	CC17	carrier	Kenya	III		17	
1961_K62585_SRR3321133	CC23	carrier	Kenya	Ia	2013	23	

1962_K62584_SRR3321132	CC19	carrier	Kenya	V	2013	19	
1963_K62583_SRR3321131	CC17	carrier	Kenya	III	2013	17	
1964_K62582_SRR3321130	CC17	carrier	Kenya	III	2013	17	
1965_K62581_SRR3321129	CC10	carrier	Kenya	Ib	2013	10	
1966_K62580_SRR3321128	CC23	carrier	Kenya	Ia	2013	23	
1967_K62570_SRR3321127	CC23	carrier	Kenya	Ia	2013	23	
1968_K62569_SRR3321126	CC17	carrier	Kenya	III	2013	17	
1969_K62567_SRR3321125	CC23	carrier	Kenya	Ia	2013	23	
1970_K62558_SRR3321124	CC17	carrier	Kenya	IV	2013	291	
1971_K62557_SRR3321123	CC19	carrier	Kenya	III	2013	19	
1972_K62556_SRR3321122	CC23	carrier	Kenya	Ia	2013	23	
1973_K62554_SRR3321121	CC17	carrier	Kenya	III	2013	17	
1974_K62534_SRR3321120	CC23	carrier	Kenya	Ia	2013	23	
1975_K62476_SRR3321119	CC17	carrier	Kenya	III	2013	17	
1976_K62475_SRR3321118	CC17	carrier	Kenya	III	2013	484	
1977_K62474_SRR3321117	CC17	carrier	Kenya	III	2013	17	
1978_K62464_SRR3321116	CC17	carrier	Kenya	III	2013	17	
1979_K62463_SRR3321115	CC10	carrier	Kenya	Ib	2013	10	
1980_K62462_SRR3321114	CC10	carrier	Kenya	Ib	2013	10	
1981_K62461_SRR3321113	CC23	carrier	Kenya	Ia	2013	23	
1982_K62460_SRR3321112	CC1	carrier	Kenya	V	2013	1	
1983_K62459_SRR3321111	CC17	carrier	Kenya	III	2013	484	
1984_K62446_SRR3321110	CC23	carrier	Kenya	Ia	2013	23	
1985_K62445_SRR3321109	CC23	carrier	Kenya	Ia	2013	23	
1986_K62444_SRR3321108	CC23	carrier	Kenya	V	2013	23	
1987_K62419_SRR3321107	CC23	carrier	Kenya	Ia	2013	23	
1988_K62418_SRR3321106	CC17	carrier	Kenya	III	2013	17	
1989_K62393_SRR3321105	CC17	carrier	Kenya	III	2013	17	
1990_K62392_SRR3321104	CC19	carrier	Kenya	V	2013	328	
1991_K62350_SRR3321103	CC10	carrier	Kenya	Ib	2013	10	
1992_K62349_SRR3321102	CC17	carrier	Kenya	III	2013	484	
1993_K62348_SRR3321101	CC17	carrier	Kenya	III	2013	17	
1994_K62347_SRR3321100	CC19	carrier	Kenya	III	2013	19	
1995_K62313_SRR3321099	CC17	carrier	Kenya	III	2013	17	
1996_K62312_SRR3321098	CC23	carrier	Kenya	Ia	2013	23	
1997_K62311_SRR3321097	CC1	carrier	Kenya	V	2013	1	
1998_K62252_SRR3321096	CC19	carrier	Kenya	V	2013	19	
1999_K62251_SRR3321095	CC19	carrier	Kenya	III	2013	182	
2000_K62250_SRR3321094	CC17	carrier	Kenya	III	2013	484	
2001_K62249_SRR3321093	CC19	carrier	Kenya	V	2013	327	
2002_K62210_SRR3321092	CC1	carrier	Kenya	V	2013	1	
2003_K62209_SRR3321091	N/D	carrier	Kenya	Ib	2013	3	
2004_K62208_SRR3321090	CC1	carrier	Kenya	V	2013	1	
2005_K62207_SRR3321089	CC19	carrier	Kenya	V	2013	19	
2006_K62204_SRR3321088	CC23	carrier	Kenya	Ia	2013	23	
2007_K62170_SRR3321087	CC10	carrier	Kenya	Ib	2013	8	
2008_K62168_SRR3321086	CC17	carrier	Kenya	III	2013	17	
2009_K62167_SRR3321085	CC1	carrier	Kenya	V	2013	1	
2010_K62165_SRR3321084	CC1	carrier	Kenya	V	2013	1	
2011_K62164_SRR3321083	CC19	carrier	Kenya	III	2013	19	
2012_K62157_SRR3321082	N/D	carrier	Kenya	Ia	2013	103	
2013_K62156_SRR3321081	CC17	carrier	Kenya	III	2013	17	
2014_K62155_SRR3321080	CC1	carrier	Kenya	V	2013	1	
2015_K62154_SRR3321079	CC17	carrier	Kenya	III	2013	17	
2016_K62153_SRR3321078	CC17	carrier	Kenya	III	2013	17	
2017_K62152_SRR3321077	CC23	carrier	Kenya	Ia	2013	23	
2018_K62151_SRR3321076	CC10	carrier	Kenya	Ib	2013	8	
2019_K62145_SRR3321075	CC17	carrier	Kenya	III	2013	801	
2020_K62144_SRR3321074	CC1	carrier	Kenya	V	2013	1	
2021_K62143_SRR3321073	CC17	carrier	Kenya	III	2013	484	
2022_K62142_SRR3321072	CC17	carrier	Kenya	III	2013	484	
2023_K62111_SRR3321071	CC1	carrier	Kenya	V	2013	1	

2024_K62110_SRR3321070	CC17	carrier	Kenya	III	2013	17	
2025_K62080_SRR3321069	CC17	carrier	Kenya	III	2013	17	
2026_K62079_SRR3321068	CC17	carrier	Kenya	III	2013	17	
2027_K62064_SRR3321067	CC10	carrier	Kenya	II	2013	10	
2028_K62063_SRR3321066	CC19	carrier	Kenya	III	2013	182	
2029_K62062_SRR3321065	CC23	carrier	Kenya	Ia	2013	23	
2030_K62044_SRR3321064	CC1	carrier	Kenya	V	2013	1	
2031_K62043_SRR3321063	CC17	carrier	Kenya	III	2013	17	
2032_K62042_SRR3321062	CC17	carrier	Kenya	III	2013	17	
2033_K62032_SRR3321061	CC17	carrier	Kenya	III	2013	17	
2034_K62031_SRR3321060	CC10	carrier	Kenya	Ib	2013	10	
2035_K62030_SRR3321059	CC10	carrier	Kenya	Ib	2013	10	
2036_K62029_SRR3321058	CC19	carrier	Kenya	II	2013	28	
2037_K62026_SRR3321057	CC10	carrier	Kenya	Ib	2013	10	
2038_K62025_SRR3321056	CC17	carrier	Kenya	III	2013	17	
2039_K61994_SRR3321055	CC17	carrier	Kenya	III	2013	17	
2040_K61855_SRR3321054	CC19	carrier	Kenya	V	2013	327	
2041_K61845_SRR3321053	CC17	carrier	Kenya	III	2013	17	
2042_K61844_SRR3321052	CC17	carrier	Kenya	III	2013	17	
2043_K61843_SRR3321051	CC19	carrier	Kenya	III	2013	19	
2044_K61842_SRR3321050	CC10	carrier	Kenya	II	2013	10	
2045_K61841_SRR3321049	CC17	carrier	Kenya	III	2013	17	
2046_K61840_SRR3321048	CC17	carrier	Kenya	III	2013	17	
2047_K61832_SRR3321047	CC17	carrier	Kenya	III	2013	17	
2048_K61823_SRR3321046	CC19	carrier	Kenya	V	2013	19	
2049_K61781_SRR3321045	CC10	carrier	Kenya	Ib	2013	10	
2050_K61780_SRR3321044	CC23	carrier	Kenya	Ia	2013	23	
2051_K61773_SRR3321043	CC19	carrier	Kenya	II	2013	28	
2052_K61772_SRR3321042	CC23	carrier	Kenya	Ia	2013	23	
2053_K61771_SRR3321041	CC23	carrier	Kenya	Ia	2013	23	
2054_K61768_SRR3321040	CC23	carrier	Kenya	Ia	2013	23	
2055_K61767_SRR3321039	CC17	carrier	Kenya	III	2013	484	
2056_K61766_SRR3321038	CC10	carrier	Kenya	V	2013	12	
2057_K61765_SRR3321037	CC10	carrier	Kenya	V	2013	12	
2058_K61764_SRR3321036	CC10	carrier	Kenya	II	2013	10	
2059_K61763_SRR3321035	CC17	carrier	Kenya	III	2013	17	
2060_K61749_SRR3321034	CC19	carrier	Kenya	II	2013	28	
2061_K61748_SRR3321033	CC19	carrier	Kenya	III	2013	182	
2062_K61747_SRR3321032	CC23	carrier	Kenya	V	2013	23	
2063_K61746_SRR3321031	CC23	carrier	Kenya	Ia	2013	23	
2064_K61739_SRR3321030	CC23	carrier	Kenya	Ia	2013	23	
2065_K61733_SRR3321029	CC23	carrier	Kenya	Ia	2013	23	
2066_K61732_SRR3321028	CC1	carrier	Kenya	V	2013	1	
2067_K61731_SRR3321027	CC17	carrier	Kenya	III	2013	17	
2068_K61730_SRR3321026	CC23	carrier	Kenya	Ia	2013	23	
2069_K61729_SRR3321025	CC19	carrier	Kenya	V	2013	19	
2070_K61725_SRR3321024	CC19	carrier	Kenya	II	2013	28	
2071_K61704_SRR3321023	CC17	carrier	Kenya	III	2013	484	
2072_K61692_SRR3321022	CC19	carrier	Kenya	II	2013	28	
2073_K61691_SRR3321021	CC23	carrier	Kenya	Ia	2013	23	
2074_K61690_SRR3321020	CC1	carrier	Kenya	V	2013	1	
2075_K61689_SRR3321019	CC23	carrier	Kenya	Ia	2013	23	
2076_K61688_SRR3321018	CC17	carrier	Kenya	III	2013	109	
2077_K61685_SRR3321017	CC1	carrier	Kenya	V	2013	1	
2078_K61684_SRR3321016	CC19	carrier	Kenya	III	2013	182	
2079_K61683_SRR3321015	CC23	carrier	Kenya	Ia		23	
2080_K61670_SRR3321014	CC1	carrier	Kenya	V	2013	1	
2081_K61669_SRR3321013	CC23	carrier	Kenya	Ia	2013	23	
2082_K61668_SRR3321012	CC23	carrier	Kenya	Ia	2013	23	
2083_K61640_SRR3321011	CC17	carrier	Kenya	III	2013	17	
2084_K61639_SRR3321010	CC17	carrier	Kenya	III	2013	17	
2085_K61622_SRR3321009	CC10	carrier	Kenya	II	2013	10	

2086_K61620_SRR3321008	CC17	carrier	Kenya	III	2013	17	
2087_K61618_SRR3321007	CC10	carrier	Kenya	II	2013	10	
2088_K61611_SRR3321006	CC17	carrier	Kenya	III	2013	17	
2089_K61610_SRR3321005	CC23	carrier	Kenya	Ia	2013	23	
2090_K61608_SRR3321004	CC17	carrier	Kenya	III	2013	17	
2091_K61606_SRR3321003	CC19	carrier	Kenya	II	2013	28	
2092_K61594_SRR3321002	CC17	carrier	Kenya	III	2013	484	
2093_K61592_SRR3321001	CC10	carrier	Kenya	II	2013	10	
2094_K61591_SRR3321000	CC1	carrier	Kenya	II	2013	2	
2095_K61590_SRR3320999	CC17	carrier	Kenya	III	2013	17	
2096_K61589_SRR3320998	N/D	carrier	Kenya	Ia	2013	798	
2097_K61586_SRR3320997	CC17	carrier	Kenya	III	2013	484	
2098_K61572_SRR3320996	CC17	carrier	Kenya	III	2013	17	
2099_K61571_SRR3320995	CC23	carrier	Kenya	Ia	2013	23	
2100_K61533_SRR3320994	CC10	carrier	Kenya	Ib	2013	10	
2101_K61521_SRR3320993	CC17	carrier	Kenya	III	2013	17	
2102_K61516_SRR3320992	CC17	carrier	Kenya	III	2013	17	
2103_K61515_SRR3320991	CC10	carrier	Kenya	Ib	2013	8	
2104_K61514_SRR3320990	CC17	carrier	Kenya	III	2013	484	
2105_K61512_SRR3320989	CC19	carrier	Kenya	V	2013	327	
2106_K61491_SRR3320988	N/D	carrier	Kenya	Ib	2013		
2107_K61481_SRR3320987	CC19	carrier	Kenya	V	2013	327	
2108_K61465_SRR3320986	CC19	carrier	Kenya	III	2013	182	
2109_K61464_SRR3320985	CC10	carrier	Kenya	Ib	2013	797	
2110_K61463_SRR3320984	CC10	carrier	Kenya	II	2013	10	
2111_K61461_SRR3320983	CC17	carrier	Kenya	III	2013	484	
2112_K61460_SRR3320982	CC19	carrier	Kenya	III	2013	182	
2113_K61459_SRR3320981	CC23	carrier	Kenya	Ia	2013	23	
2114_K61457_SRR3320980	CC10	carrier	Kenya	Ib	2013	10	
2115_K61438_SRR3320979	CC23	carrier	Kenya	Ia	2013	23	
2116_K61437_SRR3320978	CC10	carrier	Kenya	Ib	2013	8	
2117_K61436_SRR3320977	CC10	carrier	Kenya	Ib	2013	10	
2118_K61435_SRR3320976	CC17	carrier	Kenya	III	2013	17	
2119_K61434_SRR3320975	CC1	carrier	Kenya	V	2013	1	
2120_K61432_SRR3320974	CC10	carrier	Kenya	Ib	2013	10	
2121_K61431_SRR3320973	CC17	carrier	Kenya	III	2013	790	
2122_K61428_SRR3320972	CC23	carrier	Kenya	Ia	2013	23	
2123_K61416_SRR3320971	CC10	carrier	Kenya	Ib	2013	10	
2124_K61415_SRR3320970	CC17	carrier	Kenya	III	2013	484	
2125_K61414_SRR3320969	CC23	carrier	Kenya	Ia	2013	23	
2126_K61407_SRR3320968	CC17	carrier	Kenya	III	2013	484	
2127_K61395_SRR3320967	CC23	carrier	Kenya	Ia	2013	23	
2128_K61394_SRR3320966	CC23	carrier	Kenya	Ia	2013	23	
2129_K61381_SRR3320965	CC1	carrier	Kenya	V	2013	1	
2130_K61379_SRR3320964	CC23	carrier	Kenya	Ia	2013	23	
2131_K61378_SRR3320963	CC10	carrier	Kenya	Ib	2013	10	
2132_K61363_SRR3320962	CC23	carrier	Kenya	Ia	2013	23	
2133_K61362_SRR3320961	CC17	carrier	Kenya	III	2013	17	
2134_K61361_SRR3320960	CC10	carrier	Kenya	Ib	2013	10	
2135_K61360_SRR3320959	CC1	carrier	Kenya	V	2013	796	
2136_K61359_SRR3320958	CC10	carrier	Kenya	II	2013	10	
2137_K61342_SRR3320957	CC19	carrier	Kenya	II	2013	28	
2138_K61341_SRR3320956	CC23	carrier	Kenya	V	2013	24	
2139_K61336_SRR3320955	CC17	carrier	Kenya	III	2013	484	
2140_K61335_SRR3320954	CC17	carrier	Kenya	III	2013	17	
2141_K61334_SRR3320953	CC23	carrier	Kenya	Ia	2013	23	
2142_K61331_SRR3320952	CC17	carrier	Kenya	III	2013	17	
2143_K61330_SRR3320951	CC10	carrier	Kenya	Ib	2013	8	
2144_K61329_SRR3320950	CC10	carrier	Kenya	Ib	2013	10	
2145_K61328_SRR3320949	CC23	carrier	Kenya	Ia	2013	23	
2146_K61308_SRR3320948	CC19	carrier	Kenya	V	2013	328	
2147_K61307_SRR3320947	CC1	carrier	Kenya	IV	2013	196	

2148_K61306_SRR3320946	CC10	carrier	Kenya	Ib	2013	8	
2149_K61305_SRR3320945	CC17	carrier	Kenya	III	2013	17	
2150_K61304_SRR3320944	CC23	carrier	Kenya	Ia	2013	23	
2151_K61282_SRR3320943	CC17	carrier	Kenya	III	2013	17	
2152_K61280_SRR3320942	CC17	carrier	Kenya	III	2013	484	
2153_K61279_SRR3320941	CC1	carrier	Kenya	V	2013	1	
2154_K61278_SRR3320940	CC23	carrier	Kenya	Ia	2013	23	
2155_K61276_SRR3320939	CC17	carrier	Kenya	III	2013	17	
2156_K61270_SRR3320938	CC19	carrier	Kenya	V	2013	19	
2157_K61269_SRR3320937	CC23	carrier	Kenya	Ia	2013	23	
2158_K61265_SRR3320936	CC10	carrier	Kenya	Ib	2013	8	
2159_K61263_SRR3320935	CC1	carrier	Kenya	V	2013	1	
2160_K61261_SRR3320934	CC1	carrier	Kenya	II	2013	2	
2161_K61259_SRR3320933	CC10	carrier	Kenya	Ib	2013	8	
2162_K61258_SRR3320932	CC23	carrier	Kenya	Ia	2013	23	
2163_K61256_SRR3320931	CC10	carrier	Kenya	Ib	2013	8	
2164_K61255_SRR3320930	CC19	carrier	Kenya	III	2013	182	
2165_K61252_SRR3320929	CC17	carrier	Kenya	III	2013	484	
2166_K61251_SRR3320928	CC17	carrier	Kenya	III	2013	17	
2167_K61250_SRR3320927	CC19	carrier	Kenya	III	2013	182	
2168_K61236_SRR3320926	CC1	carrier	Kenya	V	2013	1	
2169_K61235_SRR3320925	CC1	carrier	Kenya	II	2013	2	
2170_K61223_SRR3320924	CC23	carrier	Kenya	Ia	2013	23	
2171_K61219_SRR3320923	CC1	carrier	Kenya	II	2013	2	
2172_K61211_SRR3320922	CC23	carrier	Kenya	Ia	2013	23	
2173_K61196_SRR3320921	CC23	carrier	Kenya	Ia	2013	23	
2174_K61194_SRR3320920	CC23	carrier	Kenya	V	2013	23	
2175_K61193_SRR3320919	CC23	carrier	Kenya	Ia	2013	24	
2176_K61192_SRR3320918	CC19	carrier	Kenya	III	2013	182	
2177_K61191_SRR3320917	CC23	carrier	Kenya	Ia	2013	23	
2178_K61177_SRR3320916	CC17	carrier	Kenya	III	2013	484	
2179_K61175_SRR3320915	CC23	carrier	Kenya	Ia	2013	23	
2180_K61164_SRR3320914	CC17	carrier	Kenya	III	2013	484	
2181_K61163_SRR3320913	CC19	carrier	Kenya	V	2013	328	
2182_K61143_SRR3320912	CC1	carrier	Kenya	IV	2013	196	
2183_K61142_SRR3320911	CC1	carrier	Kenya	IV	2013	196	
2184_K61141_SRR3320910	CC10	carrier	Kenya	II	2013	10	
2185_K61138_SRR3320909	N/D	carrier	Kenya	V	2013		
2186_K61119_SRR3320908	CC19	carrier	Kenya	III	2013	182	
2187_K61118_SRR3320907	CC19	carrier	Kenya	III	2013	182	
2188_K61116_SRR3320906	CC19	carrier	Kenya	II	2013	28	
2189_K61115_SRR3320905	CC10	carrier	Kenya	Ib	2013	8	
2190_K61079_SRR3320904	CC17	carrier	Kenya	III	2013	17	
2191_K61078_SRR3320903	CC17	carrier	Kenya	III	2013	17	
2192_K61069_SRR3320902	CC23	carrier	Kenya	V	2013	223	
2193_K61068_SRR3320901	CC17	carrier	Kenya	III	2013	484	
2194_K61067_SRR3320900	CC19	carrier	Kenya	III	2013	182	
2195_K61066_SRR3320899	CC23	carrier	Kenya	V	2013	23	
2196_K61063_SRR3320898	CC17	carrier	Kenya	III	2013	17	
2197_K61062_SRR3320897	CC23	carrier	Kenya	Ia	2013	23	
2198_K61061_SRR3320896	CC1	carrier	Kenya	V	2013	1	
2199_K61060_SRR3320895	CC1	carrier	Kenya	V	2013	1	
2200_K61053_SRR3320894	CC17	carrier	Kenya	III	2013	484	
2201_K61035_SRR3320893	CC23	carrier	Kenya	Ia	2013	23	
2202_K61034_SRR3320892	CC23	carrier	Kenya	Ia	2013	23	
2203_K61018_SRR3320891	CC17	carrier	Kenya	III	2013	484	
2204_K61017_SRR3320890	CC1	carrier	Kenya	V	2013	794	
2205_K61016_SRR3320889	CC17	carrier	Kenya	III	2013	17	
2206_K61008_SRR3320888	CC10	carrier	Kenya	Ib	2013	8	
2207_K61006_SRR3320887	CC17	carrier	Kenya	III	2013	17	
2208_K61005_SRR3320886	CC23	carrier	Kenya	Ia	2013	23	
2209_K61000_SRR3320885	CC1	carrier	Kenya	II	2013	2	

2210_K60999_SRR3320884	CC1	carrier	Kenya	V	2013	1	
2211_K60994_SRR3320883	CC23	carrier	Kenya	Ia	2013	23	
2212_K60992_SRR3320882	CC23	carrier	Kenya	Ia	2013	23	
2213_K60961_SRR3320881	CC19	carrier	Kenya	II	2013	28	
2214_K60957_SRR3320880	CC17	carrier	Kenya	III	2013	17	
2215_K60940_SRR3320879	CC19	carrier	Kenya	III		19	
2216_K60939_SRR3320878	CC17	carrier	Kenya	III	2013	17	
2217_K60937_SRR3320877	CC10	carrier	Kenya	Ib	2013	10	
2218_K60929_SRR3320876	CC23	carrier	Kenya	Ia	2013	802	
2219_K60928_SRR3320875	CC10	carrier	Kenya	NT	2013	10	
2220_K60905_SRR3320874	CC19	carrier	Kenya	III	2013	182	
2221_K60903_SRR3320873	CC10	carrier	Kenya	II	2013	10	
2222_K60902_SRR3320872	CC19	carrier	Kenya	II	2013	28	
2223_K60901_SRR3320871	N/D	carrier	Kenya	II	2013		
2224_K60888_SRR3320870	CC23	carrier	Kenya	Ia	2013	23	
2225_K60887_SRR3320869	CC17	carrier	Kenya	III	2013	17	
2226_K60886_SRR3320868	CC23	carrier	Kenya	Ia	2013	23	
2227_K60885_SRR3320867	CC17	carrier	Kenya	III	2013	17	
2228_K60881_SRR3320866	CC10	carrier	Kenya	Ib	2013	8	
2229_K60880_SRR3320865	CC10	carrier	Kenya	Ib	2013	8	
2230_K60878_SRR3320864	CC1	carrier	Kenya	V	2013	1	
2231_K60877_SRR3320863	CC1	carrier	Kenya	V	2013	799	
2232_K60876_SRR3320862	CC23	carrier	Kenya	Ia	2013	23	
2233_K60871_SRR3320861	CC23	carrier	Kenya	Ia	2013	23	
2234_K60870_SRR3320860	CC1	carrier	Kenya	IV	2013	196	
2235_K60844_SRR3320859	CC23	carrier	Kenya	Ia	2013	23	
2236_K60843_SRR3320858	CC17	carrier	Kenya	III	2013	800	
2237_K60831_SRR3320857	CC17	carrier	Kenya	III	2013	17	
2238_K60830_SRR3320856	CC17	carrier	Kenya	III	2013	17	
2239_K60829_SRR3320855	CC10	carrier	Kenya	Ib	2013	8	
2240_K60812_SRR3320854	CC17	carrier	Kenya	III	2013	17	
2241_K60811_SRR3320853	CC19	carrier	Kenya	III	2013	19	
2242_K60801_SRR3320852	CC10	carrier	Kenya	Ib	2013	10	
2243_K60800_SRR3320851	CC19	carrier	Kenya	II	2013	28	
2244_K60799_SRR3320850	CC19	carrier	Kenya	III	2013	182	
2245_K60797_SRR3320849	CC17	carrier	Kenya	III	2013	17	
2246_K60796_SRR3320848	CC19	carrier	Kenya	II	2013	28	
2247_K60794_SRR3320847	CC23	carrier	Kenya	Ia	2013	23	
2248_K60793_SRR3320846	CC17	carrier	Kenya	III	2013	484	
2249_K60792_SRR3320845	N/D	carrier	Kenya	III	2013		
2250_K60790_SRR3320844	CC17	carrier	Kenya	III	2013	17	
2251_K60789_SRR3320843	CC19	carrier	Kenya	V	2013	327	
2252_K60788_SRR3320842	CC17	carrier	Kenya	III	2013	17	
2253_K60785_SRR3320841	CC10	carrier	Kenya	Ib	2013	8	
2254_K60756_SRR3320840	CC17	carrier	Kenya	III	2013	17	
2255_K60721_SRR3320839	CC19	carrier	Kenya	III	2013	182	
2256_K60720_SRR3320838	CC17	carrier	Kenya	III	2013	17	
2257_K60719_SRR3320837	CC17	carrier	Kenya	III	2013	17	
2258_K60715_SRR3320836	CC17	carrier	Kenya	III	2013	484	
2259_K60714_SRR3320835	CC23	carrier	Kenya	Ia	2013	23	
2260_K60693_SRR3320834	CC1	carrier	Kenya	V	2013	799	
2261_K60690_SRR3320833	CC1	carrier	Kenya	V	2013	1	
2262_K60689_SRR3320832	CC23	carrier	Kenya	V	2013	24	
2263_K60688_SRR3320831	CC10	carrier	Kenya	II	2013	10	
2264_K60670_SRR3320830	CC17	carrier	Kenya	III	2013	17	
2265_K60668_SRR3320829	CC10	carrier	Kenya	Ib	2013	10	
2266_K60666_SRR3320828	CC1	carrier	Kenya	VII	2013	1	
2267_K60664_SRR3320827	CC23	carrier	Kenya	Ia	2013	23	
2268_K60663_SRR3320826	CC17	carrier	Kenya	III	2013	17	
2269_K60658_SRR3320825	CC23	carrier	Kenya	Ia	2013	23	
2270_K60657_SRR3320824	CC1	carrier	Kenya	V	2013	796	
2271_K60577_SRR3320823	CC23	carrier	Kenya	V	2013	23	

2272_K60576_SRR3320822	CC10	carrier	Kenya	Ib	2013	8	
2273_K60575_SRR3320821	CC1	carrier	Kenya	IV	2013	196	
2274_K60574_SRR3320820	CC17	carrier	Kenya	III	2013	17	
2275_K60573_SRR3320819	CC23	carrier	Kenya	III	2013	55	
2276_K60572_SRR3320818	CC19	carrier	Kenya	III	2013	182	
2277_K60566_SRR3320817	CC17	carrier	Kenya	III	2013	17	
2278_K60565_SRR3320816	CC17	carrier	Kenya	III	2013	484	
2279_K60564_SRR3320815	CC17	carrier	Kenya	III	2013	17	
2280_K60516_SRR3320814	CC10	carrier	Kenya	Ib	2013	8	
2281_K60508_SRR3320813	CC10	carrier	Kenya	Ib	2013	8	
2282_K60442_SRR3320812	CC10	carrier	Kenya	Ib	2013	8	
2283_K60434_SRR3320811	CC23	carrier	Kenya	Ia	2013	23	
2284_K60433_SRR3320810	CC19	carrier	Kenya	III	2013	182	
2285_K60432_SRR3320809	CC17	carrier	Kenya	III	2013	17	
2286_K60428_SRR3320808	CC19	carrier	Kenya	V	2013	793	
2287_K60427_SRR3320807	CC19	carrier	Kenya	V	2013	793	
2288_K60426_SRR3320806	CC19	carrier	Kenya	V	2013	328	
2289_K60291_SRR3320805	CC17	carrier	Kenya	III	2013	17	
2290_K60290_SRR3320804	CC19	carrier	Kenya	III	2013	19	
2291_K60028_SRR3320803	CC19	carrier	Kenya	V	2012	327	
2292_K59962_SRR3320802	CC19	carrier	Kenya	III	2012	19	
2293_K59927_SRR3320801	CC19	carrier	Kenya	III	2012	19	
2294_K59917_SRR3320800	CC10	carrier	Kenya	Ib	2012	8	
2295_K59881_SRR3320799	CC23	carrier	Kenya	Ia	2012	23	
2296_K59813_SRR3320798	CC10	carrier	Kenya	Ib	2012	10	
2297_K59793_SRR3320797	CC19	carrier	Kenya	III	2012	182	
2298_K59792_SRR3320796	CC19	carrier	Kenya	V	2012	327	
2299_K59791_SRR3320795	CC17	carrier	Kenya	III	2012	17	
2300_K59790_SRR3320794	CC1	carrier	Kenya	V	2012	1	
2301_K59789_SRR3320793	CC10	carrier	Kenya	II	2012	10	
2302_K59778_SRR3320792	CC17	carrier	Kenya	III	2012	484	
2303_K59777_SRR3320791	CC23	carrier	Kenya	Ia	2012	23	
2304_K59776_SRR3320790	CC19	carrier	Kenya	II	2012	28	
2305_K59775_SRR3320789	CC19	carrier	Kenya	III	2012	182	
2306_K59774_SRR3320788	CC10	carrier	Kenya	Ib	2012	8	
2307_K59773_SRR3320787	CC10	carrier	Kenya	Ib	2012	8	
2308_K59772_SRR3320786	CC17	carrier	Kenya	III	2012	17	
2309_K59754_SRR3320785	CC19	carrier	Kenya	III	2012	182	
2310_K59753_SRR3320784	CC17	carrier	Kenya	III	2012	17	
2311_K59752_SRR3320783	CC19	carrier	Kenya	II	2012	28	
2312_K59749_SRR3320782	CC17	carrier	Kenya	III	2012	484	
2313_K59748_SRR3320781	CC1	carrier	Kenya	V	2012	1	
2314_K59747_SRR3320780	CC17	carrier	Kenya	III	2012	17	
2315_K59746_SRR3320779	CC23	carrier	Kenya	Ia	2012	23	
2316_K59733_SRR3320778	CC17	carrier	Kenya	III	2012	17	
2317_K59731_SRR3320777	CC17	carrier	Kenya	III	2012	17	
2318_K59730_SRR3320776	CC23	carrier	Kenya	Ia	2012	23	
2319_K59729_SRR3320775	CC17	carrier	Kenya	III	2012	484	
2320_K59712_SRR3320774	CC17	carrier	Kenya	III	2012	795	
2321_K59711_SRR3320773	CC17	carrier	Kenya	III	2012	17	
2322_K59710_SRR3320772	CC17	carrier	Kenya	III	2012	17	
2323_K59673_SRR3320771	CC17	carrier	Kenya	III	2012	484	
2324_K59648_SRR3320770	CC17	carrier	Kenya	III	2012	17	
2325_K59646_SRR3320769	CC23	carrier	Kenya	Ia	2012	23	
2326_K59645_SRR3320768	CC17	carrier	Kenya	III	2012	17	
2327_K59605_SRR3320767	CC17	carrier	Kenya	III	2012	484	
2328_K59604_SRR3320766	CC19	carrier	Kenya	III	2012	182	
2329_K59594_SRR3320765	CC1	carrier	Kenya	V	2012	1	
2330_K59593_SRR3320764	CC19	carrier	Kenya	III	2012	182	
2331_K59591_SRR3320763	CC10	carrier	Kenya	Ib	2012	10	
2332_K59590_SRR3320762	CC17	carrier	Kenya	III	2012	17	
2333_K59589_SRR3320761	CC23	carrier	Kenya	Ia	2012	23	

2334_K59580_SRR3320760	CC10	carrier	Kenya	Ib	2012	8	
2335_K59567_SRR3320759	CC10	carrier	Kenya	Ib	2012	10	
2336_K59566_SRR3320758	CC17	carrier	Kenya	III	2012	801	
2337_K59564_SRR3320757	CC17	carrier	Kenya	III	2012	17	
2338_K59528_SRR3320756	CC23	carrier	Kenya	Ia	2012	23	
2339_K59527_SRR3320755	CC23	carrier	Kenya	V	2012	23	
2340_K59526_SRR3320754	CC1	carrier	Kenya	V	2012	1	
2341_K59524_SRR3320753	CC1	carrier	Kenya	V	2012	1	
2342_K59522_SRR3320752	CC17	carrier	Kenya	III	2012	484	
2343_K59518_SRR3320751	CC1	carrier	Kenya	V	2012	1	
2344_K59501_SRR3320750	CC23	carrier	Kenya	V	2012	23	
2345_K59500_SRR3320749	CC10	carrier	Kenya	Ib	2012	10	
2346_K59499_SRR3320748	CC23	carrier	Kenya	Ia	2012	23	
2347_K59498_SRR3320747	CC17	carrier	Kenya	III	2012	484	
2348_K59495_SRR3320746	CC10	carrier	Kenya	Ib	2012	10	
2349_K59494_SRR3320745	CC1	carrier	Kenya	V	2012	1	
2350_K59463_SRR3320744	CC1	carrier	Kenya	V	2012	1	
2351_K59441_SRR3320743	CC23	carrier	Kenya	Ia	2012	23	
2352_K59440_SRR3320742	CC23	carrier	Kenya	Ia	2012	23	
2353_K59439_SRR3320741	CC17	carrier	Kenya	III	2012	17	
2354_K59426_SRR3320740	CC17	carrier	Kenya	III	2012	17	
2355_K59425_SRR3320739	CC17	carrier	Kenya	III	2012	17	
2356_K59423_SRR3320738	CC17	carrier	Kenya	III	2012	17	
2357_K59411_SRR3320737	CC1	carrier	Kenya	V	2012	1	
2358_K59410_SRR3320736	CC17	carrier	Kenya	III	2012	484	
2359_K59354_SRR3320735	CC1	carrier	Kenya	Ia	2012	1	
2360_K59352_SRR3320734	CC19	carrier	Kenya	III	2012	182	
2361_K59292_SRR3320733	CC23	carrier	Kenya	Ia	2012	23	
2362_K59290_SRR3320732	CC19	carrier	Kenya	III	2012	182	
2363_K59288_SRR3320731	CC23	carrier	Kenya	Ia	2012	23	
2364_K59245_SRR3320730	CC17	carrier	Kenya	III	2012	484	
2365_K59230_SRR3320729	CC19	carrier	Kenya	V	2012	328	
2366_K59219_SRR3320728	CC19	carrier	Kenya	III	2012	182	
2367_K59218_SRR3320727	CC19	carrier	Kenya	III	2012	182	
2368_K59217_SRR3320726	CC10	carrier	Kenya	Ib	2012	10	
2369_K59200_SRR3320725	CC17	carrier	Kenya	III	2012	17	
2370_K59199_SRR3320724	CC19	carrier	Kenya	II	2012	28	
2371_K59184_SRR3320723	CC1	carrier	Kenya	V	2012	796	
2372_K59183_SRR3320722	CC17	carrier	Kenya	III	2012	17	
2373_K59182_SRR3320721	CC1	carrier	Kenya	V	2012	1	
2374_K59181_SRR3320720	CC10	carrier	Kenya	Ib	2012	10	
2375_K59167_SRR3320719	CC23	carrier	Kenya	Ia	2012	23	
2376_K59154_SRR3320718	CC19	carrier	Kenya	III	2012	182	
2377_K59148_SRR3320717	CC23	carrier	Kenya	Ia	2012	498	
2378_K59126_SRR3320716	CC17	carrier	Kenya	III	2012	17	
2379_K59113_SRR3320715	CC17	carrier	Kenya	III	2012	17	
2380_K59097_SRR3320714	CC17	carrier	Kenya	III	2012	17	
2381_K59095_SRR3320713	CC17	carrier	Kenya	III	2012	17	
2382_K59094_SRR3320712	CC19	carrier	Kenya	III	2012	182	
2383_K59086_SRR3320711	CC23	carrier	Kenya	Ia	2012	23	
2384_K59071_SRR3320710	CC10	carrier	Kenya	II	2012	10	
2385_K59070_SRR3320709	CC1	carrier	Kenya	V	2012	1	
2386_K59068_SRR3320708	CC1	carrier	Kenya	VII	2012	167	
2387_K59067_SRR3320707	CC1	carrier	Kenya	V	2012	1	
2388_K58954_SRR3320706	CC1	carrier	Kenya	V	2012	1	
2389_K58915_SRR3320705	CC19	carrier	Kenya	II	2012	28	
2390_K58906_SRR3320704	CC10	carrier	Kenya	II	2012	10	
2391_K58905_SRR3320703	CC1	carrier	Kenya	V	2012	1	
2392_K58884_SRR3320702	CC19	carrier	Kenya	V	2012	19	
2393_K58864_SRR3320701	CC1	carrier	Kenya	V	2012	1	
2394_K58833_SRR3320700	CC23	carrier	Kenya	Ia	2012	23	
2395_K58820_SRR3320699	CC23	carrier	Kenya	Ia	2012	23	

2396_K58806_SRR3320698	CC10	carrier	Kenya	II	2012	10	
2397_K58805_SRR3320697	CC17	carrier	Kenya	III	2012	17	
2398_K58801_SRR3320696	CC23	carrier	Kenya	Ia	2012	23	
2399_K58795_SRR3320695	CC17	carrier	Kenya	III	2012	17	
2400_K58773_SRR3320694	CC1	carrier	Kenya	V	2012	1	
2401_K58771_SRR3320693	CC23	carrier	Kenya	V	2012	23	
2402_K58760_SRR3320692	CC17	carrier	Kenya	III	2012	17	
2403_K58758_SRR3320691	CC17	carrier	Kenya	III	2012	484	
2404_K58737_SRR3320690	CC19	carrier	Kenya	III	2012	182	
2405_K58736_SRR3320689	CC10	carrier	Kenya	II	2012	10	
2406_K58734_SRR3320688	CC19	carrier	Kenya	III	2012	19	
2407_K58711_SRR3320687	CC17	carrier	Kenya	III	2012	484	
2408_K58710_SRR3320686	CC10	carrier	Kenya	Ib	2012	10	
2409_K58694_SRR3320685	CC17	carrier	Kenya	III	2012	17	
2410_K58690_SRR3320684	CC10	carrier	Kenya	Ib	2012	10	
2411_K58634_SRR3320683	CC17	carrier	Kenya	III	2012	17	
2412_K58631_SRR3320682	CC23	carrier	Kenya	Ia	2012	23	
2413_K58613_SRR3320681	CC1	carrier	Kenya	V	2012	796	
2414_K58611_SRR3320680	CC23	carrier	Kenya	Ia	2012	23	
2415_K58610_SRR3320679	CC23	carrier	Kenya	Ia	2012	23	
2416_K58593_SRR3320678	CC1	carrier	Kenya	IV	2012	196	
2417_K58592_SRR3320677	CC23	carrier	Kenya	Ia	2012	23	
2418_K58591_SRR3320676	CC19	carrier	Kenya	III	2012	182	
2419_K58574_SRR3320675	CC17	carrier	Kenya	III	2012	484	
2420_K58551_SRR3320674	CC17	carrier	Kenya	III	2012	484	
2421_K58495_SRR3320673	CC19	carrier	Kenya	III	2012	182	
2422_K58493_SRR3320672	CC17	carrier	Kenya	III	2012	17	
2423_K58491_SRR3320671	CC17	carrier	Kenya	III	2012	17	
2424_K58489_SRR3320670	CC10	carrier	Kenya	II	2012	10	
2425_K58460_SRR3320669	CC23	carrier	Kenya	Ia	2012	23	
2426_K58435_SRR3320668	CC17	carrier	Kenya	III	2012	17	
2427_K58418_SRR3320667	CC10	carrier	Kenya	Ib	2012	10	
2428_K58417_SRR3320666	CC23	carrier	Kenya	Ia	2012	23	
2429_K58331_SRR3320665	CC19	carrier	Kenya	III	2012	182	
2430_K58317_SRR3320664	CC23	carrier	Kenya	Ia	2012	23	
2431_K58316_SRR3320663	CC10	carrier	Kenya	II	2012	10	
2432_K58308_SRR3320662	CC1	carrier	Kenya	V	2012	1	
2433_K58307_SRR3320661	CC10	carrier	Kenya	Ib	2012	8	
2434_K58306_SRR3320660	CC10	carrier	Kenya	Ib	2012	8	
2435_K58274_SRR3320659	CC10	carrier	Kenya	Ib	2012	8	
2436_K58243_SRR3320658	CC17	carrier	Kenya	III	2012	17	
2437_K58242_SRR3320657	CC10	carrier	Kenya	Ib	2012	10	
2438_K58211_SRR3320656	CC17	carrier	Kenya	III	2012	801	
2439_K58185_SRR3320655	CC10	carrier	Kenya	Ib	2012	8	
2440_K58137_SRR3320654	CC1	carrier	Kenya	V	2012	1	
2441_K58136_SRR3320653	CC17	carrier	Kenya	III	2012	17	
2442_K58105_SRR3320652	CC10	carrier	Kenya	Ib	2012	8	
2443_K58104_SRR3320651	CC17	carrier	Kenya	III	2012	17	
2444_K58057_SRR3320650	CC23	carrier	Kenya	Ia	2012	23	
2445_K58056_SRR3320649	CC1	carrier	Kenya	V	2012	1	
2446_K57992_SRR3320648	CC10	carrier	Kenya	II	2012	10	
2447_K57976_SRR3320647	CC19	carrier	Kenya	III	2012	182	
2448_K57973_SRR3320646	CC10	carrier	Kenya	Ib	2012	8	
2449_K57972_SRR3320645	CC1	carrier	Kenya	V	2012	796	
2450_K57969_SRR3320644	CC23	carrier	Kenya	Ia	2012	23	
2451_K57965_SRR3320643	CC17	carrier	Kenya	III	2012	17	
2452_K57964_SRR3320642	CC23	carrier	Kenya	Ia	2012	23	
2453_K57912_SRR3320641	CC19	carrier	Kenya	II	2012	28	
2454_K57910_SRR3320640	CC17	carrier	Kenya	III	2012	17	
2455_K57878_SRR3320639	CC23	carrier	Kenya	Ia	2012	23	
2456_K57877_SRR3320638	CC10	carrier	Kenya	Ib	2012	8	
2457_K57843_SRR3320637	CC17	carrier	Kenya	III	2012	17	

2458_K57809_SRR3320636	N/D	carrier	Kenya	III	2012	791	
2459_K57808_SRR3320635	N/D	carrier	Kenya	V	2012		
2460_K57807_SRR3320634	CC1	carrier	Kenya	VII	2012	1	
2461_K57806_SRR3320633	CC17	carrier	Kenya	III	2012	17	
2462_K57805_SRR3320632	CC1	carrier	Kenya	V	2012	1	
2463_K57804_SRR3320631	CC10	carrier	Kenya	Ib	2012	8	
2464_K57803_SRR3320630	CC23	carrier	Kenya	Ia	2012	23	
2465_K57788_SRR3320629	CC10	carrier	Kenya	II	2012	10	
2466_K57787_SRR3320628	CC10	carrier	Kenya	Ib	2012	10	
2467_K57786_SRR3320627	CC23	carrier	Kenya	Ia	2012	23	
2468_K57751_SRR3320626	CC17	carrier	Kenya	III	2012	484	
2469_K57749_SRR3320625	CC17	carrier	Kenya	III	2012	484	
2470_K57643_SRR3320624	CC1	carrier	Kenya	V	2012	1	
2471_K57571_SRR3320623	CC10	carrier	Kenya	II	2012	10	
2472_K57570_SRR3320622	CC19	carrier	Kenya	V	2012	327	
2473_K57546_SRR3320621	CC10	carrier	Kenya	II	2012	10	
2474_K57545_SRR3320620	CC1	carrier	Kenya	V	2012	1	
2475_K57543_SRR3320619	CC17	carrier	Kenya	III	2012	17	
2476_K57541_SRR3320618	CC19	carrier	Kenya	III	2012	182	
2477_K57540_SRR3320617	CC19	carrier	Kenya	II	2012	28	
2478_K57539_SRR3320616	CC19	carrier	Kenya	V	2012	327	
2479_K57538_SRR3320615	CC17	carrier	Kenya	III	2012	484	
2480_K57525_SRR3320614	CC1	carrier	Kenya	V	2012	1	
2481_K57524_SRR3320613	CC19	carrier	Kenya	III	2012	182	
2482_K57515_SRR3320612	CC1	carrier	Kenya	V	2012	796	
2483_K57514_SRR3320611	CC23	carrier	Kenya	Ia	2012	23	
2484_K57513_SRR3320610	CC17	carrier	Kenya	III	2012	17	
2485_K57479_SRR3320609	CC19	carrier	Kenya	III	2012	182	
2486_K57478_SRR3320608	CC23	carrier	Kenya	Ia	2012	23	
2487_K57477_SRR3320607	CC17	carrier	Kenya	III	2012	17	
2488_K57403_SRR3320606	CC10	carrier	Kenya	Ib	2012	8	
2489_K57402_SRR3320605	CC17	carrier	Kenya	III	2012	484	
2490_K57401_SRR3320604	CC19	carrier	Kenya	III	2012	182	
2491_K57400_SRR3320603	CC10	carrier	Kenya	Ib	2012	8	
2492_K57355_SRR3320602	CC10	carrier	Kenya	II	2012	10	
2493_K57352_SRR3320601	CC19	carrier	Kenya	II	2012	28	
2494_K57351_SRR3320600	CC17	carrier	Kenya	III	2012	17	
2495_K57350_SRR3320599	CC17	carrier	Kenya	III	2012	17	
2496_K57349_SRR3320598	CC17	carrier	Kenya	III	2012	17	
2497_K57348_SRR3320597	CC1	carrier	Kenya	IV	2012	196	
2498_K57347_SRR3320596	CC23	carrier	Kenya	V	2012	223	
2499_K57346_SRR3320595	CC10	carrier	Kenya	II	2012	10	
2500_K57335_SRR3320594	CC17	carrier	Kenya	III	2012	17	
2501_K57334_SRR3320593	CC23	carrier	Kenya	Ia	2012	23	
2502_K57199_SRR3320592	CC23	carrier	Kenya	V	2012	23	
2503_K57198_SRR3320591	CC23	carrier	Kenya	Ia	2012	23	
2504_K57174_SRR3320590	CC23	carrier	Kenya	Ia	2012	23	
2505_K57173_SRR3320589	CC23	carrier	Kenya	Ia	2012	23	
2506_K57171_SRR3320588	CC23	carrier	Kenya	Ia	2012	23	
2507_K57149_SRR3320587	CC23	carrier	Kenya	Ia	2012	144	
2508_K57135_SRR3320586	CC23	carrier	Kenya	Ia	2012	23	
2509_K57124_SRR3320585	CC1	carrier	Kenya	IV	2012	196	
2510_K57093_SRR3320584	CC23	carrier	Kenya	Ia	2012	23	
2511_K57092_SRR3320583	CC23	carrier	Kenya	Ia	2012	23	
2512_K57090_SRR3320582	CC23	carrier	Kenya	Ia	2012	23	
2513_K57089_SRR3320581	CC17	carrier	Kenya	III	2012	17	
2514_K57069_SRR3320580	CC19	carrier	Kenya	V	2012	327	
2515_K57068_SRR3320579	CC19	carrier	Kenya	III	2012	182	
2516_K57066_SRR3320578	CC19	carrier	Kenya	II	2012	28	
2517_K57064_SRR3320577	CC23	carrier	Kenya	Ia	2012	23	
2518_K57062_SRR3320576	CC10	carrier	Kenya	Ib	2012	10	
2519_K57056_SRR3320575	CC10	carrier	Kenya	II	2012	10	

2520_K57050_SRR3320574	CC1	carrier	Kenya	IV	2012	196	
2521_K57049_SRR3320573	CC17	carrier	Kenya	III	2012	17	
2522_K57015_SRR3320572	CC23	carrier	Kenya	Ia	2012	23	
2523_K56996_SRR3320571	CC1	carrier	Kenya	V	2012	1	
2524_K56995_SRR3320570	CC17	carrier	Kenya	III	2012	484	
2525_K56992_SRR3320569	CC10	carrier	Kenya	Ib	2012	8	
2526_K56991_SRR3320568	CC17	carrier	Kenya	III	2012	484	
2527_K56989_SRR3320567	CC17	carrier	Kenya	III	2012	484	
2528_K56988_SRR3320566	CC17	carrier	Kenya	III	2012	17	
2529_K56981_SRR3320565	CC17	carrier	Kenya	III	2012	790	
2530_K56957_SRR3320564	CC23	carrier	Kenya	Ia	2012	23	
2531_K56949_SRR3320563	CC17	carrier	Kenya	III	2012	17	
2532_K56943_SRR3320562	CC19	carrier	Kenya	V	2012	327	
2533_K56917_SRR3320561	N/D	carrier	Kenya	Ia	2012	486	
2534_K56914_SRR3320560	CC23	carrier	Kenya	Ia	2012	23	
2535_K56913_SRR3320559	CC23	carrier	Kenya	Ia	2012	23	
2536_K56889_SRR3320558	CC17	carrier	Kenya	III	2012	17	
2537_K56872_SRR3320557	CC10	carrier	Kenya	NT	2012	8	
2538_K56866_SRR3320556	CC17	carrier	Kenya	III	2012	484	
2539_K56857_SRR3320555	CC17	carrier	Kenya	III	2012	17	
2540_K56814_SRR3320554	CC17	carrier	Kenya	III	2012	484	
2541_K56813_SRR3320553	CC19	carrier	Kenya	V	2012	327	
2542_K56810_SRR3320552	CC23	carrier	Kenya	Ia	2012	23	
2543_K56809_SRR3320551	CC17	carrier	Kenya	III	2012	17	
2544_K56802_SRR3320550	CC10	carrier	Kenya	Ib	2012	8	
2545_K56800_SRR3320549	CC23	carrier	Kenya	Ia	2012	23	
2546_K56799_SRR3320548	CC19	carrier	Kenya	V	2012	19	
2547_K56798_SRR3320547	CC23	carrier	Kenya	Ia	2012	23	
2548_K56797_SRR3320546	CC19	carrier	Kenya	III	2012	182	
2549_K56796_SRR3320545	CC17	carrier	Kenya	III	2012	17	
2550_K56794_SRR3320544	CC17	carrier	Kenya	III	2012	17	
2551_K56781_SRR3320543	CC23	carrier	Kenya	Ia	2012	23	
2552_K56778_SRR3320542	CC23	carrier	Kenya	Ia	2012	23	
2553_K56777_SRR3320541	CC23	carrier	Kenya	Ia	2012	23	
2554_K56776_SRR3320540	CC23	carrier	Kenya	Ia	2012	23	
2555_K56758_SRR3320539	CC23	carrier	Kenya	Ia	2012	23	
2556_K56745_SRR3320538	CC23	carrier	Kenya	Ia	2012	23	
2557_K56722_SRR3320537	CC19	carrier	Kenya	III	2012	182	
2558_K56721_SRR3320536	CC17	carrier	Kenya	III	2012	484	
2559_K56719_SRR3320535	CC10	carrier	Kenya	Ib	2012	8	
2560_K56718_SRR3320534	CC23	carrier	Kenya	Ia	2012	23	
2561_K56704_SRR3320533	CC19	carrier	Kenya	III	2012	182	
2562_K56702_SRR3320532	CC23	carrier	Kenya	Ia	2012	23	
2563_K56689_SRR3320531	CC23	carrier	Kenya	Ia	2012	23	
2564_K56687_SRR3320530	CC23	carrier	Kenya	Ia	2012	23	
2565_K56685_SRR3320529	CC10	carrier	Kenya	Ib	2012	8	
2566_K56684_SRR3320528	CC10	carrier	Kenya	Ib	2012	10	
2567_K56668_SRR3320527	CC10	carrier	Kenya	II	2012	10	
2568_K56667_SRR3320526	CC1	carrier	Kenya	V	2012	1	
2569_K56665_SRR3320525	CC17	carrier	Kenya	III	2012	484	
2570_K56664_SRR3320524	CC17	carrier	Kenya	III	2012	484	
2571_K56646_SRR3320523	N/D	carrier	Kenya	V	2012	3	
2572_K56645_SRR3320522	CC17	carrier	Kenya	III	2012	17	
2573_K56635_SRR3320521	CC19	carrier	Kenya	V	2012	327	
2574_K56634_SRR3320520	CC23	carrier	Kenya	Ia	2012	23	
2575_K56619_SRR3320519	CC23	carrier	Kenya	Ia	2012	23	
2576_K56615_SRR3320518	CC17	carrier	Kenya	III	2012	17	
2577_K56614_SRR3320517	CC19	carrier	Kenya	III	2012	182	
2578_K56613_SRR3320516	CC19	carrier	Kenya	II	2012	28	
2579_K56612_SRR3320515	CC23	carrier	Kenya	Ia	2012	498	
2580_K56611_SRR3320514	N/D	carrier	Kenya	Ia	2012	486	
2581_K56610_SRR3320513	CC23	carrier	Kenya	Ia	2012	23	

2582_K56586_SRR3320512	CC10	carrier	Kenya	Ib	2012	10	
2583_K56585_SRR3320511	CC10	carrier	Kenya	Ib	2012	10	
2584_K56583_SRR3320510	CC23	carrier	Kenya	Ia	2012	24	
2585_K56581_SRR3320509	CC23	carrier	Kenya	V	2012	24	
2586_K56570_SRR3320508	CC23	carrier	Kenya	Ia	2012	23	
2587_K56555_SRR3320507	CC23	carrier	Kenya	Ia	2012	23	
2588_K56545_SRR3320506	CC19	carrier	Kenya	II	2012	28	
2589_K56509_SRR3320505	CC19	carrier	Kenya	II	2012	28	
2590_K56508_SRR3320504	CC17	carrier	Kenya	III	2012	17	
2591_K56507_SRR3320503	CC23	carrier	Kenya	Ia	2012	23	
2592_K56506_SRR3320502	CC19	carrier	Kenya	III	2012	19	
2593_K56494_SRR3320501	CC23	carrier	Kenya	Ia	2012	23	
2594_K56493_SRR3320500	CC10	carrier	Kenya	II	2012	10	
2595_K56492_SRR3320499	CC1	carrier	Kenya	V	2012	1	
2596_K56487_SRR3320498	CC1	carrier	Kenya	V	2012	1	
2597_K56486_SRR3320497	CC23	carrier	Kenya	Ia	2012	23	
2598_K56484_SRR3320496	CC23	carrier	Kenya	Ia	2012	498	
2599_K56483_SRR3320495	CC10	carrier	Kenya	II	2012	10	
2600_K56482_SRR3320494	CC19	carrier	Kenya	III	2012	182	
2601_K56479_SRR3320493	CC10	carrier	Kenya	Ib	2012	8	
2602_K56477_SRR3320492	CC23	carrier	Kenya	Ia	2012	23	
2603_K56463_SRR3320491	CC19	carrier	Kenya	III	2012	182	
2604_K56453_SRR3320490	CC1	carrier	Kenya	IV	2012	196	
2605_K56442_SRR3320489	CC23	carrier	Kenya	Ia	2012	23	
2606_K56436_SRR3320488	CC23	carrier	Kenya	Ia	2012	23	
2607_K56435_SRR3320487	CC23	carrier	Kenya	Ia	2012	23	
2608_K56432_SRR3320486	CC23	carrier	Kenya	Ia	2012	23	
2609_K56431_SRR3320485	CC23	carrier	Kenya	Ia	2012	23	
2610_K56419_SRR3320484	CC23	carrier	Kenya	Ia	2012	23	
2611_K56418_SRR3320483	CC23	carrier	Kenya	Ia	2012	23	
2612_K56377_SRR3320482	CC23	carrier	Kenya	Ia	2012	23	
2613_K56374_SRR3320481	CC1	carrier	Kenya	IV	2012	196	
2614_K56373_SRR3320480	CC19	carrier	Kenya	III	2012	182	
2615_K56372_SRR3320479	CC17	carrier	Kenya	III	2012	792	
2616_K56371_SRR3320478	CC17	carrier	Kenya	III	2012	17	
2617_K56370_SRR3320477	CC1	carrier	Kenya	V	2012	1	
2618_K56369_SRR3320476	CC17	carrier	Kenya	III	2012	17	
2619_K56367_SRR3320475	CC19	carrier	Kenya	V	2012	327	
2620_K56366_SRR3320474	CC1	carrier	Kenya	V	2012	1	
2621_K56341_SRR3320473	CC10	carrier	Kenya	Ib	2012	8	
2622_K56333_SRR3320472	CC19	carrier	Kenya	V	2012	327	
2623_K56278_SRR3320471	CC10	carrier	Kenya	Ib	2012	8	
2624_K56264_SRR3320470	CC19	carrier	Kenya	V	2012	19	
2625_K56263_SRR3320469	CC1	carrier	Kenya	V	2012	1	
2626_K56261_SRR3320468	CC19	carrier	Kenya	III	2012	19	
2627_K56247_SRR3320467	CC23	carrier	Kenya	Ia	2012	23	
2628_K56246_SRR3320466	CC17	carrier	Kenya	III	2012	17	
2629_K56245_SRR3320465	CC17	carrier	Kenya	III	2012	17	
2630_K56244_SRR3320464	CC1	carrier	Kenya	V	2012	1	
2631_K56235_SRR3320463	CC17	carrier	Kenya	III	2012	788	
2632_K56234_SRR3320462	CC23	carrier	Kenya	Ia	2012	23	
2633_K56233_SRR3320461	CC23	carrier	Kenya	Ia	2012	23	
2634_K56232_SRR3320460	CC23	carrier	Kenya	Ia	2012	23	
2635_K56231_SRR3320459	CC17	carrier	Kenya	III	2012	17	
2636_K56228_SRR3320458	CC17	carrier	Kenya	III	2012	17	
2637_K56218_SRR3320457	CC17	carrier	Kenya	III	2012	17	
2638_K56217_SRR3320456	CC23	carrier	Kenya	Ia	2012	23	
2639_K56198_SRR3320455	CC23	carrier	Kenya	Ia	2012	23	
2640_K56197_SRR3320454	CC19	carrier	Kenya	III	2012	19	
2641_K56196_SRR3320453	CC10	carrier	Kenya	Ib	2012	10	
2642_K56195_SRR3320452	CC17	carrier	Kenya	III	2012	801	
2643_K56194_SRR3320451	CC19	carrier	Kenya	III	2012	182	

2644_K56193_SRR3320450	CC19	carrier	Kenya	II	2012	28	
2645_K56186_SRR3320449	CC23	carrier	Kenya	Ia	2012	498	
2646_K56185_SRR3320448	CC17	carrier	Kenya	III	2012	17	
2647_K56184_SRR3320447	CC19	carrier	Kenya	III	2012	182	
2648_K56183_SRR3320446	CC17	carrier	Kenya	III	2012	17	
2649_K56159_SRR3320445	CC1	carrier	Kenya	V	2012	1	
2650_K56114_SRR3320444	CC1	carrier	Kenya	V	2012	1	
2651_K56113_SRR3320443	CC23	carrier	Kenya	Ia	2012	23	
2652_K56076_SRR3320442	CC10	carrier	Kenya	Ib	2012	10	
2653_K56074_SRR3320441	CC19	carrier	Kenya	III	2012	182	
2654_K56073_SRR3320440	CC19	carrier	Kenya	III	2012	182	
2655_K56071_SRR3320439	CC17	carrier	Kenya	III	2012	484	
2656_K56070_SRR3320438	CC10	carrier	Kenya	Ib	2012	10	
2657_K56069_SRR3320437	CC23	carrier	Kenya	Ia	2012	23	
2658_K56064_SRR3320436	CC17	carrier	Kenya	III	2012	484	
2659_K56062_SRR3320435	CC17	carrier	Kenya	III	2012	17	
2660_K56061_SRR3320434	CC17	carrier	Kenya	III	2012	484	
2661_K56046_SRR3320433	CC19	carrier	Kenya	III	2012	182	
2662_K56031_SRR3320432	CC17	carrier	Kenya	III	2012	484	
2663_K55997_SRR3320431	CC23	carrier	Kenya	Ia	2012	23	
2664_K55996_SRR3320430	CC10	carrier	Kenya	Ib	2012	8	
2665_K55980_SRR3320429	CC23	carrier	Kenya	Ia	2012	23	
2666_K55979_SRR3320428	CC23	carrier	Kenya	V	2012	23	
2667_K55960_SRR3320427	CC19	carrier	Kenya	V	2012	327	
2668_K55959_SRR3320426	CC19	carrier	Kenya	III	2012	182	
2669_K55958_SRR3320425	CC19	carrier	Kenya	II	2012	28	
2670_K55896_SRR3320424	CC23	carrier	Kenya	Ia	2012	23	
2671_K55895_SRR3320423	CC19	carrier	Kenya	III	2012	182	
2672_K55881_SRR3320422	CC17	carrier	Kenya	III	2012	484	
2673_K55880_SRR3320421	CC23	carrier	Kenya	Ia	2012	23	
2674_K55761_SRR3320420	CC23	carrier	Kenya	Ia	2012	23	
2675_K55711_SRR3320419	CC1	carrier	Kenya	V	2012	796	
2676_K55655_SRR3320418	CC17	carrier	Kenya	III	2012	801	
2677_K55631_SRR3320417	CC17	carrier	Kenya	III	2012	17	
2678_K55630_SRR3320416	CC10	carrier	Kenya	Ib	2012	10	
2679_K55603_SRR3320415	CC17	carrier	Kenya	III	2012	17	
2680_K55594_SRR3320414	CC17	carrier	Kenya	III	2012	484	
2681_K55593_SRR3320413	CC17	carrier	Kenya	III	2012	17	
2682_K55547_SRR3320412	CC17	carrier	Kenya	III	2012	17	
2683_K55532_SRR3320411	CC23	carrier	Kenya	Ia	2012	23	
2684_K55531_SRR3320410	CC10	carrier	Kenya	II	2012	10	
2685_K55530_SRR3320409	CC19	carrier	Kenya	V	2012	328	
2686_K55529_SRR3320408	CC10	carrier	Kenya	II	2012	10	
2687_K55513_SRR3320407	CC19	carrier	Kenya	V	2012	328	
2688_K55494_SRR3320406	N/D	carrier	Kenya	Ib	2012	785	
2689_K55493_SRR3320405	CC17	carrier	Kenya	III	2012	17	
2690_K55454_SRR3320404	CC10	carrier	Kenya	Ib	2012	8	
2691_K55453_SRR3320403	CC1	carrier	Kenya	V	2012	1	
2692_K55452_SRR3320402	CC23	carrier	Kenya	V	2012	24	
2693_K55442_SRR3320401	CC23	carrier	Kenya	Ia	2012	23	
2694_K55441_SRR3320400	CC23	carrier	Kenya	Ia	2012	23	
2695_K55427_SRR3320399	CC19	carrier	Kenya	V	2012	328	
2696_K55425_SRR3320398	CC10	carrier	Kenya	Ib	2012	10	
2697_K55416_SRR3320397	CC17	carrier	Kenya	III	2012	17	
2698_K55398_SRR3320396	CC19	carrier	Kenya	V	2012	327	
2699_K55357_SRR3320395	CC23	carrier	Kenya	V	2012	23	
2700_K55356_SRR3320394	CC19	carrier	Kenya	V	2012	327	
2701_K55320_SRR3320393	CC17	carrier	Kenya	III	2012	17	
2702_K55292_SRR3320392	CC17	carrier	Kenya	III	2012	17	
2703_K55286_SRR3320391	CC17	carrier	Kenya	IV	2012	291	
2704_K55280_SRR3320390	CC23	carrier	Kenya	Ia	2012	23	
2705_K55234_SRR3320389	CC10	carrier	Kenya	Ib	2012	10	

2706_K55219_SRR3320388	CC1	carrier	Kenya	IV	2012	196	
2707_K55218_SRR3320387	CC10	carrier	Kenya	Ib	2012	8	
2708_K52864_SRR3320386	CC23	carrier	Kenya	Ia	2012	23	
2709_K52862_SRR3320385	CC17	carrier	Kenya	III	2012	17	
2710_K52817_SRR3320384	CC19	carrier	Kenya	V	2012	327	
2711_K52808_SRR3320383	CC19	carrier	Kenya	II	2012	28	
2712_K52792_SRR3320382	CC10	carrier	Kenya	Ib	2012	10	
2713_K52781_SRR3320381	CC1	carrier	Kenya	V	2012	1	
2714_K52780_SRR3320380	CC19	carrier	Kenya	III	2012	182	
2715_K52779_SRR3320379	CC19	carrier	Kenya	V	2012	327	
2716_K52774_SRR3320378	CC23	carrier	Kenya	Ia	2012	23	
2717_K52773_SRR3320377	CC17	carrier	Kenya	III	2012	17	
2718_K52692_SRR3320376	CC23	carrier	Kenya	Ia	2012	23	
2719_K52674_SRR3320375	CC10	carrier	Kenya	Ib	2012	10	
2720_K52662_SRR3320374	CC10	carrier	Kenya	II	2012	10	
2721_K52647_SRR3320373	CC17	carrier	Kenya	III	2012	17	
2722_K52646_SRR3320372	CC17	carrier	Kenya	III	2012	17	
2723_K52623_SRR3320371	CC17	carrier	Kenya	III	2012	17	
2724_K52615_SRR3320370	CC19	carrier	Kenya	III	2012	182	
2725_K52539_SRR3320369	CC23	carrier	Kenya	Ia	2012	23	
2726_K52487_SRR3320368	CC1	carrier	Kenya	V	2012	1	
2727_K52486_SRR3320367	CC17	carrier	Kenya	III	2012	17	
2728_K52459_SRR3320366	CC10	carrier	Kenya	Ib	2012	10	
2729_K52458_SRR3320365	CC1	carrier	Kenya	IV	2012	196	
2730_K52454_SRR3320364	CC19	carrier	Kenya	V	2012	327	
2731_K52452_SRR3320363	CC23	carrier	Kenya	V	2012	24	
2732_K52433_SRR3320362	CC1	carrier	Kenya	V	2012	1	
2733_K52432_SRR3320361	CC10	carrier	Kenya	II	2012	10	
2734_K52396_SRR3320360	CC19	carrier	Kenya	III	2012	182	
2735_K52368_SRR3320359	CC1	carrier	Kenya	V	2012	1	
2736_K52367_SRR3320358	CC17	carrier	Kenya	III	2012	17	
2737_K52346_SRR3320357	CC17	carrier	Kenya	III	2012	17	
2738_K52345_SRR3320356	CC19	carrier	Kenya	III	2012	19	
2739_K52318_SRR3320355	CC1	carrier	Kenya	V	2012	1	
2740_K52315_SRR3320354	CC17	carrier	Kenya	III	2012	17	
2741_K52314_SRR3320353	CC10	carrier	Kenya	Ib	2012	8	
2742_K52274_SRR3320352	CC1	carrier	Kenya	V	2012	1	
2743_K52157_SRR3320351	CC19	carrier	Kenya	V	2012	328	
2744_K52086_SRR3320350	CC10	carrier	Kenya	Ib	2012	787	
2745_K52085_SRR3320349	CC19	carrier	Kenya	III	2012	182	
2746_K52084_SRR3320348	CC23	carrier	Kenya	Ia	2012	23	
2747_K52083_SRR3320347	CC1	carrier	Kenya	V	2012	1	
2748_K52082_SRR3320346	CC19	carrier	Kenya	II	2012	28	
2749_K52067_SRR3320345	CC19	carrier	Kenya	II	2012	28	
2750_K52065_SRR3320344	CC17	carrier	Kenya	III	2012	801	
2751_K52064_SRR3320343	CC10	carrier	Kenya	Ib	2012	10	
2752_K52050_SRR3320342	CC1	carrier	Kenya	V	2012	1	
2753_K52049_SRR3320341	CC17	carrier	Kenya	III	2012	17	
2754_K52048_SRR3320340	CC19	carrier	Kenya	II	2012	28	
2755_K51965_SRR3320339	CC19	carrier	Kenya	V	2012	327	
2756_K51887_SRR3320338	CC19	carrier	Kenya	V	2011	327	
2757_K51855_SRR3320337	CC19	carrier	Kenya	II	2011	28	
2758_K51818_SRR3320336	CC17	carrier	Kenya	III	2011	17	
2759_K51792_SRR3320335	CC10	carrier	Kenya	Ib	2011	10	
2760_K51745_SRR3320334	CC17	carrier	Kenya	III	2011	17	
2761_K51742_SRR3320333	CC17	carrier	Kenya	III	2011	17	
2762_K51738_SRR3320332	CC17	carrier	Kenya	III	2011	17	
2763_K51705_SRR3320331	CC19	carrier	Kenya	III	2011	19	
2764_K51704_SRR3320330	N/D	carrier	Kenya	Ib	2011		
2765_K51698_SRR3320329	CC1	carrier	Kenya	V	2011	1	
2766_K51617_SRR3320328	CC17	carrier	Kenya	III	2011	17	
2767_K51608_SRR3320327	CC17	carrier	Kenya	III	2011	17	

2768_K51590_SRR3320326	CC17	carrier	Kenya	III	2011	484	
2769_K51578_SRR3320325	CC17	carrier	Kenya	III	2011	17	
2770_K51572_SRR3320324	CC17	carrier	Kenya	III	2011	484	
2771_K51527_SRR3320323	CC19	carrier	Kenya	III	2011	182	
2772_K51526_SRR3320322	CC1	carrier	Kenya	II	2011	1	
2773_K51525_SRR3320321	CC19	carrier	Kenya	II	2011	28	
2774_K51478_SRR3320320	CC23	carrier	Kenya	Ia	2011	23	
2775_K51463_SRR3320319	CC10	carrier	Kenya	Ib	2011	8	
2776_K51420_SRR3320318	CC1	carrier	Kenya	V	2011	796	
2777_K51361_SRR3320317	CC10	carrier	Kenya	Ib	2011	10	
2778_K51349_SRR3320316	CC1	carrier	Kenya	IV	2011	196	
2779_K51348_SRR3320315	CC17	carrier	Kenya	III	2011	484	
2780_K51329_SRR3320314	CC23	carrier	Kenya	Ia	2011	23	
2781_K51319_SRR3320313	CC1	carrier	Kenya	IV	2011	196	
2782_K51318_SRR3320312	CC1	carrier	Kenya	II	2011	2	
2783_K51280_SRR3320311	CC19	carrier	Kenya	II	2011	28	
2784_K51261_SRR3320310	CC10	carrier	Kenya	Ib	2011	8	
2785_K51260_SRR3320309	CC17	carrier	Kenya	III	2011	17	
2786_K51258_SRR3320308	CC1	carrier	Kenya	V	2011	1	
2787_K51257_SRR3320307	CC23	carrier	Kenya	Ia	2011	23	
2788_K51256_SRR3320306	CC19	carrier	Kenya	III	2011	182	
2789_K51255_SRR3320305	CC23	carrier	Kenya	Ia	2011	802	
2790_K51230_SRR3320304	CC10	carrier	Kenya	Ib	2011	10	
2791_K51216_SRR3320303	CC19	carrier	Kenya	II	2011	28	
2792_K51201_SRR3320302	CC17	carrier	Kenya	III	2011	17	
2793_K51175_SRR3320301	CC23	carrier	Kenya	Ia	2011	23	
2794_K51157_SRR3320300	CC19	carrier	Kenya	III	2011	182	
2795_K51139_SRR3320299	CC10	carrier	Kenya	NT	2011	8	
2796_K51133_SRR3320298	CC19	carrier	Kenya	V	2011	327	
2797_K51118_SRR3320297	CC19	carrier	Kenya	III	2011	182	
2798_K51091_SRR3320296	CC17	carrier	Kenya	III	2011	17	
2799_K50972_SRR3320295	CC17	carrier	Kenya	III	2011	17	
2800_K50939_SRR3320294	CC19	carrier	Kenya	III	2011	182	
2801_K50938_SRR3320293	CC23	carrier	Kenya	Ia	2011	23	
2802_K50909_SRR3320292	CC10	carrier	Kenya	Ib	2011	10	
2803_K50877_SRR3320291	CC17	carrier	Kenya	III	2011	784	
2804_K50864_SRR3320290	CC10	carrier	Kenya	Ib	2011	10	
2805_K50827_SRR3320289	CC19	carrier	Kenya	II	2011	28	
2806_K50826_SRR3320288	CC19	carrier	Kenya	II	2011	28	
2807_K50792_SRR3320287	CC17	carrier	Kenya	III	2011	17	
2808_K50791_SRR3320286	CC17	carrier	Kenya	III	2011	17	
2809_K50790_SRR3320285	CC23	carrier	Kenya	Ia	2011	23	
2810_K50725_SRR3320284	CC23	carrier	Kenya	Ia	2011	23	
2811_K50724_SRR3320283	CC17	carrier	Kenya	III	2011	17	
2812_K50709_SRR3320282	CC17	carrier	Kenya	III	2011	484	
2813_K50708_SRR3320281	CC23	carrier	Kenya	Ia	2011	23	
2814_K50669_SRR3320280	CC1	carrier	Kenya	IV	2011	196	
2815_K50525_SRR3320279	CC10	carrier	Kenya	Ib	2011	8	
2816_K50492_SRR3320278	CC23	carrier	Kenya	Ia	2011	23	
2817_K50466_SRR3320277	CC17	carrier	Kenya	III	2011	788	
2818_K50442_SRR3320276	CC10	carrier	Kenya	Ib	2011	8	
2819_K50441_SRR3320275	CC17	carrier	Kenya	III	2011	17	
2820_K50426_SRR3320274	CC19	carrier	Kenya	III	2011	182	
2821_K50415_SRR3320273	CC1	carrier	Kenya	V	2011	1	
2822_K50414_SRR3320272	CC17	carrier	Kenya	III	2011	17	
2823_K50387_SRR3320271	CC10	carrier	Kenya	Ib	2011	10	
2824_K50365_SRR3320270	CC10	carrier	Kenya	Ib	2011	8	
2825_K50364_SRR3320269	CC19	carrier	Kenya	V	2011	327	
2826_K50342_SRR3320268	CC23	carrier	Kenya	Ia	2011	23	
2827_K50227_SRR3320267	CC1	carrier	Kenya	V	2011	796	
2828_K50223_SRR3320266	CC17	carrier	Kenya	III	2011	17	
2829_K50222_SRR3320265	CC19	carrier	Kenya	III	2011	182	

2830_K50167_SRR3320264	CC19	carrier	Kenya	III	2011	182	
2831_K50146_SRR3320263	CC17	carrier	Kenya	III	2011	484	
2832_K50145_SRR3320262	CC23	carrier	Kenya	Ia	2011	23	
2833_K50144_SRR3320261	CC10	carrier	Kenya	Ib	2011	8	
2834_K50143_SRR3320260	CC19	carrier	Kenya	III	2011	182	
2835_K50142_SRR3320259	CC17	carrier	Kenya	III	2011	484	
2836_K50140_SRR3320258	CC17	carrier	Kenya	III	2011	17	
2837_K50057_SRR3320257	CC17	carrier	Kenya	III	2011	484	
2838_K50009_SRR3320256	CC19	carrier	Kenya	V	2011	327	
2839_K50008_SRR3320255	CC19	carrier	Kenya	II	2011	28	
2840_K50007_SRR3320254	CC19	carrier	Kenya	III	2011	19	
2841_K50006_SRR3320253	CC19	carrier	Kenya	II	2011	28	
2842_K49986_SRR3320252	CC19	carrier	Kenya	III	2011	19	
2843_K49981_SRR3320251	CC17	carrier	Kenya	III	2011	17	
2844_K49970_SRR3320250	CC17	carrier	Kenya	III	2011	17	
2845_K49969_SRR3320249	CC1	carrier	Kenya	V	2011	1	
2846_K49965_SRR3320248	CC17	carrier	Kenya	III	2011	17	
2847_K49933_SRR3320247	CC17	carrier	Kenya	III	2011	484	
2848_K49931_SRR3320246	CC23	carrier	Kenya	Ia	2011	23	
2849_K49917_SRR3320245	CC23	carrier	Kenya	Ia	2011	23	
2850_K49870_SRR3320244	CC10	carrier	Kenya	Ib	2011	10	
2851_K49839_SRR3320243	CC19	carrier	Kenya	III	2011	19	
2852_K49831_SRR3320242	CC17	carrier	Kenya	III	2011	484	
2853_K49793_SRR3320241	CC23	carrier	Kenya	Ia	2011	23	
2854_K49791_SRR3320240	CC23	carrier	Kenya	Ia	2011	23	
2855_K49768_SRR3320239	CC19	carrier	Kenya	II	2011	28	
2856_K49740_SRR3320238	CC17	carrier	Kenya	III	2011	17	
2857_K49739_SRR3320237	CC10	carrier	Kenya	Ib	2011	8	
2858_K49738_SRR3320236	CC17	carrier	Kenya	III	2011	484	
2859_K23965_SRR3320235	CC17	invasive	Kenya	III	2007	17	
2860_K23286_SRR3320234	CC23	invasive	Kenya	Ia	2007	23	
2861_K20349_SRR3320233	CC17	invasive	Kenya	III	2006	17	
2862_K20332_SRR3320232	CC17	invasive	Kenya	III	2006	17	
2863_K20251_SRR3320231	CC10	invasive	Kenya	Ib		804	
2864_K20230_SRR3320230	CC10	invasive	Kenya	Ib	2006	8	
2865_K20019_SRR3320229	CC17	invasive	Kenya	III	2006	17	
2866_K19924_SRR3320228	CC17	invasive	Kenya	III	2006	484	
2867_K19244_SRR3320227	CC17	invasive	Kenya	III	2006	17	
2868_K19212_SRR3320226	CC17	invasive	Kenya	III	2006	17	
2869_K19170_SRR3320225	N/D	invasive	Kenya	Ia	2006	803	
2870_K18723_SRR3320224	CC17	invasive	Kenya	III	2006	484	
2871_K17900_SRR3320223	CC17	invasive	Kenya	III	2006	17	
2872_K17897_SRR3320222	CC17	invasive	Kenya	III	2006	147	
2873_K17389_SRR3320221	CC17	invasive	Kenya	III	2005	17	
2874_K17308_SRR3320220	CC17	invasive	Kenya	III	2005	17	
2875_K17224_SRR3320219	CC1	invasive	Kenya	IV	2005	196	
2876_K17110_SRR3320218	CC17	invasive	Kenya	III	2005	17	
2877_K16827_SRR3320217	CC10	invasive	Kenya	Ib	2005	10	
2878_K16755_SRR3320216	CC17	invasive	Kenya	III	2005	17	
2879_K16534_SRR3320215	CC17	invasive	Kenya	III	2005	17	
2880_K14206_SRR3320214	CC17	invasive	Kenya	III	2005	17	
2881_K13758_SRR3320213	CC17	invasive	Kenya	III	2004	17	
2882_K11385_SRR3320212	CC17	invasive	Kenya	III	2003	17	
2883_K59563_SRR3320211	CC17	invasive	Kenya	III	2012	17	
2884_K57810_SRR3320210	CC17	invasive	Kenya	III	2012	17	
2885_K57085_SRR3320209	CC17	invasive	Kenya	III	2012	17	
2886_K57018_SRR3320208	CC1	invasive	Kenya	II	2012	2	
2887_K10268_SRR3320207	CC17	invasive	Kenya	III	2003	17	
2888_K56640_SRR3320206	CC17	invasive	Kenya	III	2012	17	
2889_K55826_SRR3320205	CC17	invasive	Kenya	III	2012	17	
2890_K55813_SRR3320204	CC17	invasive	Kenya	III	2012	17	
2891_K9182_SRR3320203	CC17	invasive	Kenya	III	2002	17	

2892_K52758_SRR3320202	CC23	invasive	Kenya	Ia	2012	23	
2893_K8568_SRR3320201	CC23	invasive	Kenya	Ia	2002	23	
2894_K8555_SRR3320200	CC17	invasive	Kenya	III	2002	17	
2895_K8309_SRR3320199	CC17	invasive	Kenya	III	2002	17	
2896_K50468_SRR3320198	CC17	invasive	Kenya	III		792	
2897_K7834_SRR3320197	CC10	invasive	Kenya	Ib	2001	10	
2898_K48612_SRR3320196	CC17	invasive	Kenya	III	2011	484	
2899_K7453_SRR3320195	CC19	invasive	Kenya	III	2001	19	
2900_K48051_SRR3320194	CC17	invasive	Kenya	III	2011	17	
2901_K7229_SRR3320193	CC17	invasive	Kenya	III	2001	17	
2902_K47340_SRR3320192	CC17	invasive	Kenya	III	2011	484	
2903_K7129_SRR3320191	CC23	invasive	Kenya	Ia	2001	23	
2904_K6781_SRR3320190	CC23	invasive	Kenya	Ia	2001	23	
2905_K45678_SRR3320189	CC17	invasive	Kenya	III	2010	17	
2906_K6666_SRR3320188	CC17	invasive	Kenya	III	2000	484	
2907_K6625_SRR3320187	CC17	invasive	Kenya	III	2000	17	
2908_K6245_SRR3320186	CC17	invasive	Kenya	III	2000	17	
2909_K6234_SRR3320185	CC17	invasive	Kenya	III	2000	17	
2910_K6229_SRR3320184	CC17	invasive	Kenya	III	2000	17	
2911_K6209_SRR3320183	CC23	invasive	Kenya	Ia	2000	23	
2912_K5666_SRR3320182	CC23	invasive	Kenya	Ia	2000	23	
2913_K5136_SRR3320181	CC17	invasive	Kenya	III	1999	17	
2914_K4958_SRR3320180	CC17	invasive	Kenya	III	1999	17	
2915_K39258_SRR3320179	CC17	invasive	Kenya	III	2009	484	
2916_K38783_SRR3320178	CC17	invasive	Kenya	III	2009	17	
2917_K36829_SRR3320177	CC17	invasive	Kenya	III	2009	484	
2918_K4436_SRR3320176	CC23	invasive	Kenya	Ia	1999	23	
2919_K4340_SRR3320175	CC17	invasive	Kenya	III	1999	17	
2920_K36235_SRR3320174	CC19	invasive	Kenya	III	2009	182	
2921_K36205_SRR3320173	CC19	invasive	Kenya	III	2009	182	
2922_K35752_SRR3320172	CC10	invasive	Kenya	Ib	2008	8	
2923_K3931_SRR3320171	CC23	invasive	Kenya	Ia	1998	23	
2924_K33921_SRR3320170	CC17	invasive	Kenya	III	2008	17	
2925_K3605_SRR3320169	CC17	invasive	Kenya	III	1998	17	
2926_K33534_SRR3320168	CC17	invasive	Kenya	III	2008	17	
2927_K31176_SRR3320167	CC17	invasive	Kenya	III	2008	17	
2928_K28931_SRR3320166	CC17	invasive	Kenya	III	2008	17	
2929_K28764_SRR3320165	CC23	invasive	Kenya	Ia	2008	23	
2930_K25271_SRR3320164	CC19	invasive	Kenya	III	2007	182	
3018_LZC0821	CC23	carrier	Malawi	V		223	ERR1910505
3019_LZC3113	CC19	carrier	Malawi	III		182	ERR1910506
3021_B17570	N/D	invasive	Malawi	II			ERR1910508
3022_LZA8818	CC23	carrier	Malawi	Ia		23	ERR1910509
3025_LZC3822	CC23	carrier	Malawi	V		223	ERR1910512
3027_LZC7155	CC17	carrier	Malawi	III		17	ERR1910514
3028_BKQ2CT	CC23	invasive	Malawi	Ia		23	ERR1910515
3029_LZA9312	CC19	carrier	Malawi	III		19	ERR1910516
3030_BKR2A0	N/D	invasive	Malawi	III			ERR1910517
3032_D34169	CC19	invasive	Malawi	V		327	ERR1910519
3033_D31559	CC17	invasive	Malawi	III		17	ERR1910520
3034_LZE6322	N/D	unknown	Malawi	NT			ERR1910521
3035_BKR25R	CC17	invasive	Malawi	III		866	ERR1910522
3036_LZE9626	CC19	carrier	Malawi	III		182	ERR1910523
3037_LZA8762	CC23	carrier	Malawi	Ia		23	ERR1910524
3038_LZC4964	CC17	carrier	Malawi	III		17	ERR1910525
3040_LZE8310	CC23	carrier	Malawi	NT		23	ERR1910527
3041_LZC5852	CC17	carrier	Malawi	III		17	ERR1910528
3043_LZE9097	CC23	carrier	Malawi	V		23	ERR1910530
3044_LZE8393	CC17	carrier	Malawi	III		866	ERR1910531
3046_1029448	CC17	invasive	Malawi	III		17	ERR1910533
3047_LZE0977	CC23	carrier	Malawi	Ia		23	ERR1910534
3048_1037160	CC19	invasive	Malawi	V		327	ERR1910535

3050_LZE7243	CC19	carrier	Malawi	III		182	ERR1910537
3051_BCN134	CC23	invasive	Malawi	Ia		23	ERR1910538
3052_LZC6769	CC23	carrier	Malawi	Ia		23	ERR1910539
3053_D26167	CC17	invasive	Malawi	III		17	ERR1910540
3054_BKQCW3	CC17	invasive	Malawi	III		17	ERR1910541
3056_LZE4647	CC23	carrier	Malawi	V		223	ERR1910543
3058_LZE7260	CC17	carrier	Malawi	III		17	ERR1910545
3060_LZC4270	CC23	carrier	Malawi	V		223	ERR1910547
3061_LZA9667	CC23	carrier	Malawi	Ia		23	ERR1910548
3062_LZE6179	CC23	unknown	Malawi	V		223	ERR1910549
3063_D36128	CC17	invasive	Malawi	III		17	ERR1910550
3064_D34047	N/D	invasive	Malawi	V			ERR1910551
3065_LZE0969	CC23	carrier	Malawi	Ia		23	ERR1910552
3066_LZC5480	CC17	carrier	Malawi	III		17	ERR1910553
3068_LZE1214	CC17	carrier	Malawi	III		17	ERR1910555
3069_LZA4841	CC23	carrier	Malawi	Ia		23	ERR1910556
3070_LZC2799	CC19	carrier	Malawi	V		327	ERR1910557
3071_LZE8125	CC23	carrier	Malawi	Ia		23	ERR1910558
3072_LZE6689	CC23	carrier	Malawi	NT		23	ERR1910559
3073_LZA4972	CC23	carrier	Malawi	Ia		23	ERR1910560
3074_LZE6242	CC23	carrier	Malawi	NT		23	ERR1910561
3075_LZE9208	CC19	carrier	Malawi	V		327	ERR1910562
3076_C12087	CC17	invasive	Malawi	III		17	ERR1910563
3077_D32852	CC10	invasive	Malawi	II		10	ERR1910564
3078_LZE8711	CC23	carrier	Malawi	Ia		802	ERR1910565
3079_BKR239	CC17	invasive	Malawi	III		866	ERR1910566
3080_D33484	CC19	invasive	Malawi	V		327	ERR1910567
3081_LZC8192	CC23	carrier	Malawi	Ia		23	ERR1910568
3082_LZE8674	CC19	carrier	Malawi	III		182	ERR1910569
3083_LZE7286	CC23	carrier	Malawi	V		24	ERR1910570
3085_LZE6881	CC17	carrier	Malawi	III		17	ERR1910572
3087_LZA7325	CC19	carrier	Malawi	III		19	ERR1910574
3088_D53208	CC17	invasive	Malawi	III		17	ERR1910575
3089_LZC9860	CC23	carrier	Malawi	V		223	ERR1910576
3090_BKR5LM	CC17	invasive	Malawi	III		17	ERR1910577
3093_ZAE877	CC19	invasive	Malawi	V		327	ERR1910580
3095_LZE9257	CC19	carrier	Malawi	III		182	ERR1910582
3096_LQX0032	CC23	carrier	Malawi	Ia		23	ERR1910583
3098_LZE8424	CC19	carrier	Malawi	V		327	ERR1910585
3099_LZC4655	CC19	carrier	Malawi	V		19	ERR1910586
3100_LZE6630	CC19	unknown	Malawi	V		327	ERR1910587
3102_LZE8553	CC23	carrier	Malawi	Ia		23	ERR1910589
3103_D32743	CC17	invasive	Malawi	III		17	ERR1910590
3105_LZE9136	CC23	carrier	Malawi	Ia		23	ERR1910592
3107_C15370	CC17	invasive	Malawi	III		17	ERR1910594
3109_LZE4702	CC23	carrier	Malawi	Ia		23	ERR1910596
3110_LZE6988	CC19	carrier	Malawi	III		19	ERR1910597
3111_LZC4930	CC17	carrier	Malawi	III		17	ERR1910598
3112_LZE6146	CC10	carrier	Malawi	Ib		10	ERR1910599
3113_LZA6701	CC17	carrier	Malawi	III		17	ERR1910600
3114_LZE4270	CC23	carrier	Malawi	V		223	ERR1910601
3115_LZE9273	CC19	carrier	Malawi	III		182	ERR1910602
3116_LZE8264	CC10	carrier	Malawi	NT		8	ERR1910603
3117_LZE9595	CC19	carrier	Malawi	NT		327	ERR1910604
3118_LZE9683	CC23	carrier	Malawi	V		223	ERR1910605
3119_LZE8711	CC23	carrier	Malawi	Ia		802	ERR1910606
3120_LZC7585	CC17	carrier	Malawi	III		17	ERR1910607
3122_LZC7616	CC17	carrier	Malawi	III		17	ERR1910609
3125_LZE9520	CC23	carrier	Malawi	Ia		23	ERR1910612
3126_LZC7083	CC17	carrier	Malawi	III		17	ERR1910613
3127_D28060	CC17	invasive	Malawi	III		17	ERR1910614
3129_D32479	CC17	invasive	Malawi	III		17	ERR1910616

3131_D30738	CC17	invasive	Malawi	III		109	ERR1910618
3132_LABZAE908	CC10	carrier	Malawi	Ib		8	ERR1910619
3133_D40064	CC17	invasive	Malawi	III		17	ERR1910620
3134_LZC1351	CC23	carrier	Malawi	Ia		23	ERR1910621
3135_LZC5367	CC23	carrier	Malawi	V		223	ERR1910622
3136_C11861	CC17	invasive	Malawi	III		17	ERR1910623
3138_LZC3806	CC19	carrier	Malawi	V		327	ERR1910625
3139_LZE4288_A	CC23	unknown	Malawi	V		223	ERR1910626
3140_1038262	CC17	invasive	Malawi	III		17	ERR1910627
3141_LZA5406	CC17	carrier	Malawi	III		17	ERR1910628
3142_D26122	CC10	invasive	Malawi	Ib		8	ERR1910629
3143_D31951	CC17	invasive	Malawi	III		17	ERR1910630
3144_LZC3558	CC19	carrier	Malawi	III		182	ERR1910631
3145_4Cath	N/D	invasive	Malawi	II			ERR1910632
3146_LZC5375	CC10	carrier	Malawi	Ib		10	ERR1910633
3147_LZC1982	CC19	carrier	Malawi	NT		327	ERR1910634
3148_LZE6912	CC17	carrier	Malawi	III		17	ERR1910635
3149_D29545	CC10	invasive	Malawi	Ib		8	ERR1910636
3151_D43024	CC17	invasive	Malawi	III		17	ERR1910638
3152_D31483	CC17	invasive	Malawi	III		17	ERR1910639
3153_LZE0686	CC23	carrier	Malawi	V		223	ERR1910640
3154_C14639	CC17	invasive	Malawi	III		109	ERR1910641
3155_LZA8795	CC10	carrier	Malawi	NT		8	ERR1910642
3156_BKQG72	CC17	invasive	Malawi	III		17	ERR1910643
3158_C16147	CC17	invasive	Malawi	III		17	ERR1910645
3159_C19342	CC17	invasive	Malawi	III		17	ERR1910646
3160_LZC5498	CC17	carrier	Malawi	III		17	ERR1910647
3161_D33035	CC17	invasive	Malawi	III		17	ERR1910648
3162_LZC1773	CC19	carrier	Malawi	V		327	ERR1910649
3163_LZE1046	CC17	carrier	Malawi	III		17	ERR1910650
3164_LZE4288_B	CC23	unknown	Malawi	V		223	ERR1910651
3165_LZA7753	CC23	carrier	Malawi	Ia		23	ERR1910652
3166_LZE6769	CC19	carrier	Malawi	V		327	ERR1910653
3167_LZE8002	CC23	carrier	Malawi	Ia		23	ERR1910654
3168_BKQFT6	CC17	invasive	Malawi	III		17	ERR1910655
3169_LABZAC037	CC23	carrier	Malawi	NT		23	ERR1910656
3170_LZE9546	CC10	carrier	Malawi	II		10	ERR1910657
3172_C17639	CC17	invasive	Malawi	III		17	ERR1910659
3173_BKQFUX	CC23	invasive	Malawi	Ia		23	ERR1910660
3174_BKQ51E	CC23	invasive	Malawi	Ia		23	ERR1910661
3175_D31110	CC23	invasive	Malawi	Ia		23	ERR1910662
3176_LZC5318	CC17	carrier	Malawi	III		866	ERR1910663
3179_D28851	CC17	invasive	Malawi	III		17	ERR1910666
3180_CI7628	CC17	invasive	Malawi	III		17	ERR1910667
3181_LZA5220	CC23	carrier	Malawi	Ia		23	ERR1910668
3182_LZE9030	CC19	unknown	Malawi	V		327	ERR1910669
3184_LZC5713	CC10	carrier	Malawi	Ib		10	ERR1910671
3186_C16300	CC23	invasive	Malawi	Ia		23	ERR1910673
3187_A49324	CC19	invasive	Malawi	V		327	ERR1910674
3188_LZA6710	CC10	carrier	Malawi	Ib		8	ERR1910675
3189_LZG1247	CC17	carrier	Malawi	III		17	ERR1910676
3190_D33180	CC17	invasive	Malawi	III		17	ERR1910677
3191_D35616	CC17	invasive	Malawi	III		866	ERR1910678
3192_LZA8512	CC17	carrier	Malawi	III		17	ERR1910679
3193_BKQE3J	CC17	invasive	Malawi	III		17	ERR1910680
3194_LZE1003	CC17	carrier	Malawi	III		17	ERR1910681
3195_BKR3ZB	CC17	invasive	Malawi	III		17	ERR1910682
3196_D38101	CC17	invasive	Malawi	III		17	ERR1910683
3197_LZA7294	CC23	carrier	Malawi	NT		23	ERR1910684
3198_BHA5YQ	N/D	invasive	Malawi	III			ERR1910685
3199_BKR3EZ	N/D	invasive	Malawi	III			ERR1910686
3200_LZC1610	CC23	carrier	Malawi	Ia		23	ERR1910687

3201_BKQ8W8	CC17	invasive	Malawi	III		17	ERR1910688
3202_LZC1556	CC23	carrier	Malawi	V		223	ERR1910689
3203_A59952	CC19	invasive	Malawi	V		327	ERR1910690
3204_BKQ74D	CC23	invasive	Malawi	Ia		23	ERR1910691
3205_D33407	CC23	invasive	Malawi	Ia		23	ERR1910692
974_NETGBS_STREPCAR_7	CC19	carrier	Malawi	V	2008	327	ERR840658
975_NETGBS_STREPCAR_1586	CC23	carrier	Malawi	Ia	2010	23	ERR840659
976_NETGBS_STREPCAR_15	CC23	carrier	Malawi	Ia	2008	23	ERR840660
977_NETGBS_STREPCAR_1607	CC23	carrier	Malawi	V	2010	223	ERR840661
978_NETGBS_STREPCAR_22	CC23	carrier	Malawi	V	2008	24	ERR840662
979_NETGBS_STREPCAR_1615	N/D	carrier	Malawi	V	2010		ERR840663
981_NETGBS_STREPCAR_1622	CC23	carrier	Malawi	Ia	2010	23	ERR840665
982_NETGBS_STREPCAR_35	CC17	carrier	Malawi	III	2008	17	ERR840666
983_NETGBS_STREPCAR_1640	CC23	carrier	Malawi	V	2010	223	ERR840667
984_NETGBS_STREPCAR_39	CC23	carrier	Malawi	V	2008	223	ERR840668
985_NETGBS_STREPCAR_1645	CC17	carrier	Malawi	III	2010	17	ERR840669
986_NETGBS_STREPCAR_40	CC23	carrier	Malawi	Ia	2008	23	ERR840670
987_NETGBS_STREPCAR_1699	CC17	carrier	Malawi	III	2010	17	ERR840671
989_NETGBS_STREPCAR_1720	CC17	carrier	Malawi	III	2010	17	ERR840673
990_NETGBS_STREPCAR_72	CC19	carrier	Malawi	III	2008	19	ERR840674
991_NETGBS_STREPCAR_1721	CC17	carrier	Malawi	III	2010	17	ERR840675
992_NETGBS_STREPCAR_83	CC19	carrier	Malawi	III	2008	19	ERR840676
993_NETGBS_D25677	CC17	invasive	Malawi	III	2004	109	ERR840677
994_NETGBS_STREPCAR_89	CC17	carrier	Malawi	III	2008	17	ERR840678
995_NETGBS_D25889	CC23	invasive	Malawi	Ia	2004	23	ERR840679
996_NETGBS_STREPCAR_112	CC17	carrier	Malawi	III	2008	866	ERR840680
998_NETGBS_STREPCAR_115	CC17	carrier	Malawi	III	2008	17	ERR840682
999_NETGBS_D26173	CC23	invasive	Malawi	Ia	2004	23	ERR840683
1173_GD201008_001	CC6	animal	Unknown	Ia		7	
1174_FSL_S3_026	CC17	animal	Unknown	NT		67	
1205_CCUG_34230	CC23	animal	Germany	III	1995	23	
1230_CF01173	CC6	animal	Unknown	NT		7	
1231_FSL_C1_487	CC17	animal	Unknown	NT		415	
1232_FSL_C1_494	N/D	animal	Unknown	II			
1233_FSL_F2_338	N/D	unknown	Unknown	II			
1234_FSL_F2_343	CC23	unknown	Unknown	Ia		88	
1235_FSL_S3_001	CC1	unknown	Unknown	V		1	
1236_FSL_S3_003	CC19	unknown	Unknown	III		19	
1237_FSL_S3_005	CC17	unknown	Unknown	II		22	
1238_FSL_S3_014	CC10	unknown	Unknown	Ib		8	
1239_FSL_S3_023	CC1	unknown	Unknown	V		1	
1240_FSL_S3_027	N/D	animal	Unknown	II			
1241_FSL_S3_034	CC17	animal	Unknown	II		61	
1242_FSL_S3_043	CC17	animal	Unknown	NT		61	
1243_FSL_S3_062	N/D	animal	Unknown	II			
1244_FSL_S3_077	N/D	animal	Unknown	II			
1245_FSL_S3_090	CC23	unknown	Unknown	Ia		23	
1246_FSL_S3_102	CC17	unknown	Unknown	III		31	
1247_FSL_S3_128	N/D	animal	Unknown	II			
1248_FSL_S3_137	CC10	unknown	Unknown	Ib		8	
1249_FSL_S3_170	N/D	animal	Unknown	II			
1250_FSL_S3_222	N/D	animal	Unknown	II			
1251_FSL_S3_229	CC17	animal	Unknown	II		415	
1252_FSL_S3_251	N/D	animal	Unknown	III			
1253_FSL_S3_268	CC17	unknown	Unknown	II		22	
1254_FSL_S3_337	CC19	unknown	Unknown	III		19	
1255_FSL_S3_442	CC10	unknown	Unknown	NT		12	
1256_FSL_S3_501	N/D	animal	Unknown	II			
1257_FSL_S3_568	CC17	animal	Unknown	NT		415	
1258_FSL_S3_586	CC17	animal	Unknown	II		67	
1259_FSL_S3_603	CC17	animal	Unknown	III		61	
1260_FSL_S3_608	CC17	animal	Unknown	II		490	

1261_FSL_S3_654	CC17	animal	Unknown	II		61	
1340_LADL_05_108a	N/D	animal	Unknown	Ib		260	
1341_LADL_90_503	N/D	animal	Unknown	NT		260	
1342_LDS_610	N/D	animal	Unknown	NT			
1343_LDS_617	N/D	animal	Unknown	NT			
1344_LDS_623	CC17	animal	Unknown	III		61	
1345_LDS_628	CC17	animal	Unknown	III		61	
1346_LMG_14608	CC6	animal	Unknown	Ia		7	
1347_LMG_14609	N/D	animal	Unknown	NT			
1349_LMG_14838	N/D	animal	Unknown	III			
1350_LMG_15081	CC23	unknown	Unknown	Ia		25	
1351_LMG_15083	CC6	unknown	Unknown	Ia		7	
1352_LMG_15084	CC19	unknown	Unknown	II		19	
1353_LMG_15085	CC17	unknown	Unknown	III		17	
1354_LMG_15089	CC19	unknown	Unknown	III		19	
1355_LMG_15090	CC10	unknown	Unknown	Ib		8	
1356_LMG_15091	N/D	unknown	Unknown	IV			
1357_LMG_15092	CC1	unknown	Unknown	II		2	
1358_LMG_15093	CC19	unknown	Unknown	V		110	
1359_LMG_15094	CC17	unknown	Unknown	III		17	
1360_LMG_15095	CC17	unknown	Unknown	III		17	
1373_MRI_Z1_012	CC1	animal	Unknown	IV		2	
1374_MRI_Z1_022	CC19	animal	Unknown	NT		121	
1375_MRI_Z1_023	N/D	animal	Unknown	Ia		103	
1376_MRI_Z1_024	CC23	animal	Unknown	Ia		23	
1377_MRI_Z1_025	CC1	animal	Unknown	V		1	
1378_MRI_Z1_035	CC23	animal	Unknown	Ia		88	
1379_MRI_Z1_038	CC10	animal	Unknown	NT		296	
1380_MRI_Z1_039	N/D	animal	Unknown	Ia			
1381_MRI_Z1_048	N/D	animal	Unknown	V			
1382_MRI_Z1_049	N/D	animal	Unknown	III			
1383_MRI_Z1_198	CC10	animal	Unknown	Ib		12	
1384_MRI_Z1_199	CC23	animal	Unknown	Ia		23	
1385_MRI_Z1_200	CC23	animal	Unknown	Ia		23	
1386_MRI_Z1_201	CC23	animal	Unknown	NT		23	
1387_MRI_Z1_202	CC23	animal	Unknown	Ia		23	
1388_MRI_Z1_203	CC23	animal	Unknown	Ia		23	
1389_MRI_Z1_204	CC23	animal	Unknown	Ia		23	
1390_MRI_Z1_205	CC1	animal	Unknown	V		1	
1391_MRI_Z1_206	CC10	animal	Unknown	NT		8	
1392_MRI_Z1_209	N/D	animal	Unknown	NT			
1393_MRI_Z1_211	CC1	animal	Unknown	NT		1	
1394_MRI_Z1_212	CC1	animal	Unknown	NT		1	
1395_MRI_Z1_214	CC17	animal	Unknown	NT		591	
1396_MRI_Z1_216	CC17	animal	Unknown	NT		591	
1397_MRI_Z1_217	N/D	animal	Unknown	NT			
1418_SS1014	CC6	animal	Unknown	NT		6	
1419_SS1218	N/D	animal	Unknown	NT		261	
1420_SS1219	N/D	animal	Unknown	NT		260	
1421_STIR_CD_01	CC6	animal	Unknown	Ia		7	
1422_STIR_CD_07	N/D	animal	Unknown	Ib			
1423_STIR_CD_09	N/D	animal	Unknown	Ib		260	
1424_STIR_CD_13	N/D	animal	Unknown	Ib			
1425_STIR_CD_14	CC10	animal	Unknown	III		491	
1426_STIR_CD_17	N/D	animal	Unknown	Ib			
1427_STIR_CD_21	CC6	animal	Unknown	Ia		7	
1428_STIR_CD_22	CC6	animal	Unknown	Ia		7	
1429_STIR_CD_23	CC6	animal	Unknown	Ia		7	
1430_STIR_CD_24	CC6	animal	Unknown	Ia		7	
1431_STIR_CD_25	CC10	animal	Unknown	III		283	
1432_STIR_CD_26	CC6	animal	Unknown	Ia		500	
1433_STIR_CD_27	CC6	animal	Unknown	Ia		7	

1434_STIR_CD_28	CC6	animal	Unknown	Ia		500	
1435_STIR_CD_29	N/D	animal	Unknown	Ib		261	
1445_ZQ0910	CC6	animal	Unknown	Ia		7	
1446_FSL_S3_105	N/D	animal	Unknown	III			
1447_FSL_S3_277	N/D	animal	Unknown	III			
1455_MRI_Z1_219	CC10	animal	Unknown	II		590	
1456_ENC06	N/D	animal	Unknown	Ia			
1457_FNA07	CC6	animal	Unknown	Ia		7	
1458_FPrA02	CC6	animal	Unknown	Ia		7	
184_SA20_06	N/D	animal	Brazil	Ib	2006	553	
709_MRI_Z1_213	CC17	animal	Italy	NT	2004	591	
710_MRI_Z1_215	CC1	animal	Italy	IV	2004	589	
711_MRI_Z1_218	CC10	animal	Italy	II	2005	590	
745_ILRI112	N/D	animal	Kenya	NT			
763_ILRI005	N/D	animal	Unknown	NT	2004	609	
ERR1624734	CC19	unknown	Netherlands	III		19	
ERR1624739	CC23	unknown	Netherlands	Ia		23	
ERR1624740	CC17	unknown	Netherlands	III		17	
ERR1624750	CC19	unknown	Netherlands	III		19	
ERR1624754	CC19	unknown	Netherlands	II		19	
ERR1624766	CC17	unknown	Netherlands	III		17	
ERR1624768	CC19	unknown	Netherlands	III		19	
ERR1624770	CC17	unknown	Netherlands	III		17	
ERR1624773	CC6	unknown	Netherlands	Ia		7	
ERR1624786	CC19	unknown	Netherlands	III		19	
ERR1624791	CC19	unknown	Netherlands	III		19	
ERR1624792	CC19	unknown	Netherlands	III		19	
ERR1624793	CC10	unknown	Netherlands	Ib		12	
ERR1624794	CC19	unknown	Netherlands	III		19	
ERR1624797	CC19	unknown	Netherlands	III		19	
ERR1624801	CC10	unknown	Netherlands	Ib		10	
ERR1624802	CC17	unknown	Netherlands	III		17	
ERR1624807	CC17	unknown	Netherlands	III		17	
ERR1624811	CC10	unknown	Netherlands	Ib		12	
ERR1624823	CC6	unknown	Netherlands	Ia		7	
ERR1624827	CC19	unknown	Netherlands	III		19	
ERR1624830	CC6	unknown	Netherlands	Ia		7	
ERR1624831	CC23	unknown	Netherlands	Ia		23	
ERR1624832	CC10	unknown	Netherlands	Ib		12	
ERR1624836	CC6	unknown	Netherlands	Ia		7	
ERR1624848	CC23	unknown	Netherlands	Ia		23	
ERR1624853	CC10	unknown	Netherlands	Ib		9	
ERR1624867	CC19	unknown	Netherlands	III		19	
ERR1624868	CC19	unknown	Netherlands	III		19	
ERR1624871	CC17	unknown	Netherlands	III		17	
ERR1624884	N/D	unknown	Netherlands	III			
ERR1624887	CC19	unknown	Netherlands	III		19	
ERR1624889	CC19	unknown	Netherlands	III		19	
ERR1624894	CC19	unknown	Netherlands	III		19	
ERR1624901	CC17	unknown	Netherlands	III		17	
ERR1624904	CC19	unknown	Netherlands	III		19	
ERR1624918	CC17	unknown	Netherlands	III		17	
ERR1624920	CC10	unknown	Netherlands	Ib		12	
ERR1624922	CC23	unknown	Netherlands	III		23	
ERR1624924	CC17	unknown	Netherlands	III		17	
ERR1624927	CC17	unknown	Netherlands	III		17	
ERR1624928	CC23	unknown	Netherlands	Ia		23	
ERR1624930	CC19	unknown	Netherlands	II		28	
ERR1624939	CC17	unknown	Netherlands	III		17	
ERR1624947	CC19	unknown	Netherlands	III		19	
ERR1624952	N/D	unknown	Netherlands	Ia			
ERR1624955	N/D	unknown	Netherlands	Ia			

ERR1624958	CC17	unknown	Netherlands	III		17	
ERR1624959	CC17	unknown	Netherlands	III		17	
ERR1624964	CC10	unknown	Netherlands	Ib		8	
ERR1624970	CC23	unknown	Netherlands	Ia		23	
ERR1624972	CC10	unknown	Netherlands	Ib		12	
ERR1624973	CC17	unknown	Netherlands	III		17	
ERR1624979	CC17	unknown	Netherlands	III		17	
ERR1624980	CC19	unknown	Netherlands	III		19	
ERR1624986	CC19	unknown	Netherlands	III		547	
ERR1624987	CC10	unknown	Netherlands	Ib		9	
ERR1624996	CC19	unknown	Netherlands	III		19	
ERR1624998	CC23	unknown	Netherlands	Ia		23	
ERR1625003	CC23	unknown	Netherlands	Ia		23	
ERR1625005	CC10	unknown	Netherlands	Ib		10	
ERR1625010	CC17	unknown	Netherlands	III		17	
ERR1625015	CC17	unknown	Netherlands	III		17	
ERR1625019	CC23	unknown	Netherlands	Ia		23	
ERR1625020	CC6	unknown	Netherlands	Ib		6	
ERR1625024	CC19	unknown	Netherlands	III		19	
ERR1625026	CC19	unknown	Netherlands	III		182	
ERR1625028	CC17	unknown	Netherlands	III		17	
ERR1625035	CC10	unknown	Netherlands	Ib		12	
ERR1625036	CC23	unknown	Netherlands	Ia		24	
ERR1625037	CC10	unknown	Netherlands	Ib		12	
ERR1625038	CC19	unknown	Netherlands	III		19	
ERR1625042	CC1	unknown	Netherlands	II		1	
ERR1625049	CC17	unknown	Netherlands	III		17	
ERR1625053	CC17	unknown	Netherlands	III		148	
ERR1625055	CC17	unknown	Netherlands	III		17	
ERR1625061	CC10	unknown	Netherlands	Ib		12	
ERR1625072	CC17	unknown	Netherlands	III		17	
ERR1625076	N/D	unknown	Netherlands	V		26	
ERR1625087	CC19	unknown	Netherlands	III		19	
ERR1625094	CC10	unknown	Netherlands	Ib		8	
ERR1625096	CC17	unknown	Netherlands	III		17	
ERR1625099	CC17	unknown	Netherlands	III		17	
ERR1625105	CC17	unknown	Netherlands	III		17	
ERR1625108	CC10	unknown	Netherlands	Ib		8	
ERR1625117	CC19	unknown	Netherlands	III		19	
ERR1625125	CC17	unknown	Netherlands	III		17	
ERR1625127	CC19	unknown	Netherlands	III		19	
ERR1625129	CC23	unknown	Netherlands	Ia		23	
ERR1625131	CC19	unknown	Netherlands	III		19	
ERR1625140	CC23	unknown	Netherlands	Ia		23	
ERR1625144	CC1	unknown	Netherlands	V		1	
ERR1625146	CC10	unknown	Netherlands	Ib		10	
ERR1625174	CC23	unknown	Netherlands	Ia		23	
ERR1625175	N/D	unknown	Netherlands	Ia			
ERR1625181	CC10	unknown	Netherlands	Ib		10	
ERR1625186	CC19	unknown	Netherlands	III		19	
ERR1625192	CC19	unknown	Netherlands	III		19	
ERR1625194	CC23	unknown	Netherlands	Ia		23	
ERR1625195	CC17	unknown	Netherlands	III		17	
ERR1625202	CC10	unknown	Netherlands	Ib		12	
ERR1625211	CC19	unknown	Netherlands	III		19	
ERR1625216	CC23	unknown	Netherlands	Ia		23	
ERR1625221	CC23	unknown	Netherlands	III		23	
ERR1625223	CC17	unknown	Netherlands	III		17	
ERR1625226	N/D	unknown	Netherlands	III			
ERR1625229	CC17	unknown	Netherlands	III		17	
ERR1625231	CC23	unknown	Netherlands	Ia		23	
ERR1625233	CC17	unknown	Netherlands	III		17	

ERR1625234	CC23	unknown	Netherlands	Ia			23	
ERR1625235	CC23	unknown	Netherlands	Ia			23	
ERR1625237	N/D	unknown	Netherlands	III				
ERR1625240	CC19	unknown	Netherlands	III			19	
ERR1625243	CC17	unknown	Netherlands	III			17	
ERR1625257	CC17	unknown	Netherlands	III			17	
ERR1625260	CC17	unknown	Netherlands	III			17	
ERR1625269	CC23	unknown	Netherlands	Ia			24	
ERR1625271	CC19	unknown	Netherlands	III			19	
ERR1625278	CC17	unknown	Netherlands	III			17	
ERR1625282	CC1	unknown	Netherlands	IV			459	
ERR1625283	CC17	unknown	Netherlands	III			17	
ERR1625284	CC17	unknown	Netherlands	III			17	
ERR1625285	CC10	unknown	Netherlands	Ib			12	
ERR1625287	CC19	unknown	Netherlands	III			328	
ERR1625288	CC17	unknown	Netherlands	III			17	
ERR1625290	CC17	unknown	Netherlands	III			17	
ERR1625291	CC17	unknown	Netherlands	III			17	
ERR1625294	CC17	unknown	Netherlands	III			17	
ERR1625297	CC17	unknown	Netherlands	III			148	
ERR1625301	CC19	unknown	Netherlands	III			19	
ERR1625302	CC1	unknown	Netherlands	V			1	
ERR1625307	CC17	unknown	Netherlands	III			17	
ERR1625313	CC19	unknown	Netherlands	III			19	
ERR1625331	CC17	unknown	Netherlands	III			17	
ERR1625332	CC19	unknown	Netherlands	III			19	
ERR1625333	CC10	unknown	Netherlands	Ib			8	
ERR1625339	CC1	unknown	Netherlands	V			1	
ERR1625352	CC19	unknown	Netherlands	III			19	
ERR1625357	CC1	unknown	Netherlands	V			1	
ERR1625361	CC23	unknown	Netherlands	Ia			23	
ERR1625372	CC17	unknown	Netherlands	III			17	
ERR1625376	CC19	unknown	Netherlands	III			19	
ERR1625393	CC17	unknown	Netherlands	III			17	
ERR1625395	N/D	unknown	Netherlands	II				
ERR1625397	CC17	unknown	Netherlands	III			17	
ERR1625402	CC17	unknown	Netherlands	III			17	
ERR1625406	CC10	unknown	Netherlands	Ib			10	
ERR1625408	N/D	unknown	Netherlands	III				
ERR1625414	CC19	unknown	Netherlands	II			19	
ERR1625451	CC6	unknown	Netherlands	V			7	
ERR1625455	CC17	unknown	Netherlands	III			17	
ERR1625456	CC23	unknown	Netherlands	Ia			23	
ERR1625464	CC17	unknown	Netherlands	III			17	
ERR1625483	CC23	unknown	Netherlands	Ia			23	
ERR1659687	CC17	unknown	Netherlands	III			17	
ERR1659690	CC17	unknown	Netherlands	III			17	
ERR1659693	CC23	unknown	Netherlands	Ia			23	
ERR1659694	CC17	unknown	Netherlands	III			17	
ERR1659702	CC10	unknown	Netherlands	Ib			12	
ERR1659704	CC23	unknown	Netherlands	Ia			144	
ERR1659707	CC23	unknown	Netherlands	V			23	
ERR1659712	CC17	unknown	Netherlands	III			17	
ERR1659715	CC19	unknown	Netherlands	III			19	
ERR1659730	CC23	unknown	Netherlands	Ia			23	
ERR1659735	CC10	unknown	Netherlands	Ib			8	
ERR1659738	CC17	unknown	Netherlands	III			17	
ERR1659739	CC17	unknown	Netherlands	III			17	
ERR1659740	N/D	unknown	Netherlands	III				
ERR1659743	CC17	unknown	Netherlands	III			17	
ERR1659749	N/D	unknown	Netherlands	III				
ERR1659754	CC1	unknown	Netherlands	Ib			1	

ERR1659761	CC17	unknown	Netherlands	III		17	
ERR1659775	CC17	unknown	Netherlands	III		17	
ERR1659798	CC17	unknown	Netherlands	III		17	
ERR1659813	CC17	unknown	Netherlands	III		17	
ERR1659816	CC6	unknown	Netherlands	V		7	
ERR1659819	CC23	unknown	Netherlands	Ia		23	
ERR1659823	CC17	unknown	Netherlands	III		17	
ERR1659825	CC23	unknown	Netherlands	Ia		23	
ERR1659830	CC19	unknown	Netherlands	III		19	
ERR1659834	N/D	unknown	Netherlands	V			
ERR1659841	CC19	unknown	Netherlands	II		28	
ERR1659847	N/D	unknown	Netherlands	Ia		103	
ERR1659851	CC23	unknown	Netherlands	Ia		24	
ERR1659867	CC17	unknown	Netherlands	III		17	
ERR1672384	N/D	unknown	Netherlands	III			
ERR1672386	CC10	unknown	Netherlands	Ib		12	
ERR1672388	CC17	unknown	Netherlands	III		17	
ERR1672395	N/D	unknown	Netherlands	III			
ERR1672396	CC17	unknown	Netherlands	III		17	
ERR1672400	CC19	unknown	Netherlands	III		19	
ERR1672407	CC10	unknown	Netherlands	Ib		10	
ERR1672411	CC23	unknown	Netherlands	Ia		23	
ERR1672414	CC1	unknown	Netherlands	V		1	
ERR1672417	CC19	unknown	Netherlands	III		19	
ERR1672424	N/D	unknown	Netherlands	III			
ERR1672425	CC17	unknown	Netherlands	III		17	
ERR1672426	CC17	unknown	Netherlands	III		17	
ERR1672430	CC17	unknown	Netherlands	III		17	
ERR1672431	CC19	unknown	Netherlands	III		19	
ERR1672432	CC17	unknown	Netherlands	III		17	
ERR1672433	CC17	unknown	Netherlands	III		17	
ERR1672447	CC23	unknown	Netherlands	Ia		24	
ERR1672450	CC23	unknown	Netherlands	Ia		23	
ERR1672452	N/D	unknown	Netherlands	III			
ERR1672455	CC23	unknown	Netherlands	Ia		23	
ERR1672456	CC23	unknown	Netherlands	Ia		24	
ERR1672459	N/D	unknown	Netherlands	Ia			
ERR1672464	CC6	unknown	Netherlands	III		7	
ERR1672472	CC17	unknown	Netherlands	III		17	
ERR1672473	CC17	unknown	Netherlands	III		17	
ERR1672479	CC17	unknown	Netherlands	III		17	
ERR1672481	CC1	unknown	Netherlands	IV		2	
ERR1672487	CC17	unknown	Netherlands	III		17	
ERR1672501	CC23	unknown	Netherlands	Ia		23	
ERR1672503	CC19	unknown	Netherlands	III		19	
ERR1672504	CC23	unknown	Netherlands	Ia		23	
ERR1672509	CC17	unknown	Netherlands	III		17	
ERR1672511	CC19	unknown	Netherlands	III		19	
ERR1672512	CC19	unknown	Netherlands	II		19	
ERR1672526	CC17	unknown	Netherlands	III		17	
ERR1672528	CC17	unknown	Netherlands	III		17	
ERR1672529	CC19	unknown	Netherlands	III		19	
ERR1672530	CC19	unknown	Netherlands	III		19	
ERR1672545	CC17	unknown	Netherlands	III		17	
ERR1672557	CC17	unknown	Netherlands	III		17	
ERR1672561	CC19	unknown	Netherlands	III		19	
ERR1672563	CC17	unknown	Netherlands	III		17	
ERR1672564	CC17	unknown	Netherlands	III		17	
ERR1672574	CC17	unknown	Netherlands	III		17	
ERR1672578	CC17	unknown	Netherlands	III		17	
ERR1672583	CC10	unknown	Netherlands	Ib		8	
ERR1672586	N/D	unknown	Netherlands	III			

ERR1672600	CC17	unknown	Netherlands	III			17	
ERR1672601	CC19	unknown	Netherlands	III			19	
ERR1672607	CC19	unknown	Netherlands	III			19	
ERR1672613	CC10	unknown	Netherlands	Ib			12	
ERR1672621	CC23	unknown	Netherlands	Ia			23	
ERR1672623	CC19	unknown	Netherlands	III			233	
ERR1672628	CC23	unknown	Netherlands	Ia			144	
ERR1672631	CC6	unknown	Netherlands	Ia			7	
ERR1672633	CC17	unknown	Netherlands	III			17	
ERR1672635	CC23	unknown	Netherlands	Ia			23	
ERR1672636	CC19	unknown	Netherlands	V			19	
ERR1672654	CC10	unknown	Netherlands	Ib			12	
ERR1672660	CC17	unknown	Netherlands	III			17	
ERR1672663	CC17	unknown	Netherlands	IV			291	
ERR1672671	CC23	unknown	Netherlands	Ia			23	
ERR1672672	CC17	unknown	Netherlands	III			17	
ERR1672692	N/D	unknown	Netherlands	Ia				
ERR1672694	CC17	unknown	Netherlands	III			17	
ERR1672708	CC17	unknown	Netherlands	III			17	
ERR1672711	CC1	unknown	Netherlands	V			1	
ERR1672712	CC19	unknown	Netherlands	III			19	
ERR1672718	CC17	unknown	Netherlands	III			148	
ERR1672725	CC17	unknown	Netherlands	III			17	
ERR1672728	CC19	unknown	Netherlands	V			110	
ERR1672735	CC23	unknown	Netherlands	Ia			23	
ERR1672739	CC17	unknown	Netherlands	III			17	
ERR1672748	CC17	unknown	Netherlands	III			17	
ERR1672751	CC17	unknown	Netherlands	III			17	
ERR1672753	CC17	unknown	Netherlands	III			17	
ERR1672758	CC17	unknown	Netherlands	III			17	
ERR1672771	CC23	unknown	Netherlands	Ia			24	
ERR1672777	CC17	unknown	Netherlands	III			17	
ERR1672782	CC17	unknown	Netherlands	III			17	
ERR1672784	CC23	unknown	Netherlands	Ia			144	
ERR1672794	CC17	unknown	Netherlands	III			17	
ERR1672797	CC17	unknown	Netherlands	III			17	
ERR1672798	CC23	unknown	Netherlands	Ia			23	
ERR1672801	N/D	unknown	Netherlands	III				
ERR1672803	CC23	unknown	Netherlands	Ia			23	
ERR1672808	CC17	unknown	Netherlands	III			17	
ERR1672812	CC23	unknown	Netherlands	Ia			24	
ERR1672821	CC17	unknown	Netherlands	III			17	
ERR1672834	CC17	unknown	Netherlands	III			17	
ERR1672836	CC23	unknown	Netherlands	Ia			144	
ERR1672839	CC19	unknown	Netherlands	V			110	
ERR1672840	CC17	unknown	Netherlands	III			17	
ERR1672842	CC23	unknown	Netherlands	Ia			23	
ERR1672843	CC19	unknown	Netherlands	II			28	
ERR1672847	N/D	unknown	Netherlands	III				
ERR1672849	CC23	unknown	Netherlands	Ia			23	
ERR1672850	CC23	unknown	Netherlands	Ia			23	
ERR1672851	CC23	unknown	Netherlands	Ia			23	
ERR1672854	N/D	unknown	Netherlands	III				
ERR1672856	N/D	unknown	Netherlands	III				
ERR1672860	CC23	unknown	Netherlands	Ia			23	
ERR1672861	CC17	unknown	Netherlands	III			17	
ERR1672873	CC23	unknown	Netherlands	Ia			23	
ERR1672874	CC17	unknown	Netherlands	III			17	
ERR1672882	CC17	unknown	Netherlands	III			17	
ERR1672883	CC17	unknown	Netherlands	III			17	
ERR1672889	CC1	unknown	Netherlands	V			1	
ERR1672893	CC17	unknown	Netherlands	III			17	

ERR1672907	CC17	unknown	Netherlands	III		17	
ERR1672915	CC23	unknown	Netherlands	Ia		23	
ERR1672924	CC17	unknown	Netherlands	III		17	
ERR1672927	CC17	unknown	Netherlands	III		17	
ERR1672933	CC17	unknown	Netherlands	III		17	
