# Supplementary material for: Pan-GWAS of Streptococcus agalactiae Highlights Lineage-Specific Genes Associated with Virulence and Niche Adaptation
Source: mBio. 2020 Jun 9;11(3):e00728-20. doi: 10.1128/mBio.00728-20 (PMC7373188; doi:10.1128/mBio.00728-20)
Supplement: TABLE S2 [file mBio.00728-20-st002.pdf]

| CC | ST | Brazil | Canada | Germany | Italy | Kenya | Malawi | Netherlands | USA | Unknown |
|---|---|---|---|---|---|---|---|---|---|---|
| CC1 | 1 | 0 | 69 | 0 | 0 | 87 | 1 | 9 | 95 | 6 |
| | 14 | 0 | 0 | 0 | 0 | 1 | 0 | 0 | 0 | 0 |
| | 153 | 0 | 0 | 0 | 0 | 0 | 0 | 0 | 1 | 0 |
| | 167 | 0 | 0 | 0 | 0 | 1 | 0 | 0 | 0 | 0 |
| | 196 | 0 | 0 | 0 | 0 | 17 | 0 | 0 | 0 | 0 |
| | 2 | 0 | 1 | 0 | 0 | 7 | 0 | 1 | 0 | 2 |
| | 297 | 0 | 0 | 0 | 0 | 0 | 0 | 0 | 1 | 0 |
| | 453 | 0 | 1 | 0 | 0 | 0 | 0 | 0 | 0 | 0 |
| | 459 | 0 | 0 | 0 | 0 | 0 | 0 | 1 | 0 | 0 |
| | 531 | 0 | 1 | 0 | 0 | 0 | 0 | 0 | 0 | 0 |
| | 589 | 0 | 0 | 0 | 1 | 0 | 0 | 0 | 0 | 0 |
| | 794 | 0 | 0 | 0 | 0 | 1 | 0 | 0 | 0 | 0 |
| | 796 | 0 | 0 | 0 | 0 | 9 | 0 | 0 | 0 | 0 |
| | 799 | 0 | 0 | 0 | 0 | 2 | 0 | 0 | 0 | 0 |
| | 871 | 0 | 1 | 0 | 0 | 0 | 0 | 0 | 0 | 0 |
| | 872 | 0 | 0 | 0 | 0 | 0 | 0 | 0 | 1 | 0 |
| | 873 | 0 | 0 | 0 | 0 | 0 | 0 | 0 | 1 | 0 |
| CC6 | 500 | 0 | 0 | 0 | 0 | 0 | 0 | 0 | 0 | 2 |
| | 6 | 0 | 0 | 0 | 0 | 0 | 0 | 1 | 0 | 1 |
| | 7 | 0 | 0 | 0 | 0 | 0 | 0 | 8 | 0 | 13 |
| CC10 | 10 | 0 | 0 | 0 | 0 | 92 | 6 | 6 | 0 | 0 |
| | 12 | 0 | 0 | 0 | 0 | 2 | 0 | 14 | 0 | 2 |
| | 283 | 0 | 0 | 0 | 0 | 0 | 0 | 0 | 0 | 1 |
| | 296 | 0 | 0 | 0 | 0 | 0 | 0 | 0 | 0 | 1 |
| | 491 | 0 | 0 | 0 | 0 | 0 | 0 | 0 | 0 | 1 |
| | 590 | 0 | 0 | 0 | 1 | 0 | 0 | 0 | 0 | 1 |
| | 787 | 0 | 0 | 0 | 0 | 1 | 0 | 0 | 0 | 0 |
| | 797 | 0 | 0 | 0 | 0 | 1 | 0 | 0 | 0 | 0 |
| | 8 | 0 | 0 | 0 | 0 | 61 | 6 | 6 | 0 | 4 |
| | 804 | 0 | 0 | 0 | 0 | 1 | 0 | 0 | 0 | 0 |
| | 9 | 0 | 0 | 0 | 0 | 0 | 0 | 2 | 0 | 0 |
| CC19 | 110 | 0 | 0 | 0 | 0 | 0 | 0 | 2 | 0 | 1 |
| | 121 | 0 | 0 | 0 | 0 | 0 | 0 | 0 | 0 | 1 |
| | 182 | 0 | 0 | 0 | 0 | 72 | 7 | 1 | 0 | 0 |
| | 19 | 0 | 0 | 0 | 0 | 29 | 6 | 51 | 0 | 4 |
| | 233 | 0 | 0 | 0 | 0 | 0 | 0 | 1 | 0 | 0 |
| | 28 | 0 | 0 | 0 | 0 | 40 | 2 | 3 | 0 | 0 |
| | 327 | 0 | 0 | 0 | 0 | 27 | 27 | 0 | 0 | 0 |
| | 328 | 0 | 0 | 0 | 0 | 9 | 0 | 1 | 0 | 0 |
| | 547 | 0 | 0 | 0 | 0 | 0 | 0 | 1 | 0 | 0 |
| | 793 | 0 | 0 | 0 | 0 | 2 | 0 | 0 | 0 | 0 |
| CC17 | 109 | 0 | 0 | 0 | 0 | 1 | 4 | 0 | 0 | 0 |
| | 147 | 0 | 0 | 0 | 0 | 1 | 0 | 0 | 0 | 0 |
| | 148 | 0 | 2 | 0 | 0 | 0 | 0 | 3 | 0 | 0 |
| | 17 | 0 | 60 | 0 | 0 | 233 | 134 | 109 | 0 | 3 |
| | 22 | 0 | 0 | 0 | 0 | 0 | 0 | 0 | 0 | 2 |
| | 290 | 0 | 1 | 0 | 0 | 0 | 0 | 0 | 0 | 0 |
| | 291 | 0 | 0 | 0 | 0 | 2 | 0 | 1 | 0 | 0 |
| | 31 | 0 | 0 | 0 | 0 | 0 | 0 | 0 | 0 | 1 |
| | 415 | 0 | 0 | 0 | 0 | 0 | 0 | 0 | 0 | 3 |
| | 484 | 0 | 1 | 0 | 0 | 78 | 0 | 0 | 0 | 0 |
| | 490 | 0 | 0 | 0 | 0 | 0 | 0 | 0 | 0 | 1 |
| | 591 | 0 | 0 | 0 | 1 | 0 | 0 | 0 | 0 | 2 |
| | 61 | 0 | 0 | 0 | 0 | 0 | 0 | 0 | 0 | 6 |
| | 67 | 0 | 0 | 0 | 0 | 0 | 0 | 0 | 0 | 2 |
| | 784 | 0 | 0 | 0 | 0 | 1 | 0 | 0 | 0 | 0 |
| | 786 | 0 | 0 | 0 | 0 | 1 | 0 | 0 | 0 | 0 |
| | 788 | 0 | 0 | 0 | 0 | 2 | 0 | 0 | 0 | 0 |
| | 790 | 0 | 0 | 0 | 0 | 2 | 0 | 0 | 0 | 0 |
| | 792 | 0 | 0 | 0 | 0 | 2 | 0 | 0 | 0 | 0 |
| | 795 | 0 | 0 | 0 | 0 | 1 | 0 | 0 | 0 | 0 |
| | 800 | 0 | 0 | 0 | 0 | 1 | 0 | 0 | 0 | 0 |
| | 801 | 0 | 0 | 0 | 0 | 6 | 0 | 0 | 0 | 0 |
| | 866 | 0 | 0 | 0 | 0 | 0 | 8 | 0 | 0 | 0 |
| | 867 | 0 | 0 | 0 | 0 | 0 | 1 | 0 | 0 | 0 |
| | 868 | 0 | 0 | 0 | 0 | 0 | 1 | 0 | 0 | 0 |
| | 869 | 0 | 0 | 0 | 0 | 0 | 1 | 0 | 0 | 0 |
| | 870 | 0 | 0 | 0 | 0 | 0 | 1 | 0 | 0 | 0 |
| | 874 | 0 | 1 | 0 | 0 | 0 | 0 | 0 | 0 | 0 |
| | 95 | 0 | 1 | 0 | 0 | 0 | 0 | 0 | 0 | 0 |
| | 144 | 0 | 0 | 0 | 0 | 1 | 0 | 4 | 0 | 0 |
| | 223 | 0 | 0 | 0 | 0 | 2 | 18 | 0 | 0 | 0 |

| | | | | | | | | | | |
|---|---|---|---|---|---|---|---|---|---|---|
| **CC23** | **23** | 0 | 0 | 1 | 0 | 209 | 60 | 44 | 0 | 8 |
| | **24** | 0 | 0 | 0 | 0 | 7 | 7 | 7 | 0 | 0 |
| | **25** | 0 | 0 | 0 | 0 | 0 | 0 | 0 | 0 | 1 |
| | **498** | 0 | 0 | 0 | 0 | 4 | 0 | 0 | 0 | 0 |
| | **55** | 0 | 0 | 0 | 0 | 1 | 0 | 0 | 0 | 0 |
| | **802** | 0 | 0 | 0 | 0 | 2 | 2 | 0 | 0 | 0 |
| | **88** | 0 | 0 | 0 | 0 | 0 | 0 | 0 | 0 | 2 |
| **Single** | **103** | 0 | 0 | 0 | 0 | 1 | 0 | 1 | 0 | 1 |
| | **26** | 0 | 0 | 0 | 0 | 0 | 0 | 1 | 0 | 0 |
| | **260** | 0 | 0 | 0 | 0 | 0 | 0 | 0 | 0 | 4 |
| | **261** | 0 | 0 | 0 | 0 | 0 | 0 | 0 | 0 | 2 |
| | **3** | 0 | 0 | 0 | 0 | 2 | 0 | 0 | 0 | 0 |
| | **486** | 0 | 0 | 0 | 0 | 2 | 0 | 0 | 0 | 0 |
| | **553** | 1 | 0 | 0 | 0 | 0 | 0 | 0 | 0 | 0 |
| | **609** | 0 | 0 | 0 | 0 | 0 | 0 | 0 | 0 | 1 |
| | **785** | 0 | 0 | 0 | 0 | 1 | 0 | 0 | 0 | 0 |
| | **791** | 0 | 0 | 0 | 0 | 1 | 0 | 0 | 0 | 0 |
| | **798** | 0 | 0 | 0 | 0 | 1 | 0 | 0 | 0 | 0 |
| | **803** | 0 | 0 | 0 | 0 | 1 | 0 | 0 | 0 | 0 |
| | **N/D** | 0 | 2 | 0 | 0 | 7 | 11 | 22 | 0 | 26 |
