# Supplementary figures and images for: Pan-GWAS of Streptococcus agalactiae Highlights Lineage-Specific Genes Associated with Virulence and Niche Adaptation
Source: mBio. 2020 Jun 9;11(3):e00728-20. doi: 10.1128/mBio.00728-20 (PMC7373188; doi:10.1128/mBio.00728-20)

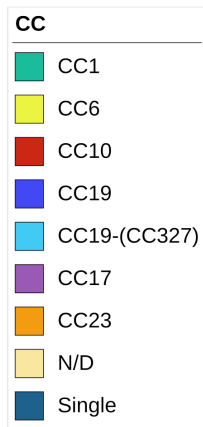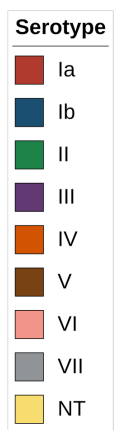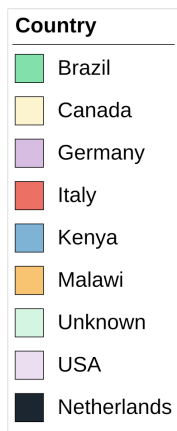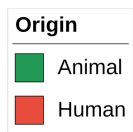

909

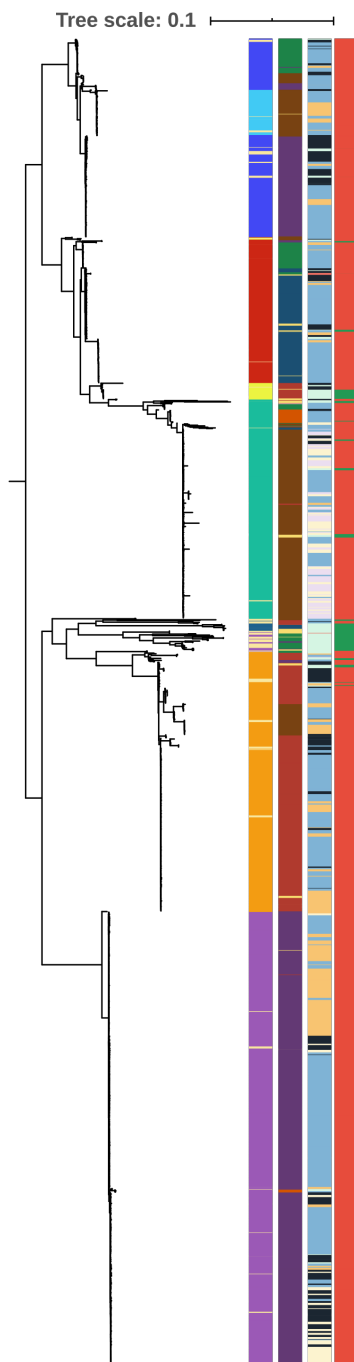

GD201

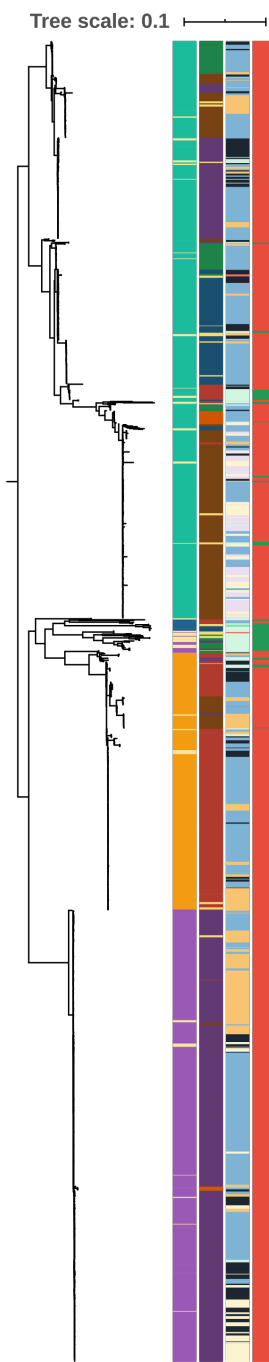

NEM316

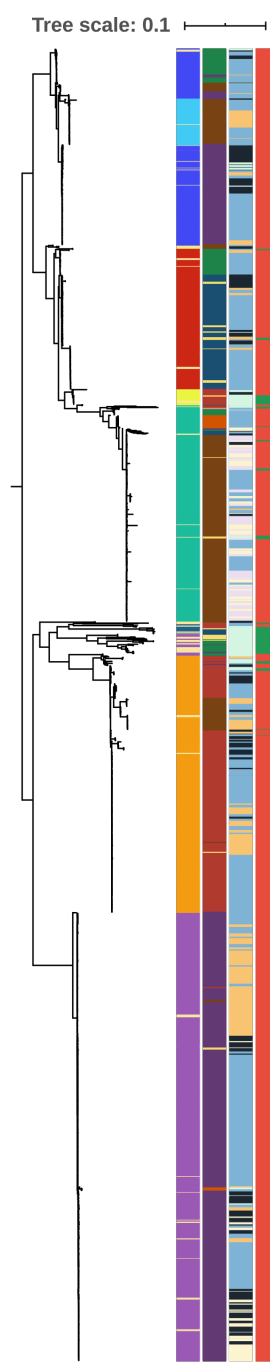

09mas

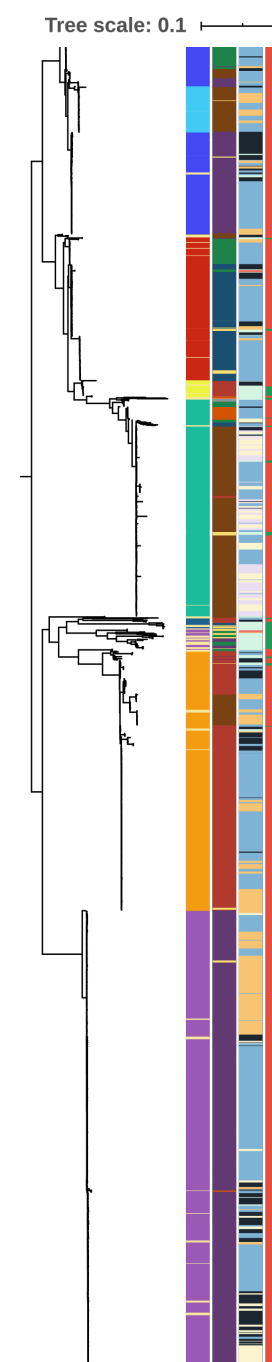

Supplement: FIG S1 [file mBio.00728-20-sf001.pdf]

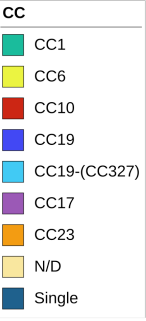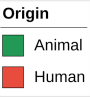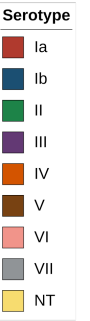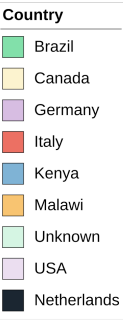

Tree scale: 0.001

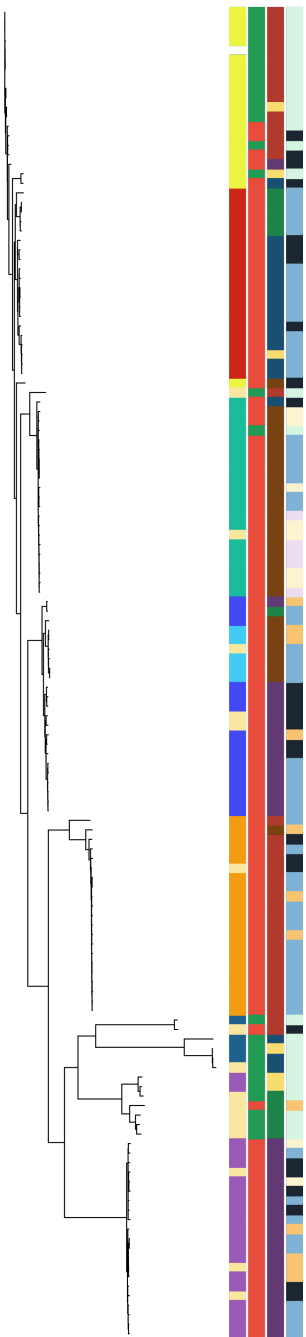

Tree scale: 0.001

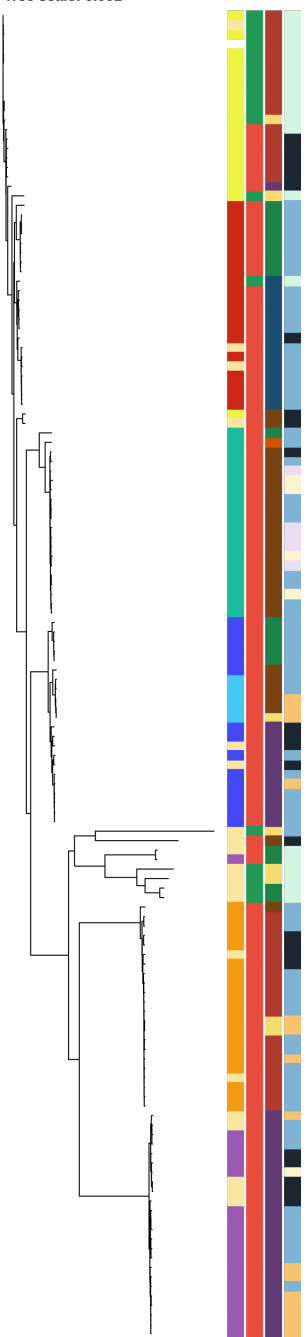

Tree scale: 0.001

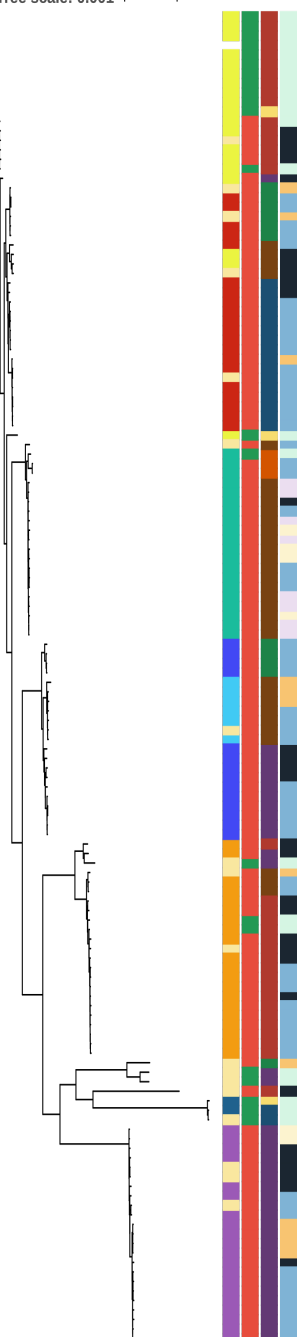

Tree scale: 0.001

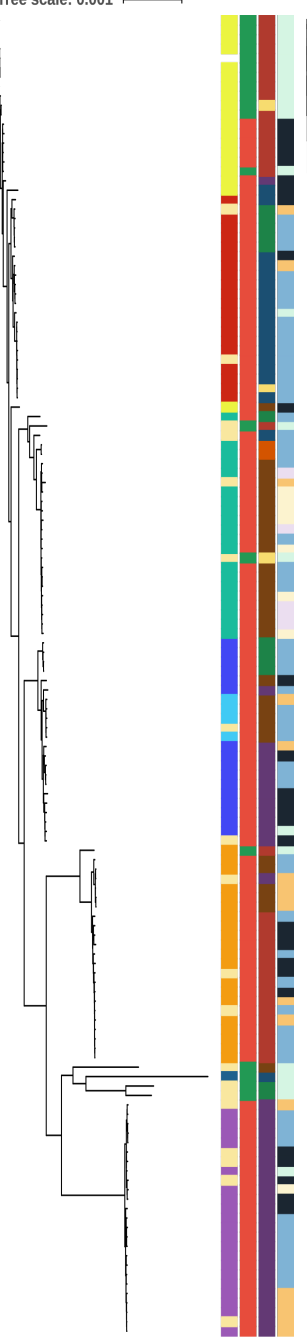

Tree scale: 0.001

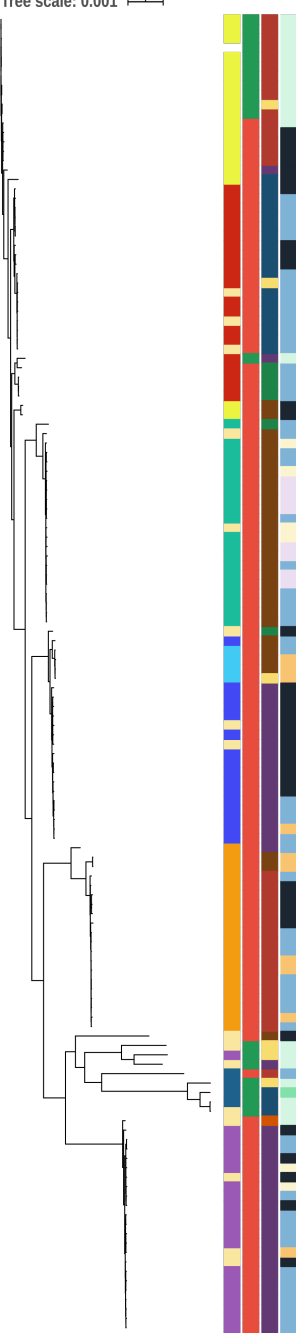

Supplement: FIG S2 [file mBio.00728-20-sf002.pdf]

CC1

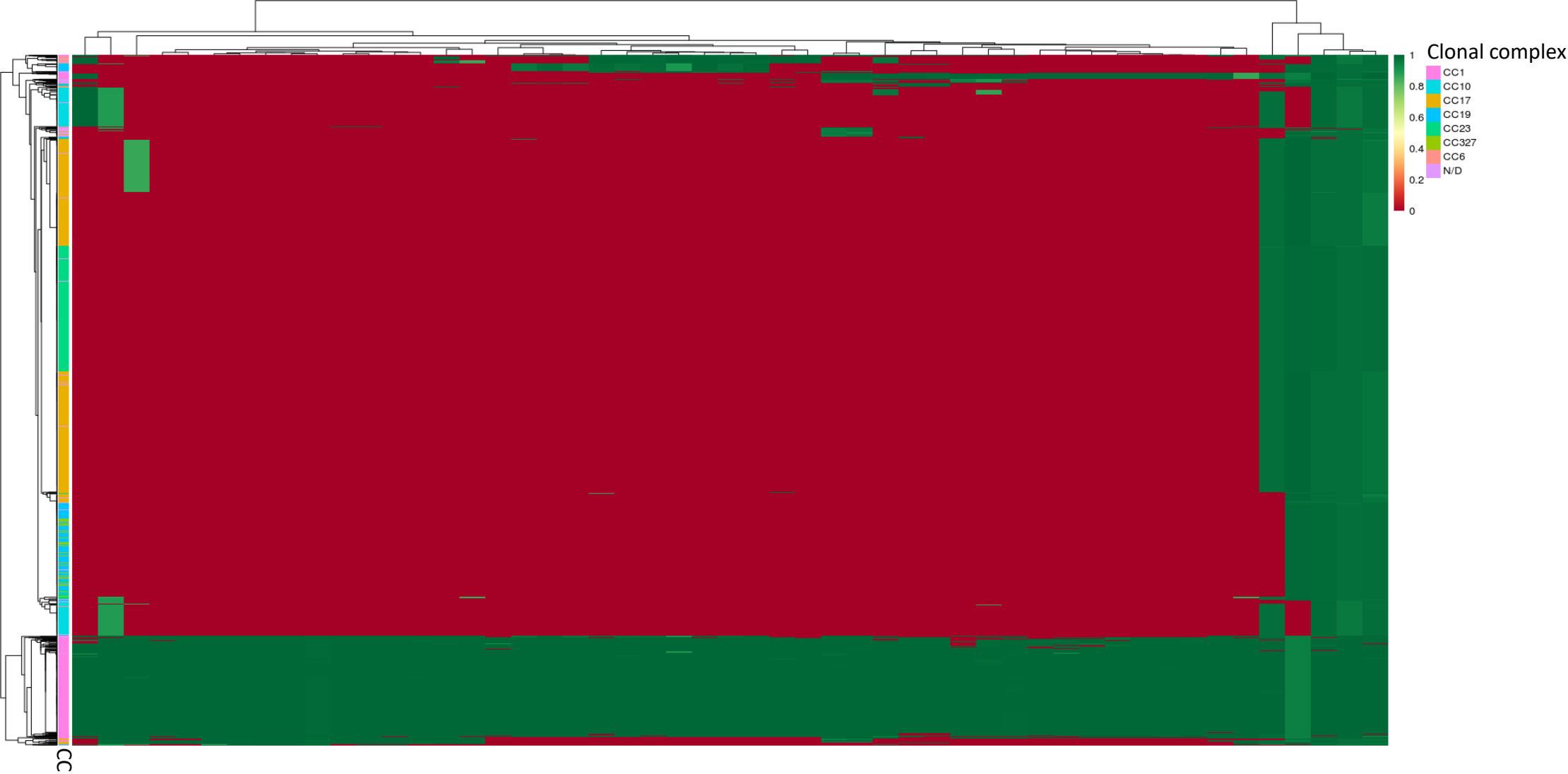

CC10

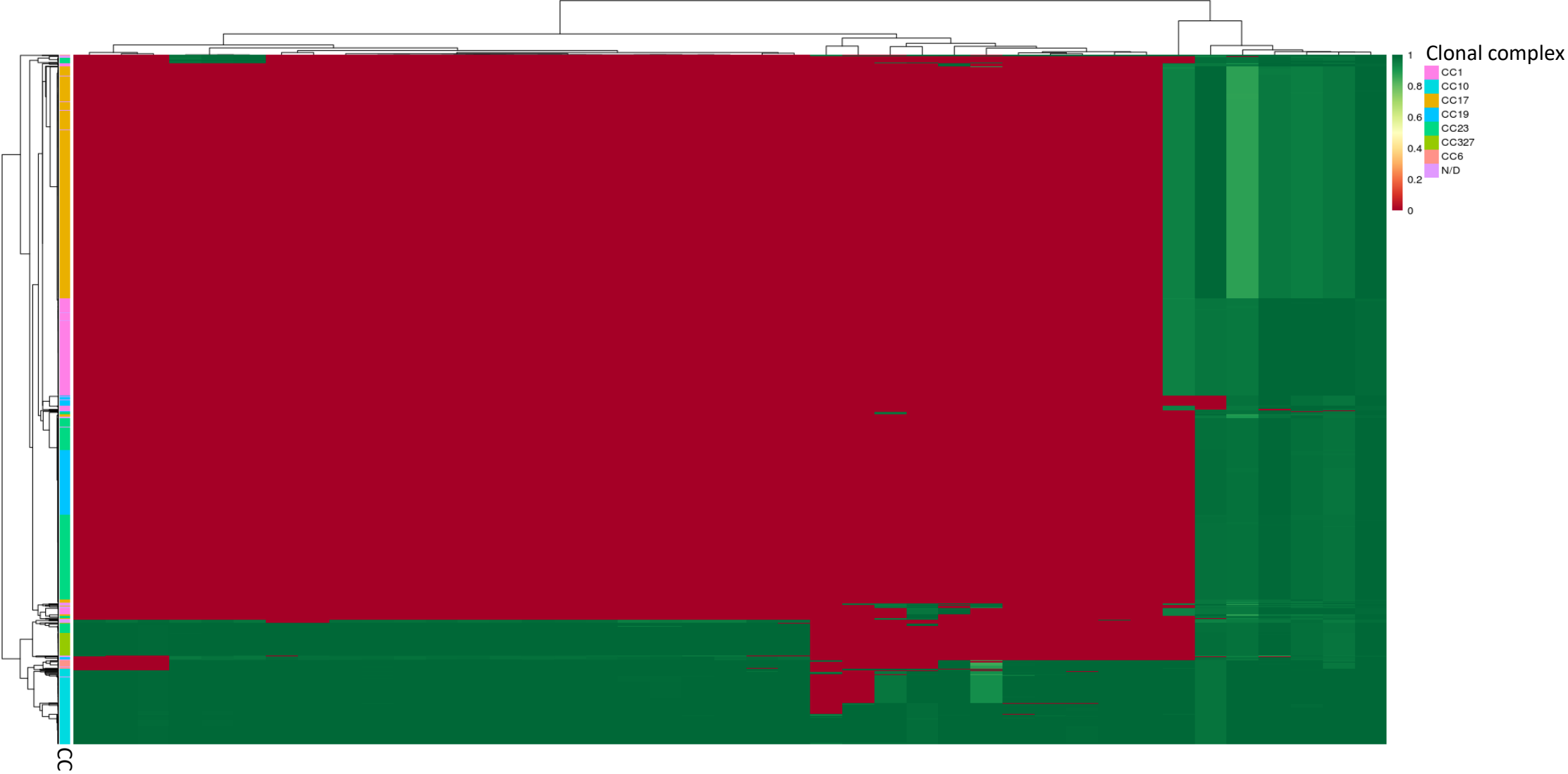

CC19

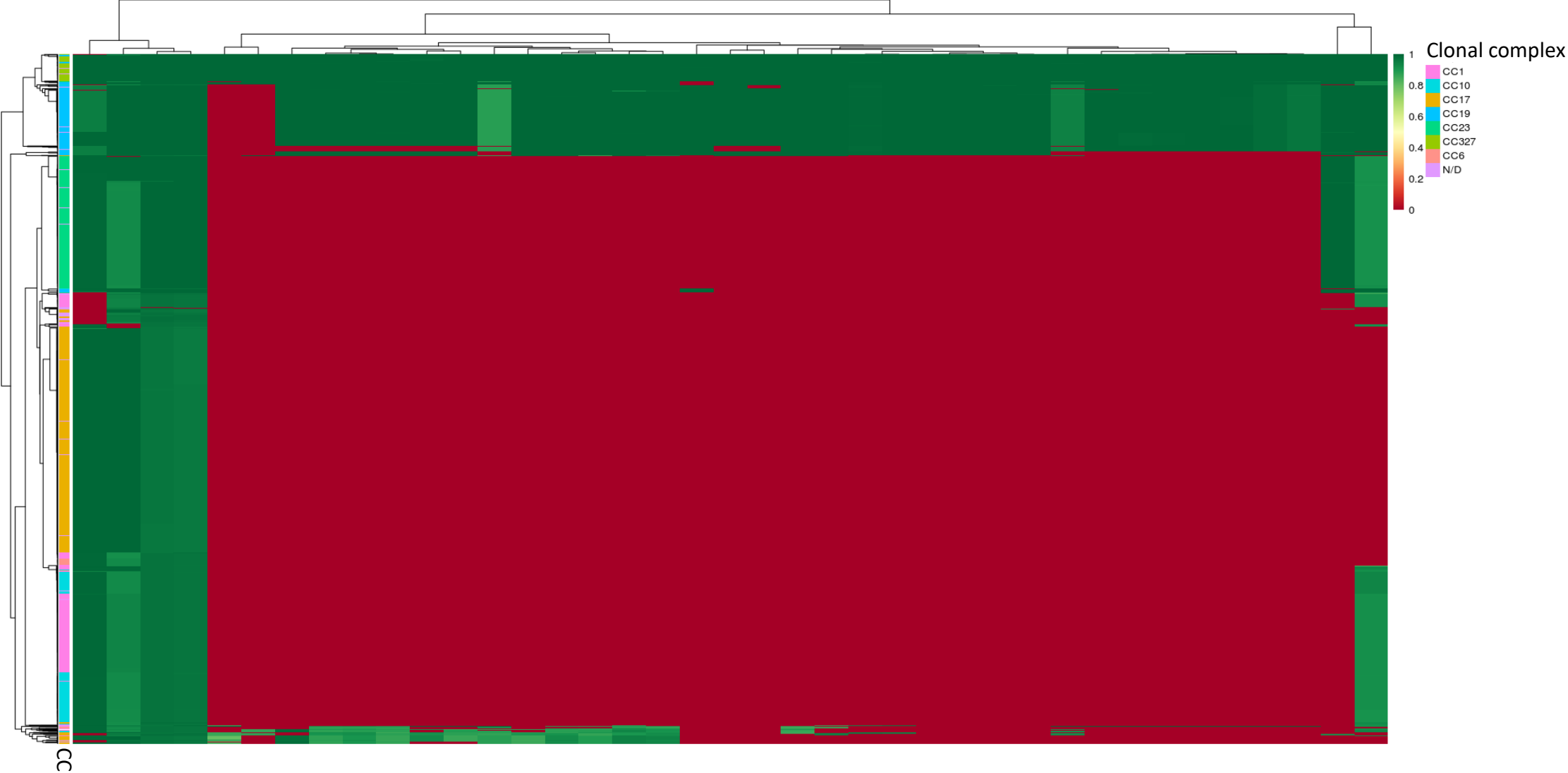

CC17

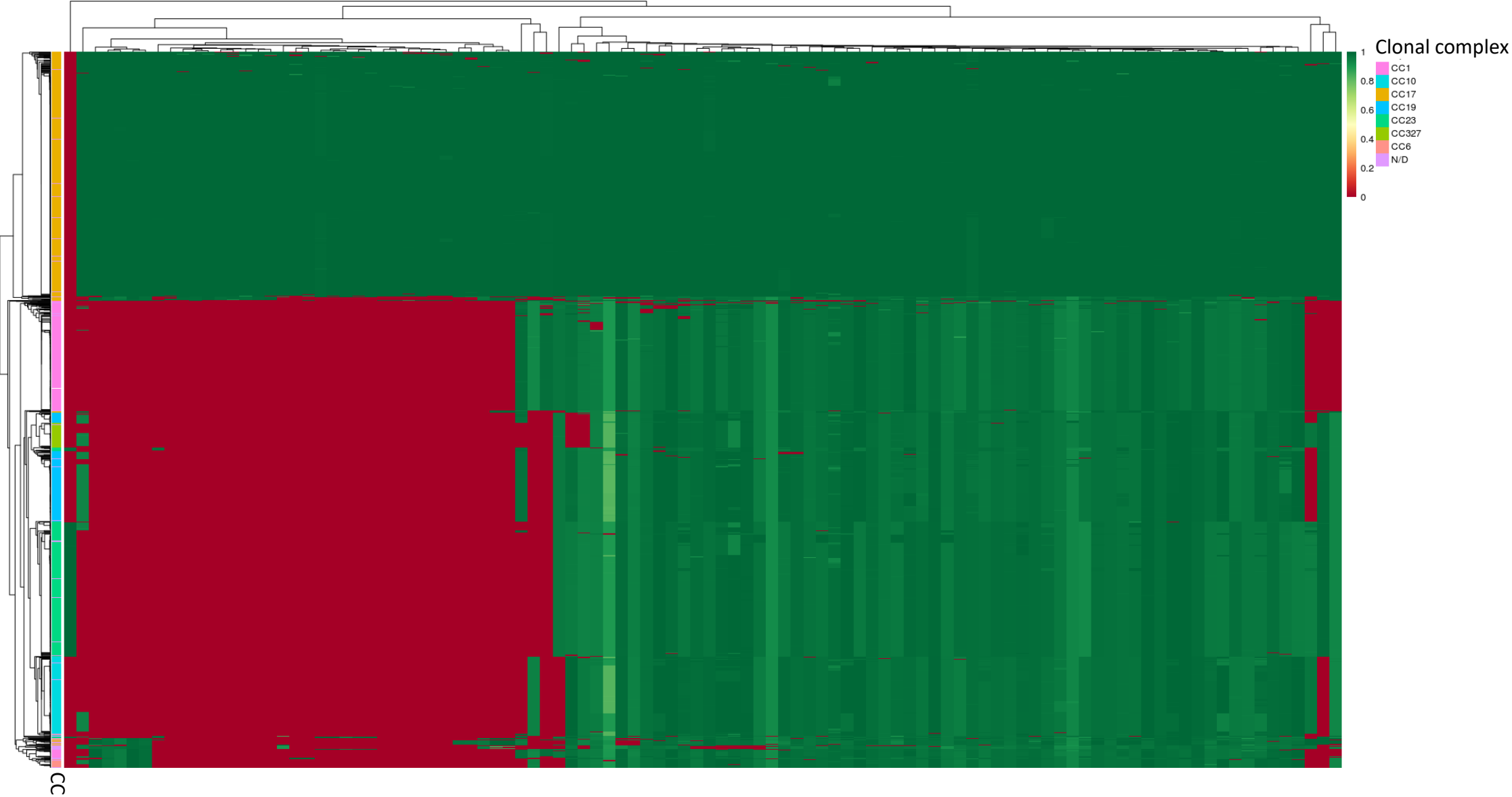

CC23

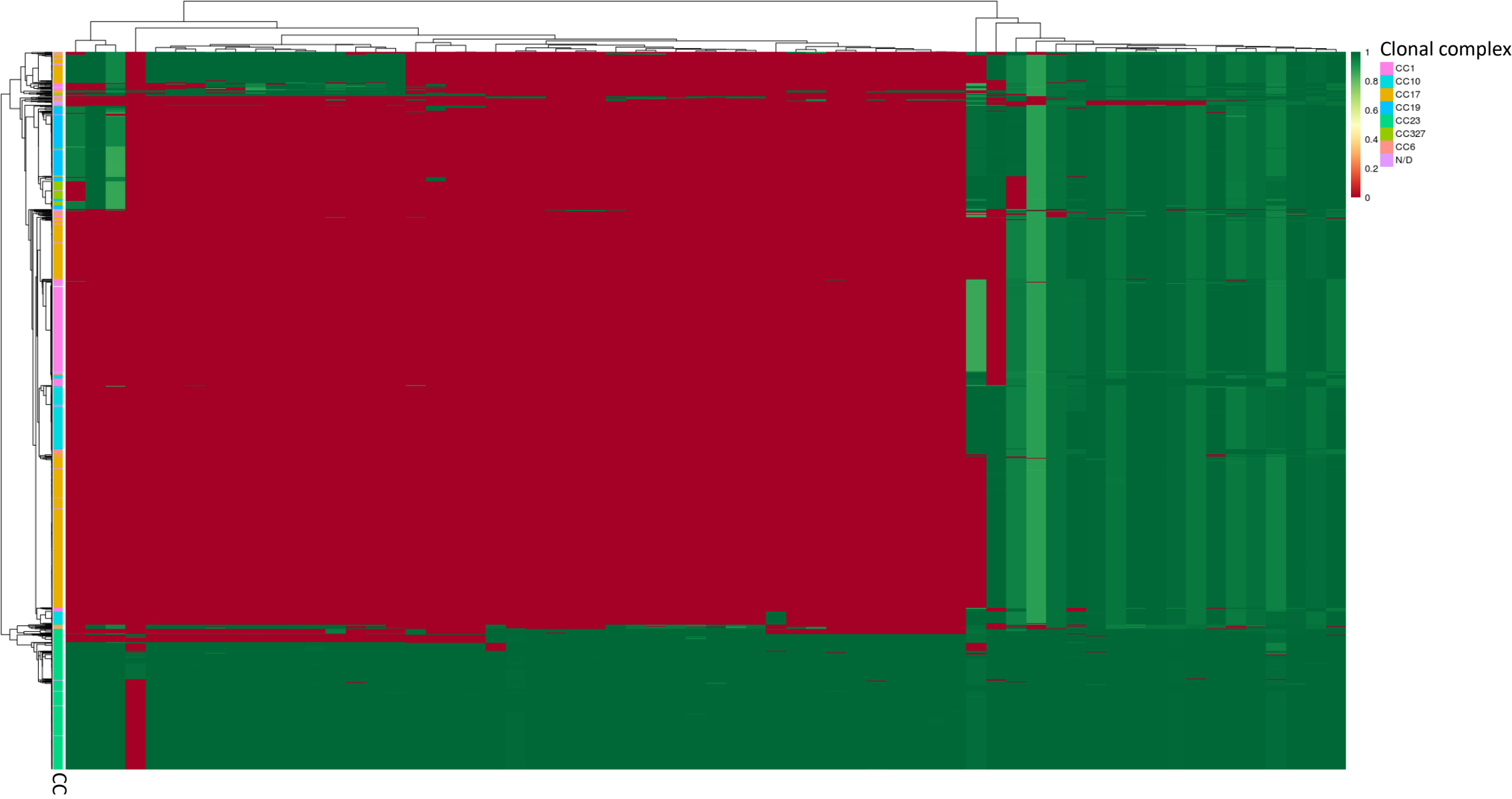

Supplement: FIG S3 [file mBio.00728-20-sf003.pdf]

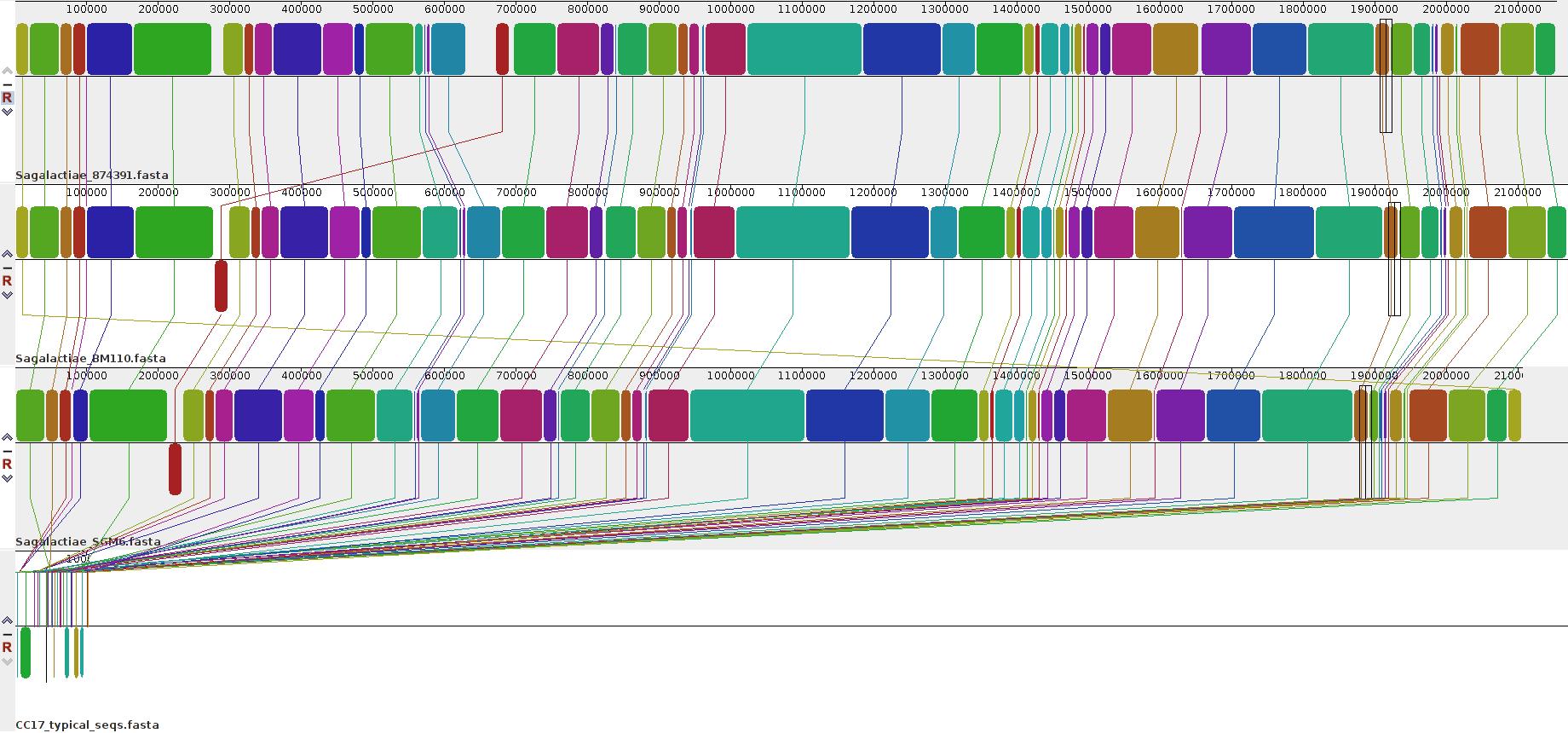

Supplement: FIG S4 [file mBio.00728-20-sf004.jpg]

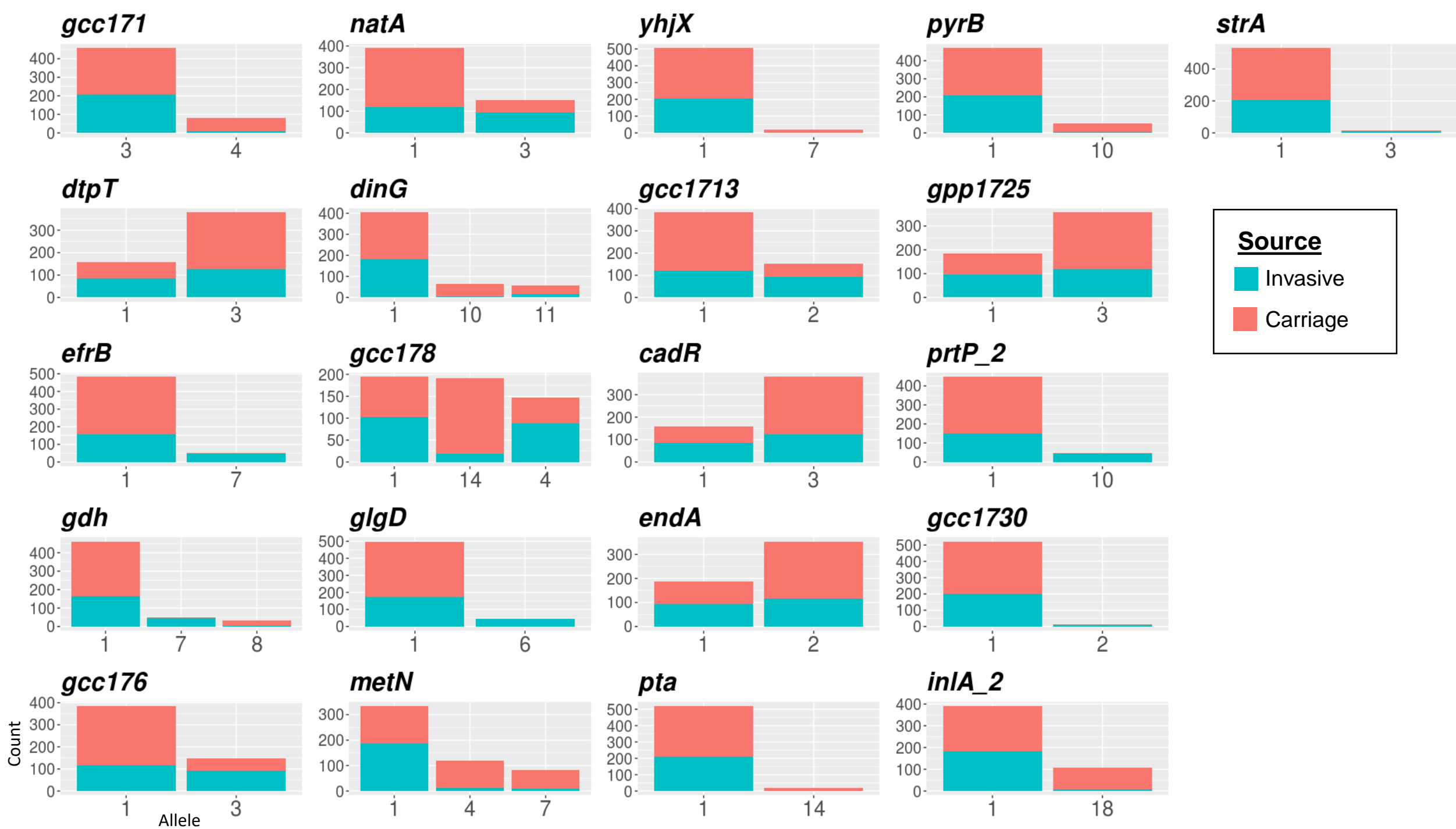

Supplement: FIG S5 [file mBio.00728-20-sf005.pdf]

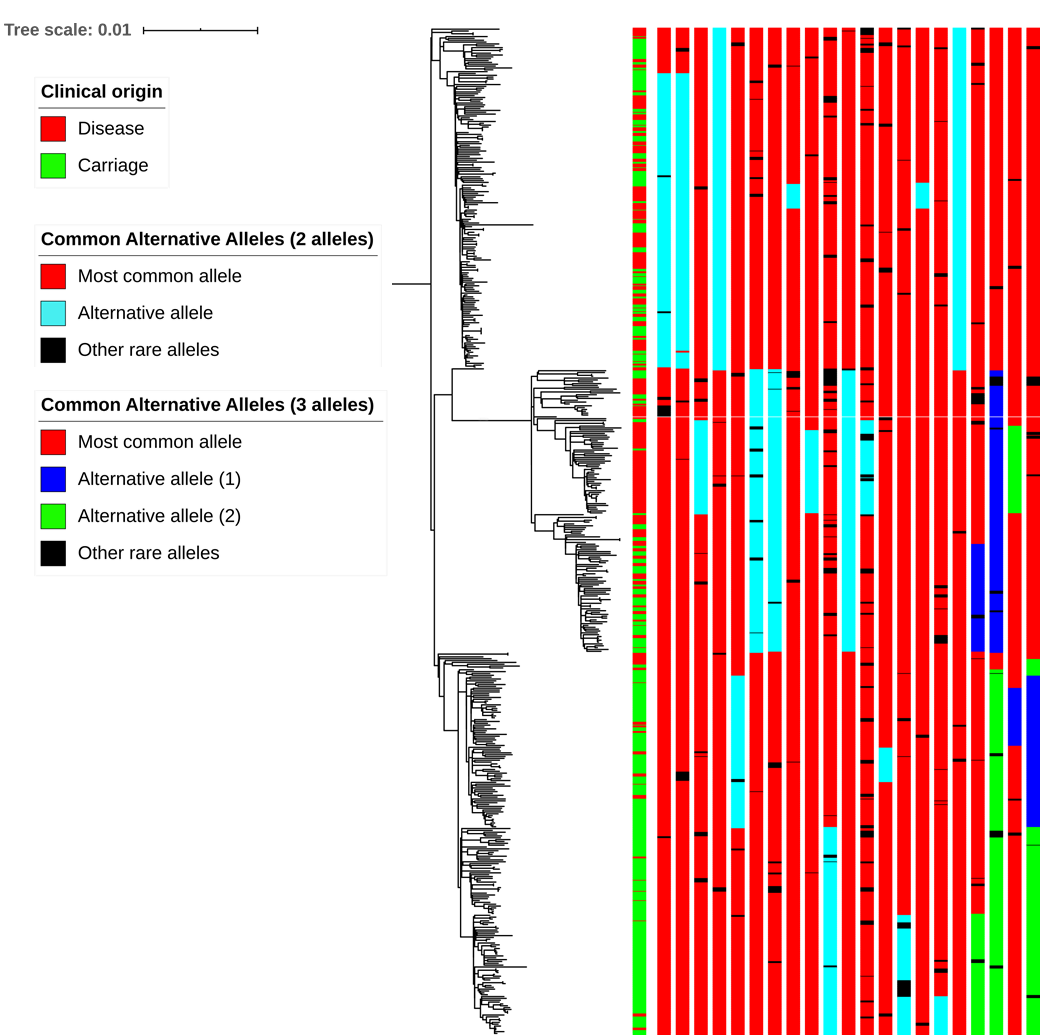

Supplement: FIG S6 [file mBio.00728-20-sf006.tif]
