# Supplementary material for: Pan-GWAS of Streptococcus agalactiae Highlights Lineage-Specific Genes Associated with Virulence and Niche Adaptation
Source: mBio. 2020 Jun 9;11(3):e00728-20. doi: 10.1128/mBio.00728-20 (PMC7373188; doi:10.1128/mBio.00728-20)
Supplement: TABLE S3 [file mBio.00728-20-st003.pdf]

CC1	Gene name	Length (AA)	KEGG #	Annotation (Kegg GO and prokka)	Class	Type of variation
	manX	145	K02744	PTS-Aga-EIIA; PTS system, N-acetylgalactosamine-specific IIA component [EC:2.7.1.-]	env	Gene absent
	gcc11	60		hypothetical protein		Point mutations
	gcc12	190		hypothetical protein		Gene absent
	gcc13	55		hypothetical protein		Gene absent
	gcc14	188		hypothetical protein		Gene absent
	gcc15	374		hypothetical protein		Gene absent
	gcc16	108		hypothetical protein		Gene absent
	gcc17	45		hypothetical protein		Gene absent
	gcc18	531		hypothetical protein		Gene absent
	phd	86	K19165	Antitoxin Phd		Gene absent
	doc	124	K07341	Toxin; death on curing protein		Gene absent
	gcc19	109		hypothetical protein		Gene absent
	gcc110	133		hypothetical protein		Gene absent
	lytG	469		hypothetical protein		Gene absent
	gcc111	176		hypothetical protein		Gene absent
	gcc112	63	K07729	putative transcriptional regulator		Gene absent
	gcc113	134		hypothetical protein		Gene absent
	Tn916	284		hypothetical protein		Gene absent
	xerD	409		hypothetical protein		Gene absent
	gcc114	65		hypothetical protein		Gene absent
	gcc115	273		hypothetical protein		Gene absent
	gcc116	470		hypothetical protein		Gene absent
	gcc117	146		hypothetical protein		Gene absent
	gcc118	71		hypothetical protein		Gene absent
	gcc119	131		hypothetical protein		Gene absent
	gcc120	139		hypothetical protein		Gene absent
	gcc121	97		hypothetical protein		Gene absent
	gcc122	248		hypothetical protein		Gene absent
	gcc123	98		hypothetical protein		Gene absent
	gcc124	105		hypothetical protein		Gene absent
	gcc125	102		hypothetical protein		Gene absent
	gcc126	197		hypothetical protein		Gene absent
	mdf	1166	K03723	mfd; transcription-repair coupling factor (superfamily II helicase) [EC:3.6.4.-]		Point mutations
	gcc127	386		hypothetical protein		Gene absent
	xre	196		HTH-type transcriptional regulator Xre		Gene absent
	gcc128	62		hypothetical protein		Gene absent
	gcc129	201		hypothetical protein		Gene absent
	gcc130	151		hypothetical protein		Gene absent
	gcc131	111		hypothetical protein		Gene absent
	gcc132	74		hypothetical protein		Gene absent
	gcc133	64		hypothetical protein		Gene absent
	gcc134	91		hypothetical protein		Gene absent
	gcc135	465		hypothetical protein		Gene absent
	gcc136	86		hypothetical protein		Gene absent
	gcc137	186		hypothetical protein		Gene absent
	gcc138	163		hypothetical protein		Gene absent
	gcc139	126		hypothetical protein		Gene absent
	gcc140	96		hypothetical protein		Gene absent
	gcc141	116		hypothetical protein		Gene absent
	acpP	55	K02078	Acyl carrier protein	met	Point mutations
	gcc142	73		hypothetical protein		Truncated protein
CC10						
	gcc101	102		hypothetical protein		Truncated protein
	gcc102	113		hypothetical protein		Gene absent
	gcc103	687		putative licABCH operon regulator		Gene absent
	gatB_1	93	K02774	PTS-Gat-EIIB; PTS system, galactitol-specific IIB component [EC:2.7.1.200]	met/env	Gene absent
	gatC_1	446	K02775	PTS-Gat-EIIC; PTS system, galactitol-specific IIC component	met/env	Gene absent
	rhaD	277	K01629	Rhamnulose-1-phosphate aldolase [EC:4.1.2.19]	met	Gene absent
	gatA	155	K02773	PTS-Gat-EIIA; PTS system, galactitol-specific IIA component [EC:2.7.1.200]	met/env	Gene absent
	gatC_2	444	K02775	PTS-Gat-EIIC; PTS system, galactitol-specific IIC component	met/env	Gene absent
	gatB_2	95	K02774	PTS-Gat-EIIB; PTS system, galactitol-specific IIB component [EC:2.7.1.200]	met/env	Gene absent
	soxS	277		Regulatory protein		Gene absent
	yesO	426	K02027	ABC.MS.S; multiple sugar transport system substrate-binding protein		Gene absent
	lacF	297	K02025	ABC.MS.P; multiple sugar transport system permease protein - Lactose transport system permease protein		Gene absent
	araQ	278	K02026	ABC.MS.P1; multiple sugar transport system permease protein - L-arabinose transport system permease protein		Gene absent
	gcc104	734	K07407	E3.2.1.22B; alpha-galactosidase [EC:3.2.1.22]	met	Gene absent
	galK	391	K00849	Galactokinase [EC:2.7.1.6]	met	Gene absent
	galT	383	K00965	UDPglucose--hexose-1-phosphate uridylyltransferase [EC:2.7.7.12]	met	Gene absent
	galE	332	K01784	UDP-glucose 4-epimerase [EC:5.1.3.2]	met	Synonimous point mutations
	galM	334	K01785	Aldose 1-epimerase [EC:5.1.3.3]	met	Gene absent
	csd2	283	K19118	CRISPR-associated protein		Gene absent
	cas4	224	K07464	CRISPR-associated exonuclease		Gene absent
	cas2	98	K09951	CRISPR-associated protein		Gene absent
	gcc105	70		hypothetical protein		Gene absent
	gcc106	385		hypothetical protein		Truncated protein
	galK_2	317	K00849	Galactokinase [EC:2.7.1.6]	met	Gene absent
	gcc107	58		hypothetical protein		Gene absent
	ydfK	239	K07150	Putative membrane protein		Point mutations
	phoB	218		Phosphate regulon transcriptional regulatory protein		Gene absent
	baeS	447		Signal transduction histidine-protein kinase		Gene absent
	gcc108	176		hypothetical protein		Gene absent
	gcc109	1159		IgA FC receptor		Gene absent
	gcc1010	341		hypothetical protein		Gene absent
	gcc1011	303		hypothetical protein		Gene absent
	gcc1012	501	K06919	Putative DNA primase/helicase		Gene absent
	gcc1013	286		hypothetical protein		Gene absent
	gcc1014	66		hypothetical protein		Gene absent
	gcc1015	103		hypothetical protein		Gene absent
	gcc1016	388		hypothetical protein		Point mutations
	yeiL	215		Regulatory protein		Point mutations
	gcc1017	205	K07078	hypothetical protein		Synonimous point mutations
	gcc1018	252		27-O-demethylrifamycin SV methyltransferase		Truncated protein
	lacC	311	K00917	Tagatose 6-phosphate kinase [EC:2.7.1.144]	met	Truncated protein
CC19						
	mdtH	411	K08152	MFS transporter, DHA1 family, multidrug resistance protein B		Point mutations
	pncC	273	K03742	Nicotinamide-nucleotide amidase - Putative competence-damage inducible protein [EC:3.5.1.42]	met	Point mutations
	gcc191	390		hypothetical protein		Point mutations
	xerC	235		Tyrosine recombinase		Gene absent
	gcc192	76		hypothetical protein		Gene absent
	immR	75		HTH-type transcriptional regulator		Gene absent
	gcc193	122		hypothetical protein		Gene absent
	gcc194	96		hypothetical protein		Gene absent
	ztnA	710	K01534	Cd2+/Zn2+-exporting ATPase - putative cadmium-transporting ATPase [EC:3.6.3.3 3.6.3.5]		Gene absent
	cadC	123	K21903	ArsR family transcriptional regulator, heavy-metals responsive transcriptional repressor - Cadmium resistance transcriptional regulatory protein		Gene absent
	gcc195	100		hypothetical protein		Gene absent
	gcc196	263		hypothetical protein		Gene absent
	gcc197	199		hypothetical protein		Gene absent
	gcc198	69		hypothetical protein		Gene absent
	copA	159	K17686	Cu+-exporting ATPase [EC:3.6.3.54]	env	Gene absent
	gcc199	130		hypothetical protein		Gene absent
	gcc1910	76		hypothetical protein		Gene absent
	gcc1911	164		hypothetical protein		Gene absent
	gcc1912	184		hypothetical protein		Gene absent
	gcc1913	64		hypothetical protein		Gene absent
	gcc1914	197		hypothetical protein		Gene absent
	gcc1915	282		hypothetical protein		Gene absent
	gcc1916	934		N-acetylmuramoyl-L-alanine amidase domain-containing protein		Gene absent
	gcc1917	118		hypothetical protein		Gene absent
	gcc1918	285		hypothetical protein		Gene absent
	gcc1919	81		hypothetical protein		Gene absent
	virD4	606	K03205	Type IV secretion system protein	env	Gene absent
	gcc1920	163		hypothetical protein		Gene absent
	gcc1921	195		hypothetical protein		Gene absent
	gcc1922	78		hypothetical protein		Gene absent
	spxA	128		Regulatory protein		Gene absent
	gcc1923	143		hypothetical protein		Gene absent
	hpaII	452	K00558	DNMT1; DNA (cytosine-5)-methyltransferase 1 - Modification methylase [EC:2.1.1.37]	met	Gene absent
	gcc1924	273		hypothetical protein		Gene absent
	gcc1925	58		hypothetical protein		Gene absent
	mmuP/rocE	459	K16235	S-methylmethionine amino-acid permease		Point mutations
	gcc1926	56		hypothetical protein		Point mutations
	strA	294	K07284	Sortase A [EC:3.4.22.70]		Gene absent
	gcc1927	706		hypothetical protein		Gene absent
CC17						
	gcc171	89		hypothetical protein		Point mutations
	dtpT	491	K03305	Proton-dependent oligopeptide transporter, POT family		Synonimous point mutations
	malR	343	K02529	HTH-type LacI family transcriptional regulator		Point mutations
	glpT	458	K02445	MFS transporter, OPA family, glycerol-3-phosphate transporter	env	Point mutations
	artP	269	K02030	ABC; polar amino acid transport system substrate-binding protein - Arginine-binding extracellular protein	env	Synonimous point mutations
	gcc172	115		hypothetical protein		Gene absent
	gcc173	147		hypothetical protein		Gene absent
	gcc174	68		hypothetical protein		Gene absent
	feuC	334	K02015	ABC; iron complex transport system permease protein - Iron-uptake system permease protein	env	Point mutations
	secY	418	K03076	Accessory Sec system protein translocase subunit	met/env/cell	Gene absent
	asp1	526	K12268	Accessory secretory protein Asp1	env	Gene absent
	asp2	530	K12269	Accessory secretory protein Asp2	env	Gene absent
	asp3	182	K12270	Accessory secretory protein Asp3	env	Gene absent
	gtf1	507		Glycosyltransferase		Gene absent
	gtf2	443		Glycosyltransferase-stabilizing protein		Gene absent
	gcc174	56		hypothetical protein		Gene absent
	wbbI	332		Beta-1,6-galactofuranosyltransferase		Gene absent
	epsJ	571		Putative glycosyltransferase		Gene absent

capA	393	K07282	Gamma-polyglutamate biosynthesis protein - Capsule biosynthesis protein		Point mutations
degV	283		hypothetical protein (contaning DegV domain)		Synonimous point mutations
ysnE	66	K03828	Putative acetyltransferase [EC:2.3.1.-]		Point mutations
efrB	590	K18888	ATP-binding cassette, subfamily B, multidrug efflux pump	env	Point mutations
gdh	450	K00262	Glutamate dehydrogenase (NADP+ specific) [EC:1.4.1.4]	met	Point mutations
yhcR	691	K01081	5'-nucleotidase [EC:3.1.3.5]	met	Point mutations
gldA	363	K00005	Glycerol dehydrogenase [EC:1.1.1.6]	met	Point mutations
gcc175	163	K02755	PTS-Bgl-EIIA; PTS system, beta-glucoside-specific IIA component [EC:2.7.1.-]	env	Point mutations
gcc176	535	K01992	ABC-2 type transport system permease protein	env	Point mutations
gcc177	77		hypothetical protein		Truncated protein
natA	108	K01990	ABC-2 type transport system ATP-binding protein	env	Point mutations
dinG	836	K03722	ATP-dependent DNA helicase [EC:3.6.4.12]	met	Point mutations
nikE	132	K10824	Nickel transport system ATP-binding protein [EC:3.6.3.24]	env	Truncated protein
nikD	263	K15587	Nickel transport system ATP-binding protein [EC:3.6.3.24]	env	Point mutations
gspA	402		General stress protein A		Gene absent
isaA	235		Putative transglycosylase		Truncated protein
lytN	57		hypothetical protein		Synonimous point mutations
gcc178	586	K00382	DLD; dihydrolipoamide dehydrogenase [EC:1.8.1.4]	met/cell	Synonimous point mutations
pdhC	463	K00627	DLAT; pyruvate dehydrogenase E2 component (dihydrolipoamide acetyltransferase) [EC:2.3.1.12]	met	Point mutations
acoA	323	K00161	PDHA; pyruvate dehydrogenase E1 component alpha subunit [EC:1.2.4.1]	met/env	Synonimous point mutations
glgD	107	K00975	glgC; glucose-1-phosphate adenylyltransferase [EC:2.7.7.27]	met/cell	Point mutations
glgC	380	K00975	Glucose-1-phosphate adenylyltransferase	met/cell	Point mutations
metN	245	K02071	Methionine import ATP-binding protein MetN	env	Point mutations
gcc179	230		hypothetical protein		Truncated protein
yhjX	407	K08177	oxlT; MFS transporter, OFA family, oxalate/formate antiporter	env	Point mutations
luxA	348		Alkanal monooxygenase alpha chain		Point mutations
gcc1710	58		hypothetical protein		Gene absent
gcc1711	95		hypothetical protein		Truncated protein
gcc1712	134		hypothetical protein		Gene absent
gcc1713	406		hypothetical protein		Gene absent
gcc1714	359		hypothetical protein		Point mutations
cadR	320		CdaA regulatory protein		Synonimous point mutations
gcc1715	282		hypothetical protein		Gene absent
gcc1716	415		hypothetical protein		Gene absent
lacC	270	K00917	Tagatose 6-phosphate kinase [EC:2.7.1.144]	met	Point mutations
gcc1717	66		FMN-binding protein		Truncated protein
dinB	370	K02346	DNA polymerase IV [EC:2.7.7.7]	met	Point mutations
mta_2	58		hypothetical protein		Point mutations
glpF_1	238	K02440	GLPF; glycerol uptake facilitator protein	env	Point mutations
gcc1718	92		hypothetical protein		Gene absent
endA	118	K15051	DNA-entry nuclease		Gene absent
gcc1719	67		hypothetical protein		Gene absent
panE	318	K00077	2-dehydropantoate 2-reductase [EC:1.1.1.169]	met	Gene absent
gcc1720	235		hypothetical protein		Gene absent
puuD	230	K07010	Putative glutamine amidotransferase	met	Point mutations
yxeM_1	278	K02030	ABC; polar amino acid transport system substrate-binding protein	env	Point mutations
speG_1	195		Spermidine N(1)-acetyltransferase		Gene absent
cas3	480	K07012	CRISPR-associated endonuclease/helicase [EC:3.1.-.- 3.6.4.-]	cell	Gene absent
gcc1721	271		Putative phosphatase		Point mutations
gcc1722	895	K01537	Ca2+-transporting ATPase [EC:3.6.3.8]		Synonimous point mutations
cpdA	102		hypothetical protein		Synonimous point mutations
gpp1723	205		hypothetical protein		Point mutations
gpp1724	288	K07052	hypothetical protein		Point mutations
cssS	419		Sensor histidine kinase		Point mutations
leuS	834	K01869	LARS; leucyl-tRNA synthetase [EC:6.1.1.4]	met	Point mutations
pta	331	K00625	Phosphate acetyltransferase [EC:2.3.1.8]	met	Point mutations
ydfG	197		NADP-dependent 3-hydroxy acid dehydrogenase		Truncated protein
pyrB	308	K00609	Aspartate carbamoyltransferase catalytic subunit [EC:2.1.3.2]	met	Point mutations
smc_1	825		Chromosome partition protein Smc		Truncated protein
gpp1725	177		hypothetical protein		Gene absent
gpp1726	159		hypothetical protein		Gene absent
gpp1727	136		hypothetical protein		Gene absent
gpp1728	89		hypothetical protein		Gene absent
gpp1729	127		hypothetical protein		Gene absent
lytS	580	K07704	Two-component system, LytTR family, sensor histidine kinase [EC:2.7.13.3]	met/env	Point mutations
lytR	486		Transcriptional regulator LytR		Point mutations
aam	680		Acylamidase		Truncated protein
pepS	414	K19689	Aminopeptidase [EC:3.4.11.-]	met	Point mutations
uvrB	664	K03702	Excinuclease ABC subunit B - UvrABC system protein	met	Point mutations
prtP_2	1386		PIII-type proteinase		Gene absent
gcc1730	354		hypothetical protein		Gene absent
mutT	175	K03574	8-oxo-dGTP diphosphatase [EC:3.6.1.55]	met	Truncated protein
gcc1731	56		hypothetical protein		Truncated protein
sigA	370	K03086	RNA polymerase primary sigma factor	met	Synonimous point mutations
gcc1732	91		hypothetical protein		Gene absent
lepB	190	K03100	Signal peptidase I [EC:3.4.21.89]	met/cell	Gene absent
inlA_2	1435		Internalin-A		Gene absent
gcc1733	503		Fimbrial subunit type 1		Gene absent
rgpB	303	K12997	Rhamnosyltransferase [EC:2.4.1.-]	met	Point mutations
scpA	457	K01361	C5a peptidase - lactocepin [EC:3.4.21.96]	met	Truncated protein
strA	292	K07284	Sortase A [EC:3.4.22.70]	met	Gene absent
gcc1734	224		hypothetical protein		Gene absent
rsmH_1	189		hypothetical protein		Point mutations
gcc1735	282		putative NTE family protein		Point mutations
**CC23**					
gcc231	207		hypothetical protein		Gene absent
gcc232	86		hypothetical protein		Gene absent
gcc233	368		hypothetical protein		Gene absent
comR	301	K20342	HTH-type transcriptional regulator, regulator for ComX	cell	Gene absent
yvgN_2	115		putative oxidoreductase		Point mutations
gcc234	132		hypothetical protein		Truncated protein
gcc235	389		hypothetical protein		Gene absent
gcc236	173		hypothetical protein		Gene absent
gcc237	95		hypothetical protein		Gene absent
gcc238	285		hypothetical protein		Gene absent
gcc239	97		hypothetical protein		Gene absent
gcc2310	135		hypothetical protein		Gene absent
gcc2311	119		hypothetical protein		Gene absent
hsdS	462	K01154	Type I restriction enzyme, S subunit - EcoKI specificity protein [EC:3.1.21.3]	cell	Gene absent
gcc2312	524		hypothetical protein		Gene absent
gcc2313	107		hypothetical protein		Point mutations
ampH	384		D-alanyl-D-alanine-carboxypeptidase/endopeptidase		Point mutations
fabG_1	241	K00059	3-oxoacyl-[acyl-carrier protein] reductase [EC:1.1.1.100	met	Synonimous point mutations
cylE	668	K11052	CylE uncharacterised protein	env	Point mutations
gcvT	318	K00605	Aminomethyltransferase [EC:2.1.2.10]	met	Point mutations
fabF_1	732	K09458	3-oxoacyl-[acyl-carrier-protein] synthase II [EC:2.3.1.179]	met	Point mutations
gcc2314	404		hypothetical protein		Point mutations
cylK	192	K11056	CylK uncharacterised protein		Point mutations
gcc2315	162		hypothetical protein		Point mutations
fetA	219	K02068	Putative ABC transport system ATP-binding protein - iron export	env	Synonimous point mutations
gcc2316	65		hypothetical protein		Truncated protein
yxdM	652	K02004	Putative ABC transport system permease protein	env	Point mutations
graS	313		Sensor histidine kinase		Synonimous point mutations
smc_2	537		Chromosome partition protein		Point mutations
gcc2317	143		hypothetical protein		Truncated protein
mntH	523	K03322	Manganese/divalent metal cation transport protein	env	Gene absent
gcc2318	436		hypothetical protein		Gene absent
cfa	253	K11045	cAMP factor	env	Gene absent
dinJ	98	K07473	DNA-damage-inducible protein J	cell	Point mutations
gcc2319	113		hypothetical protein		Gene absent
gcc2320	123		hypothetical protein		Gene absent
gcc2321	141		hypothetical protein		Gene absent
xre_1	113		HTH-type transcriptional regulator		Gene absent
xre_2	105		HTH-type transcriptional regulator		Gene absent
gcc2322	47		hypothetical protein		Gene absent
xre_3	144		HTH-type transcriptional regulator		Gene absent
gcc2324	104		hypothetical protein		Gene absent
gcc2325	154		hypothetical protein		Gene absent
gcc2326	88		hypothetical protein		Gene absent
smc	754		Chromosome partition protein		Gene absent
gcc2327	126		hypothetical protein		Gene absent
xre	75		HTH-type transcriptional regulator		Truncated protein
puuR	118		HTH-type transcriptional regulator		Gene absent
gcc2328	834		hypothetical protein		Gene absent
gcc2329	181		hypothetical protein		Gene absent
gcc2330	125		Transcriptional regulator		Gene absent
irp5	272	K04783	Yersiniabactin salicyl-AMP ligase [EC:6.3.2.-]	met	Gene absent
gcc2331	148		2-hydroxy-7-methoxy-5-methyl-1-naphthoate--CoA ligase		Gene absent
gcc2332	52		hypothetical protein		Gene absent
gcc2333	135		hypothetical protein		Gene absent
htsT	199	K16926	Energy-coupling factor transport system substrate-specific component	env	Gene absent
ecfT	238	K16785	Energy-coupling factor transport system permease protein	env	Gene absent
gcc2334	294	K06147	ABCB-BAC; ATP-binding cassette, subfamily B	env	Gene absent
msbA	292	K06147	ABCB-BAC; ATP-binding cassette, subfamily B - Lipid A export ATP-binding/permease protein	env	Gene absent
ndvA	574	K06147	ABCB-BAC; ATP-binding cassette, subfamily B - Beta-(1-->2)glucan export ATP-binding/permease protein	env	Gene absent
glpT	458	K02445	MFS transporter, OPA family, glycerol-3-phosphate transporter	env	Point mutations
gcc2335	168	K06940	uncharacterized protein		Point mutations
gcc2336	676		hypothetical protein		Truncated protein
vanYB	251	K07260	Zinc D-Ala-D-Ala carboxypeptidase [EC:3.4.17.14]	met/env	Gene absent
